# Visible-Light, Iodine-Promoted Formation of *N*-Sulfonyl Imines and *N*-Alkylsulfonamides from Aldehydes and Hypervalent Iodine Reagents

**DOI:** 10.3390/molecules23081838

**Published:** 2018-07-24

**Authors:** Megan D. Hopkins, Zachary C. Brandeburg, Andrew J. Hanson, Angus A. Lamar

**Affiliations:** Department of Chemistry and Biochemistry, The University of Tulsa, 800 S. Tucker Dr., Tulsa, OK 74104, USA; mdh933@utulsa.edu (M.D.H.); brandeburgz@gmail.com (Z.C.B.); djh758@utulsa.edu (A.J.H.)

**Keywords:** visible-light, iodine promoted, *N*-sulfonyl imine, iodobenzene diacetate (PIDA), sulfonamide, hypervalent iodine, aldimine synthesis, iminoiodinane, nitrogen-centered radical

## Abstract

Alternative synthetic methodology for the direct installation of sulfonamide functionality is a highly desirable goal within the domain of drug discovery and development. The formation of synthetically valuable *N*-sulfonyl imines from a range of aldehydes, sulfonamides, and PhI(OAc)_2_ under practical and mild reaction conditions has been developed. According to mechanistic studies described within, the reaction proceeds through an initial step involving a radical initiator (generated either by visible-light or heat) to activate the reacting substrates. The reaction provides a synthetically useful and operationally simple, relatively mild alternative to the traditional formation of *N*-sulfonyl imines that utilizes stable, widely available reagents.

## 1. Introduction

**Figure 1:**
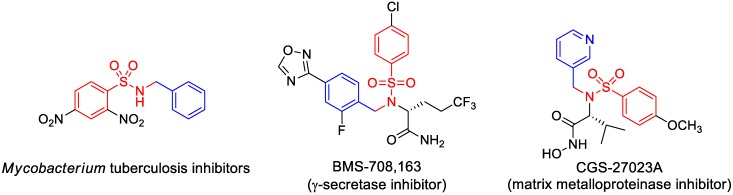
Selected examples of bioactive benzylic *N*-alkylsulfonamide pharmacores.

Sulfonamides are a crucial class of bioisosteric building blocks that are prevalent in a wide range of pharmaceuticals (such as sulfadiazine and amprenavir) and a variety of bioactive compounds (Figure 1) [1,2,3,4,5,6]. The incorporation of an *N*-alkylsulfonamide unit is most frequently accomplished by the reaction of a sulfonyl chloride with a pre-installed alkyl amine in a basic medium. However, the direct installation of an entire *N*-sulfonyl unit provides an attractive option for the drug discovery and development community to synthesize sulfonamides. With regard to *N*-sulfonyl incorporation, recent strategies have devoted attention to the transformation of functionalities other than pre-installed amines (such as aldehyde) to *N*-sulfonyl imines. These intermediate imine species can be easily reduced to *N*-alkylsulfonamides, effectively circumventing the need for a pre-installed amine. In addition, *N*-sulfonyl imines have found use as versatile activated (electron-deficient) electrophiles in a number of organic transformations, including nucleophilic additions (to form chiral amines) [7,8,9,10,11,12,13], cycloadditions [14,15,16,17], aza-Friedel–Crafts [18,19,20,21,22], ene reactions [23,24,25,26], imino-aldol reactions [27,28,29,30], and C–H functionalizations [31,32,33,34,35]. 

**Scheme 1:**
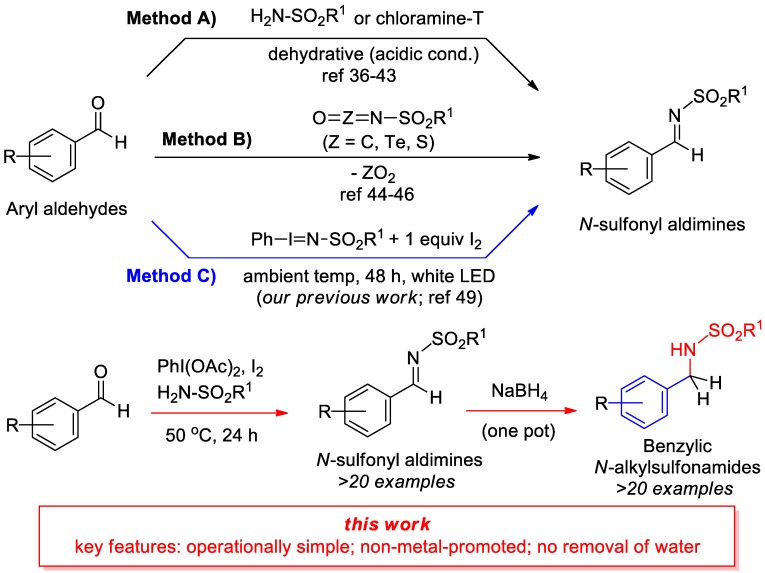
Current methods of *N*-sulfonyl aldimine formation from aryl aldehydes.

Due to the increasing utilization of these electrophilic synthetic intermediates, a variety of methods to prepare *N*-sulfonyl imines have been developed in recent years (Scheme 1). The most frequently employed path for production of an *N*-sulfonyl aldimine involves the condensation of an aldehyde and a sulfonamide (Scheme 1, method A). The inherently low nucleophilicity of sulfonamide has led to disadvantageous conditions that can include (1) the requirement of a harsh Bronsted or Lewis acid, (2) elevated temperatures (>100 °C), (3) difficult purification procedures, and (4) the removal of water (by chemical or physical means) to achieve carbonyl activation [36,37,38,39,40,41,42,43]. In addition to traditional approaches of aldehyde/sulfonamide condensation, methods involving the non-dehydrative reaction of aldehydes with isocyanate analogues have been reported (Scheme 1, method B) [44,45,46]. Resulting from our research group’s interest in the utilization of N-centered radical (NCR) precursors for C–N bond formation [47,48,49], we have recently reported a mild alternative for imine formation that uses iodine and pre-formed iminoiodinane reagents in the presence of light (Scheme 1, method C), which addresses many of the shortcomings of traditional methods [49]. NCR-mediated reactions have seen substantial advances recently, due to the advent of visible-light photoredox catalysis and the development of predictable NCR precursors [50,51,52], but there have been very few reports that have utilized NCR species for carbonyl activation [49,53,54,55]. In order to develop an even more practical and operationally simple method, we aimed to use commercially available, stable reagents such as sulfonamide and (diacetoxyiodo)benzene (referred to as PhI(OAc)_2_ or PIDA) under relatively mild conditions. Herein we disclose a new pathway to produce *N*-sulfonyl imines without the removal of water, using a hypervalent iodine reagent (PIDA) [56], iodine, aldehyde, and sulfonamide, along with a two-step (one-pot) method to form *N*-alkylsulfonamides. According to mechanistic studies, the reaction appears to operate through a unique mechanism that involves a radical initiation step, followed by a more traditional condensation of the sulfonamide unit. 

## 2. Results

**Scheme 2:**
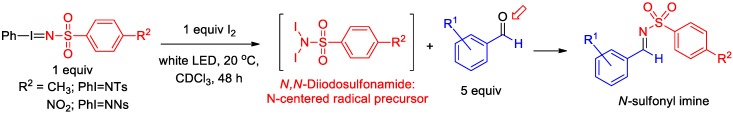
General reaction for the formation of *N*-sulfonyl imines from iminoiodinane/I_2_.

We have previously described the formation of *N*-sulfonyl aldimines, using iminoiodinane reagents and aryl aldehyde reagents, that is believed to proceed through an N-centered radical (NCR) pathway (Scheme 2) [49]. During our initial investigation, *N*,*N*′-diiodosulfonamide, formed from iminoiodinane and I_2_, was isolated and shown to be a likely reactive species in the reaction [49]. *N*,*N*′–Diiodosulfonamide moieties are highly reactive species, in which both of the available N–I bonds can be activated by ambient light without the need for a transition-metal catalyst, UV photoreactor, or external chemical initiator. From experimental observations, it was speculated that an NCR species resulting from *N*,*N*′–diiodosulfonamide was serving as a radical initiator and activator of aldehyde, though a detailed mechanism was unclear at the time. We envisioned the in-situ formation and usage of acetyl hypoiodite (IOAc) (resulting from the addition of I_2_ to PhI(OAc)_2_) [57,58] with a sulfonamide as a different and more convenient initiator in the formation of *N*-sulfonyl imines, thus eliminating the need to synthesize iminoiodinane reagents.

### 2.1. Optimization of Reaction Conditions and Control Reactions

In our initial attempt to form *N*-sulfonyl aldimine, 4-bromobenzaldehyde (**1**) was selected as a representative aldehyde, and tested using PhI(OAc)_2_, I_2_, and a representative sulfonamide (NsNH_2_) following conditions similar to our previously reported iminoiodinane/I_2_ system (Table 1, entry 1) [49]. To our delight, a 34% yield was obtained using 1 equivalent of PhI(OAc)_2_, and was improved to 42% (entry 2) when the amount of PhI(OAc)_2_ was increased to 2 equivalents. It was observed that under ambient temperature (20 °C) conditions, product **2de** does not form in the absence of either PhI(OAc)_2_ or I_2_ (entries 3–5). To determine if the reaction might simply be an acid-mediated condensation of 4-bromobenzaldehyde (**1**) and Ns–NH_2_, an experiment using hydroiodic acid (HI) was conducted at 20 °C, but as shown in entry 6, no product was observed. This result indicates that the reaction is not simply proceeding through a traditional acid-promoted, dehydrative condensation pathway of an aldehyde and sulfonamide at ambient temperature.

As shown in Table 1, entry 7, the efficiency of the reaction is improved upon heating to 50 °C. In entries 8–10, the stoichiometry of the aldehyde was varied, and interestingly, an excess of >2 equivalents is necessary to provide **2de** in good yield. 

Using optimized reaction conditions, a series of additional control experiments were conducted (Table 2). At 50 °C, light appears to be an unnecessary component of the reaction, as shown in entries 1 and 5, in which a similar yield was obtained when compared to the standard reaction (entry 1). The absence of I_2_ in the reaction was shown to be detrimental, though formation of **2de** did occur at 50 °C, as shown in entry 2. This is in stark contrast to the reaction at 20 °C, in which the absence of I_2_ resulted in no formation of **2de**. Additionally, the reaction is severely affected when activated molecular sieves are used to remove water during the course of the reaction (entry 4). Finally, the addition of 1 equivalent of 2,2,6,6-tetramethyl-1-piperidinyloxy (TEMPO), a known radical inhibitor, resulted in a substantial decrease in product yield (entry 6), indicating that a radical species might be present in the mechanistic pathway.

To further investigate the observed difference in reactivity (and dependence upon I_2_) at 20 and 50 °C, additional experiments were conducted at 30 and 40 °C in the presence and absence of 1 equivalent I_2_. The results of these experiments are shown in Table 3. Reactions conducted below 50 °C appear to operate through an iodine-dependent, light-promoted pathway, whereas at 50 °C, enough thermal energy may be available to facilitate the reaction with or without the formation of an N–I bond. 

### 2.2. Substrate Scope

**Scheme 3:**
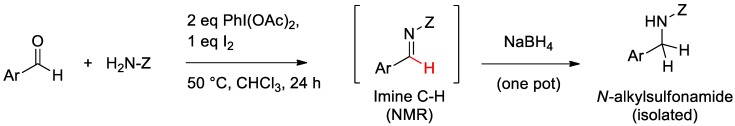
Two-step (one-pot) formation of *N*-alkylsulfonamides from aldehydes and sulfonamide.

Most *N*-sulfonyl imines can be isolated either by crystallization or silica gel chromatography, though in our past experience, successive recrystallizations may be required in order to remove the excess aldehyde, thus lowering the isolated yield. For this reason, we have determined the yields of *N*-sulfonyl imines via ^1^H-NMR integration, with an internal standard by focusing upon the distinctive *N*-sulfonyl imine C–H proton (Scheme 3) [49]. In order to further verify the formation of *N*-sulfonyl imines, we have also developed a two-step (one-pot) reduction of *N*-sulfonyl imine products, using excess NaBH_4_ (Scheme 3). The resulting *N*-alkylsulfonamides represent an additional important class of synthetic targets within the drug discovery and development community. In fact, within the array of approximately 60 pharmaceuticals marketed in the United States that contain a sulfonamide group, there are 18 drugs that contain an *N*-alkylsulfonamide unit as a key molecular feature [59]. 

Using the optimized reaction conditions (5 equivalents aldehyde, 2 equivalents PhI(OAc)_2_, and 1 equivalent I_2_ at 50 °C and 24 h in chloroform), a survey of the substrate scope with regard to aldehyde and sulfonamide was investigated (Table 4). In entries 1–13, a range of aldehydes were varied against a relatively electron-rich sulfonamide, Ts-NH_2_. The effect of the electronic nature of the aldehyde was investigated by using a variety of electron-donating and withdrawing substituents. Though a relatively small preference for an electron-rich aldehyde substrate was observed regarding the formation of *N*-sulfonyl imines (entries 1–7), the reaction consistently produced moderate to good yields of imines (**2**) and *N*-alkylsulfonamides (**3**) (see experimental Supporting Information for spectra). A number of functionalities are tolerated, including aryl ether, aryl halide, nitro, cyano, and benzylic C–H bonds.

In Table 4, entries 14–23, a variety of different sulfonamide substrates were tested. Yields relatively consistent with Ts-NH_2_ were obtained using sulfonamide substrates, such as 4-Cl-PhSO_2_NH_2_ and PhSO_2_NH_2_ (entries 14–18). In reactions that employed less electron-rich sulfonamides, the yields were negatively impacted (entries 20–23) relative to Ts-NH_2_. In addition, sulfonamides with ortho substituents on the aryl ring also performed poorly relative to their para-substituted analogues (entries 19 and 23). The practicality and operational simplicity of the system offers a complementary strategy to the use of iminoiodinanes for the introduction of a variety of *N*-sulfonyl units directly from commercially available sulfonamides. 

### 2.3. Investigation of Mechanism

To determine the likely mechanistic pathway of the imine-forming transformation, additional experiments were performed, in an attempt to differentiate between a traditional carbonyl/amine condensation and a radical-facilitated activation of the aldehyde.

#### 2.3.1. Deuterium-Labeling Experiment

**Scheme 4:**
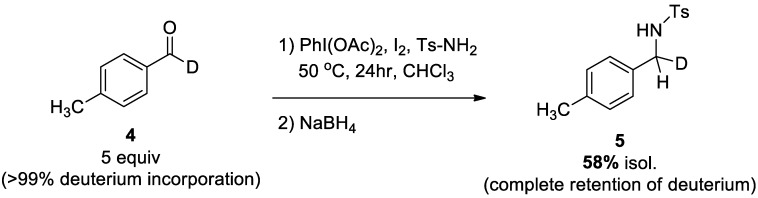
Mechanistic probe experiment using deuterium-labeled aldehyde.

Initially, it was hypothesized that the reaction mechanism would proceed through a pathway in which an acyl radical is initially formed by abstraction of the aldehydic C–H by a radical initiator, such as acetyl hypoiodite (IOAc), or an NCR species generated from the sulfonamide. To determine if the aldehydic C–H was abstracted, a deuterated aldehyde (**4**) was prepared according to literature precedent, and reacted under the optimized reaction conditions with Ts-NH_2_ (Scheme 4) [49,60]. Following the two-step reduction procedure with NaBH_4_, a 58% yield of the *N*-alkylsulfonamide product (**5**) was isolated, with complete retention of the C–D bond. This result indicates that the C–N bond-forming step may not involve an acyl radical species, and is much more likely operating through a traditional nucleophilic condensation of the sulfonamide and aldehyde. However, an acyl radical species cannot be eliminated from a plausible mechanism, because this result is complicated by the requirement of an excess of aldehyde substrate in order to obtain an observable amount of product.

#### 2.3.2. Isolation of Mechanistic Intermediates

**Scheme 5:**
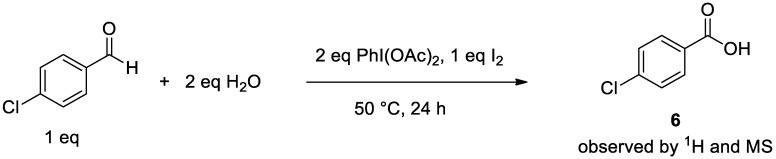
Conversion of aldehyde to carboxylic acid with H_2_O under PhI(OAc)_2_/I_2_ conditions.

In an effort to understand the requirement for an excess of aldehyde substrate, we sought to determine whether or not additional products may be formed from the aldehyde substrate under the reaction conditions. Under the optimized reaction conditions for producing *N*-sulfonyl imines, no precautions are taken to remove water prior to or during the reaction (in fact, removal of water is detrimental, as shown in Table 2, entry 4). In addition, a broad peak in the vicinity of a carboxylic acid O–H proton was observed in several crude reactions when quantifying yields by ^1^H-NMR. With this in mind, an experiment was designed to investigate the possible formation of a carboxylic acid byproduct under standard conditions in the presence of water (Scheme 5). The formation of 4-chlorobenzoic acid (**6**) was observed under standard reaction conditions (in the absence of sulfonamide) using 1 equivalent of aldehyde. This oxidation is likely occurring through abstraction of the aldehydic C–H to form an intermediary acyl radical species, via either an IOAc radical initiator or a *N*-centered radical produced in situ from an iodinated sulfonamide. 

**Scheme 6:**
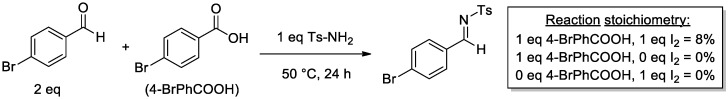
4-BrPhCOOH-promoted condensation of aldehyde and sulfonamide.

Following this observation, an investigation was conducted regarding the ability of 4-bromobenzoic acid to serve as an acidic agent in the promotion of a sulfonamide/aldehyde condensation, to form an *N*-sulfonyl imine. As shown in Scheme 6, the condensation of Ts-NH_2_ and 4-bromobenzaldehyde is promoted by 4-bromobenzoic acid (albeit with low efficiency), but the condensation does not occur in the absence of 4-bromobenzoic acid, and interestingly, in the absence of I_2_. The condensation is not simply promoted by I_2_ serving as a Lewis acid either, as shown by the lack of imine formation in the presence of I_2_ and the absence of 4-bromobenzoic acid. This implies that IOAc or I_2_ could play a role in the activation of both the aldehyde and the sulfonamide. In fact, the formation of a sulfonamide N–I bond occurs under similar conditions in a number of reported systems [61,62,63,64,65,66,67]. These results further validate the importance of the oxidizing agent PIDA in the system, but also provide support for the potential role of benzoic acid formed as a byproduct and potentially important component in the mechanistic pathway of the reaction.

## 3. Discussion

### 3.1. Trends in Reactivity

In our previously reported system, using iminoidinane reagents with I_2_ at room temperature, a clear preference was observed for aldehyde substrates with electron-donating groups [49]. In addition, the most effective iminoiodinane was consistently the electron-deficient PhI = NNs [49]. These trends are no longer seen in this study when using PIDA/I_2_ and sulfonamide. A slight preference for electron-rich aldehydes may be present, but it is not significant (Table 4, entries 1–7). The sulfonamide species, however, appears to play an important role in the present system. The reaction appears to be hindered by electron-deficient sulfonamides, or those that contain ortho-substituted aryl rings. This appears to be more indicative of a traditional role of nucleophilic sulfonamide in a condensation reaction. With regard to the in-situ reduction to *N*-alkylsulfonamide species, some reductions may appear to be more facile than others; however, we believe at this time that the inconsistent conversion of various *N*-sulfonyl imines to their respective *N*-alkylsulfonamide products is simply an artifact of the isolation of the resulting *N*-alkylsulfonamides. Some examples required multiple chromatographic separations of the benzyl alcohol (from reduction of the excess aldehyde substrate) and benzyl amine, and the relatively lower yields are simply a result of the removal of benzyl alcohol after reduction of the excess aldehyde.

### 3.2. Proposed Mechanism 

**Scheme 7:**
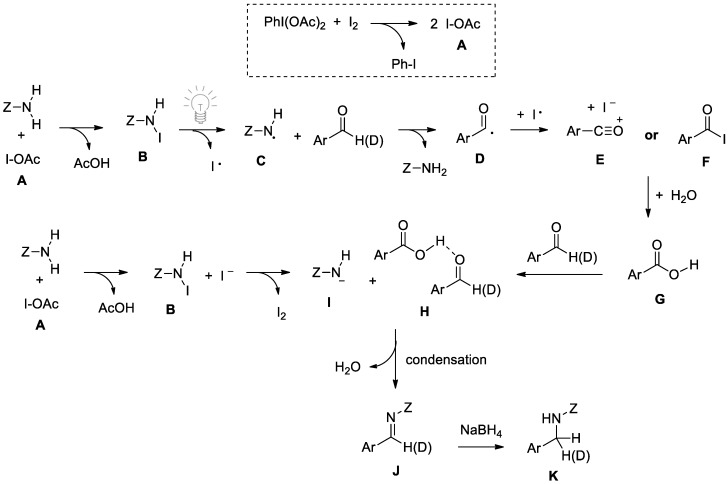
Proposed mechanism of I_2_-promoted *N*-sulfonyl formation.

The plausible mechanism presented in Scheme 7 attempts to account for the following experimental observations: (1) a decrease in imine production in the presence of either a radical inhibitor (TEMPO) or molecular sieves; (2) the requirement of PIDA and an excess of >2 equivalents of aldehyde; (3) the complete retention of the aldehydic C–H (D) in the imine or *N*-alkylsulfonamide product; (4) the observed oxidation of an aldehyde to a carboxylic acid using PIDA/I_2_/H_2_O; and (5) the likely involvement of an R–N(I)H species, as indicated by the requirement of I_2_ in the acid-promoted condensation in Scheme 5.

Upon generation of acetyl hypoiodite **A** from PIDA and I_2_, the sulfonamide is iodinated to produce NCR precursor **B**. Activated either by visible light (room temperature) or heating at 50 °C, the NCR species **C** forms and facilitates C–H (D) abstraction from a sacrificial equivalent of aldehyde, to produce a relatively stable acyl radical **D**. This acyl radical species can either combine with or be oxidized by the iodine radical to form species **E** or **F**, which would readily form the carboxylic acid **G** in the presence of water. This acid can then aid in a condensation reaction with a second equivalent of aldehyde, shown as **H**. In order to generate a more nucleophilic sulfonamide species, it is plausible that a second equivalent of the R-N(I)H species **B** will form from sulfonamide and IOAc, to produce a more reactive species **I** by attack from iodide (which would in turn regenerate I_2_). The condensation of nucleophilic **I** with an aldehyde activated by a carboxylic acid **H** would result in complete retention of the aldehydic C–H (D) bond, and also produce the water needed for the production of carboxylic acid **G**. This mechanistic pathway is unconventional in the sense that it involves both a radical activation step and a traditional nucleophilic condensation step.

## 4. Materials and Methods 

### 4.1. Chemicals and Instruments

All reagents and solvents were purchased from commercial sources and used without further purification. I_2_ was purchased from Alfa Aesar (Tewksbury, MA, USA) in 99.99+% purity (metals basis). A description of the construction of our light bath (LED) photochamber is provided in the Supporting Information. ^1^H- and ^13^C-NMR spectra were recorded on a Varian 400/100 (400 MHz) spectrometer (Agilent Technologies, Palo Alto, CA, USA) in deuterated chloroform (CDCl_3_), with the solvent residual peak as internal reference unless otherwise stated (CDCl_3_: ^1^H = 7.26 ppm, ^13^C = 77.23 ppm). Data are reported in the following order: Chemical shifts (δ) are reported in ppm, and spin–spin coupling constants (*J*) are reported in Hz, while multiplicities are abbreviated by s (singlet), d (doublet), dd (doublet of doublets), t (triplet), dt (double of triplets), td (triplet of doublets), and m (multiplet). Infrared spectra were recorded on a Nicolet iS50 FT-IR spectrometer (ThermoFisher Scientific, Waltham, MA, USA), and peaks are reported in reciprocal centimeters (cm^−1^). Melting points (M.p.) were recorded on a Mel-Temp II (Laboratory Devices) and were uncorrected. Accurate mass spectrum (HRMS—High Resolution Mass Spectrometry) was performed using a Thermo Scientific Exactive spectrometer (Waltham, MA, USA) operating in positive ion electrospray mode (ESI–electrospray ionization). 

### 4.2. Synthetic Procedures

#### 4.2.1. General Procedure for the Preparation of *N*-Sulfonyl Imines (**2**) (^1^H-NMR Yields)

To an oven-dried reaction tube was added aldehyde (1.25 mmol, 5 equivalents), PhI(OAc)_2_ (0.50 mmol, 2 equivalents), sulfonamide (0.25 mmol, 1 equivalent), I_2_ (0.25 mmol, 1 equivalents), and CDCl_3_ (3 mL). The mixture was stirred at 50 °C under argon for 24 h. After 24 h, the reaction was cooled to room temperature and an internal standard was added (1,4-dimethoxybenzene, 0.25 mmol, 35 mg); the mixture then was stirred for an additional 5–10 min. The percent yield was calculated against the internal standard via ^1^H-NMR integrations of the imine C–H signal and the aromatic (4H) signal of the internal standard.

#### 4.2.2. General Procedure for the Preparation of *N*-Alkylsulfonamides (**3**)

To an oven-dried flask was added aldehyde (2.5 mmol, 5 equivalents), sulfonamide (0.5 mmol, 1 equivalents), PhI(OAc)_2_ (1.0 mmol, 2 equivalents), I_2_ (0.5 mmol, 1 equivalent) in CHCl_3_ (6 mL). The mixture was stirred at 50 °C under argon for 24 h. After 24 h, the reaction was cooled to room temperature and solvent was removed using a rotary evaporator. The crude was dissolved in 12 mL of a 1:1 MeOH/DCM mixture, and placed in an ice bath to cool to 0 °C. NaBH_4_ (0.312 g, ~8 mmol) was added in small portions. After addition, the mixture was removed from the ice bath and stirred at room temperature for an additional 45 min. After 45 min, the reaction was quenched with 12 mL H_2_O and then extracted with EtOAc (2 × 20 mL). The combined organic layers were washed with brine (20 mL), dried (Na_2_SO_4_), and filtered, and the filtrate solvent was removed by evaporation. The resulting crude *N*-alkylsulfonamide **3** was purified by silica gel flash chromatography, using a hexanes:EtOAc eluent. 

#### 4.2.3. Isotopically Labeled Experiment

Deuterated aldehyde **4** was prepared according to a known procedure from ester reduction with LiAlD_4_, followed by Dess–Martin oxidation to the aldehyde [49,60]. To an oven-dried reaction tube was added the deuterated aldehyde (0.625 mmol, 0.074 mL), *p*-toluenesulfonamide (0.125 mmol, 0.0214 g), iodobenzene diacetate (0.250 mmol, 0.0805 g), and I_2_ (0.125 mmol, 0.0317 g), all in CHCl_3_ (1.5 mL). The mixture was stirred at 50 °C under argon for 24 h in a white LED chamber. After 24 h, the solvent was evaporated, and CDCl_3_ was added to record a ^1^H-NMR of the crude mixture. The title compound **5** was isolated from this crude mixture with a 58% yield following the general procedure for preparation of *N*-alkylsulfonamides: white solid (20 mg, 58%).; m.p. 86–88 °C; purification (hexanes:EtOAc, 70:30); R_f_ = 0.59; ^1^H-NMR (400 MHz, CDCl_3_) δ = 7.76 (d, *J* = 8.0 Hz, 2H), 7.31 (d, *J* = 8.0 Hz, 2H), 7.08 (s, 4H), 4.64 (d, *J* = 4.0 Hz, 1H), 4.05 (d, *J* = 4.0 Hz, 1H), 2.44 (s, 3H), and 2.31 (s, 3H) ppm; ^13^C-NMR (100 MHz, CDCl_3_); δ 143.5, 137.7, 136.8, 133.1, 129.7, 129.3, 127.9, 127.2, 46.8, 21.5, and 21.1 ppm; IR (neat) with ν = 3262, 2923, 1597, 1320, 1152, 1092, 726, and 665 cm^−1^. HRMS (ESI) calculated for C_15_H_17_N_1_O_2_S_1_D_1_ [M + H]^+^ requires *m/z* 277.11365, and *m/z* 277.11075 was found.

#### 4.2.4. Observation of Carboxylic Acid Intermediate

To an oven-dried round bottom flask was added 4-chlorobenzaldehyde (1 equivalent, 1 mmol), iodobenzene diacetate (2 equivalent, 2 mmol), I_2_ (1 equivalent, 1 mmol), H_2_O (2 equivalent, 2 mmol) in CHCl_3_ (12 mL). A reflux condenser was attached and the reaction was stirred at 50 °C for 24 h. After the reaction time, 7 mL of 1 M NaOH solution was added to the reaction mixture. The aqueous portion was then separated and acidified using 4 mL of a 15% HCl solution. Compound **6** was then extracted with EtOAc (3 × 7 mL). The combined organic layers were dried with Na_2_SO_4_ and the solvent evaporated. Characterization of **6** was made in comparison to known spectral data.

#### 4.2.5. Carboxylic Acid-Promoted Condensation

To an oven-dried reaction tube was added 4-bromobenzoic acid (1 equivalent, 0.125 mmol), 4-bromobenzaldehyde (2 equivalents, 0.25 mmol), *p*-toluenesulfonamide (1 equivalent, 0.125 mmol), and I_2_ (1 equivalent, 0.125 mmol) in 1.5 mL of CDCl_3_. The reaction was stirred at 50 °C for 24 h. The reaction was then cooled to room temperature, an internal standard (1,4-dimethoxybenzene) was added, and the reaction mixture was directly analyzed by ^1^H-NMR. 

### 4.3. Characterization Data

Characterization via ^1^H-NMR of all intermediate *N*-sulfonyl imines was accomplished using comparisons to reported spectra, and an additional table of imine C–H peaks is included in the ESI. All *N*-alkylsulfonamides were isolated according to general procedure. Products **3aa** [68], **3ae** [69], **3ba** [68], **3bb** [70], **3bf** [71], **3ca** [68], **3da** [72], **3ea** [68], **3eb** [70], **3ee** [69], **3ha** [73], **3hb** [74], **3ia** [75], **3ja** [73], **3ka** [73], **3la** [72], and **3ma** [72] have been reported, and the characterizational data was in agreement with literature values (spectra and full characterization available in ESI). Additional *N*-alkylsulfonamides display the characterizational data shown below. 

#### 4.3.1. 4-Chloro-*N*-[(4-methylphenyl)methyl]-benzenesulfonamide (**3bc**)

This was prepared according to the general procedure: white solid (59 mg, 40%); M.*p*. 120–122 °C; purification (hexanes:EtOAc, 70:30); R_f_ = 0.58; ^1^H-NMR (400 MHz, CDCl_3_) δ = 7.77 (d, *J* = 8.0 Hz, 2H), 7.45 (d, *J* = 8.0 Hz, 2H), 7.06 (m, 4H), 4.86 (t, *J* = 4.0 Hz, 1H), 4.10 (d, *J* = 4.0Hz, 2H), and 2.31 (s, 3H) ppm; ^13^C-NMR (100 MHz, CDCl_3_) δ = 139.1, 138.5, 137.9, 132.8, 129.4, 129.3, 128.6, 127.8, 47.1, and 21.1 ppm; IR (neat) with ν = 3245, 2923, 1586, 1325, 1158, 1092, 1058, 757, and 565 cm^−1^. HRMS (ESI) calculated for C_14_H_15_N_1_O_2_S_1_Cl_1_ [M + H]^+^ requires *m/z* 296.05120, and *m/z* 296.05029 was found.

#### 4.3.2. 2-Chloro-*N*-[(4-methylphenyl)methyl]-benzenesulfonamide (**3bd**)

This was prepared according to the general procedure: white solid (25 mg, 17%); M.*p*. 64–66 °C; purification (hexanes:EtOAc, 70:30); R_f_ = 0.53; ^1^H-NMR (400 MHz, CDCl_3_) δ = 8.09 (d, *J* = 8.0 Hz, 1H), 7.50–7.49 (m, 2H), 7.42–7.38 (m, 1H), 7.06 (s, 4H), 5.21 (t, *J* = 4.0 Hz, 1H), 4.07 (d, *J* = 4.0 Hz, 2H), and 2.29 (s, 3H) ppm; ^13^C-NMR (100 MHz, CDCl_3_) δ 137.8, 137.1, 133.6, 132.6, 131.5, 131.4, 131.3, 129.3, 127.9, 127.2, 47.3, and 21.1 ppm; IR (neat) with ν = 3277, 3094, 2924, 1426, 1327, 1163, 1042, 760, and 585 cm^−1^. HRMS (ESI) calculated for C_14_H_15_N_1_O_2_S_1_Cl_1_ [M + H]^+^ requires *m/z* 296.05120, and *m/z* 296.05140 was found.

#### 4.3.3. *N*-[(4-Bromophenyl)methyl]-4-chlorobenzenesulfonamide (**3dc**)

This was prepared according to the general procedure on a 0.125 mmol scale: white solid (25 mg, 54%); M.*p*. 126–128 °C; purification (hexanes:EtOAc, 70:30); R_f_ = 0.64; ^1^H-NMR (400 MHz, CDCl_3_) δ = 7.77 (d, *J* = 8.0 Hz, 2H), 7.48 (d, *J* = 8.0 Hz, 2H), 7.41 (d, *J* = 8.0 Hz, 2H), 7.08 (d, *J* = 8.0 Hz, 2H), 4.82 (t, *J* = 4.0 Hz, 1H), and 4.11 (d, *J* = 4.0 Hz, 2H) ppm; ^13^C-NMR (100 MHz, CDCl_3_) δ 139.4, 138.3, 135.0, 131.8, 129.50, 129.45, 128.5, 122.0, and 46.6 ppm; IR (neat) with ν = 3246, 3087, 1573, 1320, 1153, 826 cm^−1^; HRMS (ESI) calculated for C_13_H_12_N_1_O_2_S_1_Cl_1_Br_1_ [M + H]^+^ requires *m/z* 361.94607, and *m/z* 361.94302 was found.

#### 4.3.4. 4-Methyl-*N*-[(4-nitrophenyl)methyl]-benzenesulfonamide (**3fa**) 

This was prepared according to the general procedure: yellow solid (62 mg, 40%); M.*p*. 111–113 °C; purification (hexanes:EtOAc, 60:40); R_f_ = 0.47. ^1^H-NMR (400 MHz, CDCl_3_) δ = 8.12 (d, *J* = 8.0 Hz, 2H), 7.74 (d, *J* = 8.0 Hz, 2H), 7.40 (d, *J* = 8.0 Hz, 2H), 7.30 (d, *J* = 8.0 Hz, 2H), 5.13 (t, *J* = 4.0 Hz, 1H), 4.24 (d, *J* = 4.0 Hz, 2H), and 2.44 (s, 3H) ppm. ^13^C-NMR (100 MHz, CDCl_3_) δ 144.0, 143.9, 136.6, 129.9, 128.4, 127.1, 128.8, 46.4, 27.5, and 21.6 ppm; IR (neat) with ν = 3252, 2855, 1516, 1309, 1150, 1109, and 814 cm^−1^. HRMS (ESI) calculated for C_14_H_15_N_2_O_4_S_1_ [M + H]^+^ requires *m/z* 307.07525, and *m/z* 307.07452 was found.

#### 4.3.5. *N*-[(4-Cyanophenyl)methyl]-4-methylbenzenesulfonamide (**3ga**)

This was prepared according to the general procedure: white solid (63 mg, 44%); M.*p*. 120–126 °C; purification (hexanes:EtOAc, 60:40); R_f_ = 0.38. ^1^H-NMR (400 MHz, CDCl_3_) δ = 7.72 (d, *J* = 8.0 Hz, 2H), 7.53 (d, *J* = 8.0 Hz, 2H), 7.35 (d, *J* = 8.0 Hz, 2H), 7.29 (d, *J* = 8.0 Hz, 2H), 5.43 (t, *J* = 4.0 Hz, 1H), 4.17 (d, *J* = 4.0 Hz, 2H), and 2.44 (s, 3H) ppm; ^13^C-NMR (100 MHz, CDCl_3_) δ 143.9, 142.0, 136.6, 132.3, 129.8, 128.3, 127.0, 118.5, 111.5, 46.6, and 21.5 ppm; IR (neat) with ν = 3234, 2922, 2857, 2230, 1594, 1324, 1152, 1069, 842, 815, and 548 cm^−1^. HRMS (ESI) calculated for C_15_H_15_N_2_O_2_S_1_ [M + H]^+^ requires *m/z* 287.08543, and *m/z* 287.08475 was found.

## 5. Conclusions

A relatively mild and operationally simple transformation of aldehydes to *N*-sulfonyl imines using commercially available reagents has been described. At room temperature, the reaction is promoted by visible light and does not require removal of water. A variety of *N*-sulfonyl aldimines and benzylic *N*-alkylsulfonamides (resulting from a two-step, one-pot reduction) have been prepared from a range of aryl aldehydes and sulfonamides with moderate to good yields. A mechanistic investigation has provided evidence for a radical-initiated activation of a sacrificial equivalent of aldehyde, in order to produce a carboxylic acid in the presence of water, which in turn facilitates a condensation reaction between an activated sulfonamide and a second equivalent of aldehyde. Further investigation of analogous functional groups and reacting N-species is currently underway. 

## Figures and Tables

**Table 1:**
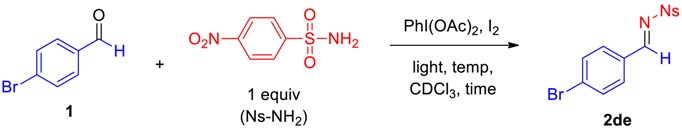
Optimization and control reactions for the PhI(OAc)_2_/I_2_ formation of *N*-sulfonyl imines.

Entry	Equiv. 1	Equiv. PhI(OAc)_2_	Equiv. I_2_	Temp (^°^C)	Time (h)	Light	Additive	Yield 2de (%) ^1^
1	5	1	1	20	48	White LED	None	34
2	5	2	1	20	48	White LED	None	42
3	5	0	1	20	48	White LED	None	0
4	5	1	0	20	48	White LED	None	0
5	5	0	0	20	48	White LED	None	0
6	5	0	0	20	48	White LED	0.55 eq HI	0
7	5	1	1	50	24	Ambient ^2^	None	57
8	5	2	1	50	24	Ambient	None	72
9	2	2	1	50	24	Ambient	None	8
10	1	2	1	50	24	Ambient	None	1
11	5	2	1	50	8	Ambient	None	65
12	5	2	1	50	4	Ambient	None	59

^1^ Yields are based upon ^1^H-NMR integration with 1,4-dimethoxybenzene as internal standard. ^2^ “Ambient” light means no additional light irradiation was used beyond what is found in an illuminated laboratory setting. Reactions were conducted in a fume hood with a 30 W fluorescent bulb approximately 1 m overhead.

**Table 2:**
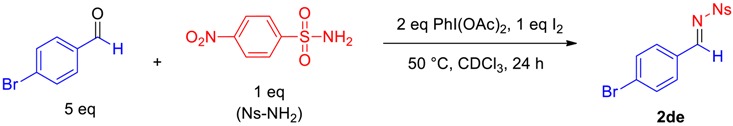
Control reactions for the iminoiodinane/I_2_ formation of *N*-sulfonyl imines.

Entry	Variation from Standard Conditions	Yield 2de (%) ^1^
1	NONE	72
2	0 equiv. I_2_	40
3	0.2 equiv. I_2_	66
4	4A Molecular sieves	8
5	Reaction in dark ^2^	74
6	1 equiv. TEMPO	19

^1^ Yields are based upon ^1^H-NMR integration with internal standard. ^2^ Reaction vessel was wrapped in aluminum foil and the experiment was performed in a laboratory with all lights turned off.

**Table 3:**
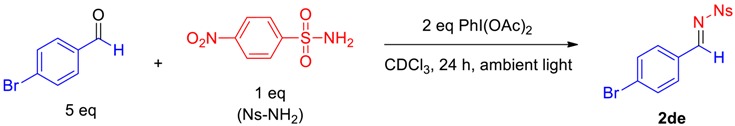
Temperature dependence of the PhI(OAc)_2_-promoted *N*-sulfonyl imine formation in the presence (and absence) of I_2_.

Entry	Oil Bath Temperature (°C)	Eq. I_2_	Yield 2de (%) ^1^
1	20	0	0
2	20	1	39
3	30	0	0
4	30	1	42
5	40	0	5
6	40	1	72
7	50	0	40
8	50	1	72

^1^ Yields are based upon ^1^H-NMR integration with internal standard.

**Table 4:**
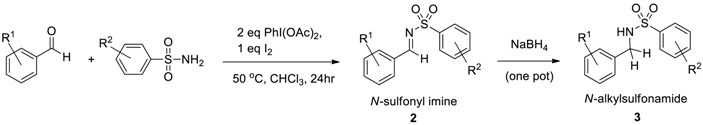
Substrate scope of *N*-sulfonyl imine **2** and *N*-alkylsulfonamide **3** formation.

Entry	(Aldehyde) R^1^	(Sulfonamide) R^2^	Product 2 Yield (%) ^1^	Product 3 Yield (%) ^2^
1	4-OCH_3_	4-CH_3_	**2aa** 61	**3aa** 61
2	4-CH_3_	4-CH_3_	**2ba** 71	**3ba** 60
3	H	4-CH_3_	**2ca** 67	**3ca** 50
4	4-Br	4-CH_3_	**2da** 54	**3da** 45
5	4-Cl	4-CH_3_	**2ea** 65	**3ea** 74
6	4-NO_2_	4-CH_3_	**2fa** 50	**3fa** 40
7	4-CN	4-CH_3_	**2ga** 52	**3ga** 44
8	3-CH_3_	4-CH_3_	**2ha** 70	**3ha** 33
9	2-CH_3_	4-CH_3_	**2ia** 60	**3ia** 33
10	3-OCH_3_	4-CH_3_	**2ja** 60	**3ja** 40
11	2-OCH_3_	4-CH_3_	**2ka** 65	**3ka** 40
12	3-Br	4-CH_3_	**2la** 50	**3la** 22
13	2-Br	4-CH_3_	**2ma** 36	**3ma** 30
14	4-CH_3_	H	**2bb** 69	**3bb** 40
15	4-Cl	H	**2eb** 66	**3eb** 70
16	2-CH_3_	H	**2hb** 60	**3hb** 38
17	4-CH_3_	4-Cl	**2bc** 60	**3bc** 40
18	4-Br	4-Cl	**2dc** 58	**3dc** 54
19	4-CH_3_	2-Cl	**2bd** 35	**3bd** 17
20	4-OCH_3_	4-NO_2_	**2ae** 34	**3ae** 11
21	4-Cl	4-NO_2_	**2ee** 27	**3ee** 21
22	4-CH_3_	3-NO_2_	**2bf** 25	**3bf** 8
23	4-CH_3_	2-NO_2_	**2bg** 6	**3bg** trace

^1^ Yields are based upon ^1^H-NMR integration with internal standard. ^2^ Isolated yields.

## Supplementary Materials

Supplementary materials including spectral characterization are available online.
