# Supplementary material for: Visible-Light, Iodine-Promoted Formation of N-Sulfonyl Imines and N-Alkylsulfonamides from Aldehydes and Hypervalent Iodine Reagents
Source: Molecules. 2018 Jul 24;23(8):1838. doi: 10.3390/molecules23081838 (PMC6222766; doi:10.3390/molecules23081838)

<u>**Experimental Supporting Information**</u>

Visible-Light, Iodine-Promoted Formation of *N*-Sulfonyl Imines and *N*-Alkylsulfonamides from Aldehydes and Hypervalent Iodine Reagents

Megan D. Hopkins, Zachary C. Brandeburg, Andrew J. Hanson, and Angus A. Lamar*

Department of Chemistry and Biochemistry, The University of Tulsa, 800 S. Tucker Dr., Tulsa, OK 74104, USA.

angus-lamar@utulsa.edu

Index

General information - LED chamber S-1

Table of *N*-sulfonyl imine C-H peaks S-2

Characterization of compounds S-2

^{1}H and ^{13}C NMR Spectra of *N*-alkylsulfonamides S-8

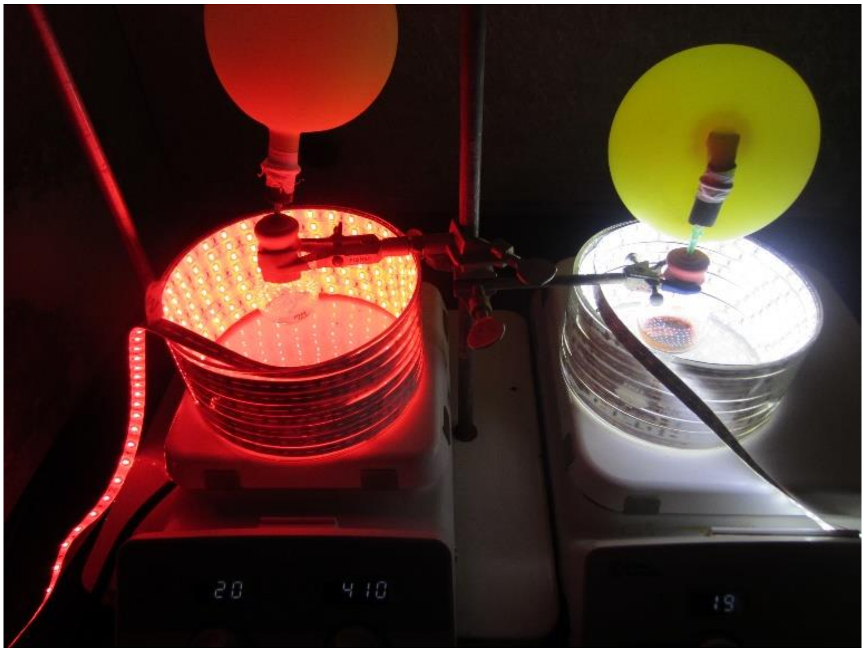

On the left – A red LED strip (620 nm) light bath reaction vessel

On the right – A white LED strip (cool white) light bath reaction vessel

Photocatalytic reactions were set up in a light bath which was constructed in our laboratory as follows. Waterproof 5050 LED strips (12V with power adapter, 18 LEDs/foot, approximately 0.24 Watt per LED – 72 Watt per strip) are coiled around the interior of evaporating dish (170mm x 90mm) using the adhesive backing of the LED strip. A Petri dish (150 x 20 mm) is placed upside down at the bottom of the dish to serve as an elevated glass "floor" to ensure that a round-bottom flask receives maximum light exposure. The temperature inside the dish is monitored and is generally maintained (air-cooled) between 19-22 °C (the temperature has not been observed above 25 °C).

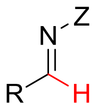

Product	δ imine C-H (ppm) NMR intgn crude	lit. (ref.)
2aa	8.92	8.91 (1)
2ba	8.97	8.96 (1)
2ca	9.02	9.01 (1)
2da	8.93	8.94 (1)
2ea	8.94	8.96 (1)
2fa	9.07	9.07 (1)
2ga	9.01	9.01 (1)
2ha	8.97	8.97 (1)
2ia	9.31	9.30 (1)
2ja	8.95	8.94 (1)
2ka	9.49	9.48 (1)
2la	8.92	8.92 (1)
2ma	9.35	9.37 (1)

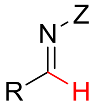

Product	δ imine C-H (ppm) NMR intgn crude	lit. (ref.)
2bb	9.04	9.03 (3)
2eb	9.01	9.02 (3)
2hb	9.36	9.38 (4)
2bc	8.98	8.93 (1)
2dc	8.99	8.95 (1)
2bd	9.13	-
2ae	8.97	8.98 (1)
2ee	9.04	9.04 (1)
2bf	9.06	-
2bg	8.98	9.04 (2)
2de	9.09	9.09 (1)

All values are reported in $CDCl_3$

_N_-(4-methoxybenzyl)-4-methylbenzenesulfonamide (3aa)[5]

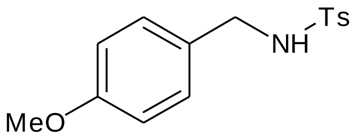

White solid (90 mg, 61% yield). M.p. 119-121 °C. Purification (hexanes:EtOAc, 60:40). R_f = 0.56. ^{1}H NMR (400 MHz, $CDCl_3$) δ = 7.75 (d, *J* = 8.0 Hz, 2H), 7.30 (d, *J* = 8.0 Hz, 2H), 7.10 (d, *J* = 8.0 Hz), 6.79 (d, *J* = 8.0 Hz, 2H), 4.72 (t, *J* = 4.0 Hz, 1H), 4.04 (d, *J* = 4.0 Hz, 2H), 3.77 (s, 3H), 2.43 (s, 3H) ppm. ^{13}C NMR (100 MHz, $CDCl_3$) δ 159.3, 143.4, 136.8, 129.7, 129.2, 128.2, 127.2, 114.0, 55.3, 46.8, 21.5 ppm. IR (neat): ν = 3248, 2922, 1612, 1513, 1320, 1251, 1155, 1029, 815, 553 cm^{-1}. HRMS (ESI): calculated for $C_{15}H_{18}N_1O_3S_1$ $[M + H]^+$ requires *m/z* 292.10074, found *m/z* 292.07983.

4-methyl-_N_-(4-methylbenzyl)benzenesulfonamide (smaller scale, 0.125 mmol) (3ba)[5]

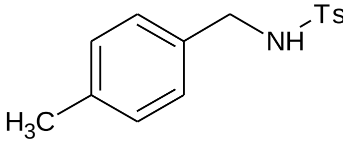

White solid (21 mg, 60%). M.p. 88-90 °C. Purification (hexanes:EtOAc, 70:30). R_f = 0.50. ^{1}H NMR (400 MHz, $CDCl_3$) δ = 7.76 (d, *J* = 8.0 Hz, 2H), 7.31 (d, *J* = 8.0 Hz, 2H), 7.08 (s, 4H), 4.62 (t, *J* = 6.0 Hz, 1H), 4.07 (d, *J* = 6.0 Hz, 2H), 2.44 (s, 3H), 2.31 (s, 3H) ppm. ^{13}C NMR (100 MHz, $CDCl_3$) δ 143.5, 137.7, 136.8, 133.1, 129.7, 129.3, 127.8, 127.2, 47.0, 21.5, 21.1 ppm. IR (neat): ν = 3261, 2921, 1597, 1323, 1151, 1093, 748, 665 cm^{-1}. HRMS (ESI): calculated for $C_{15}H_{18}N_1O_2S_1$ $[M + H]^+$ requires *m/z* 276.10583, found *m/z* 276.10449.

***N*-benzyl-4-methylbenzenesulfonamide (3ca)**[5]

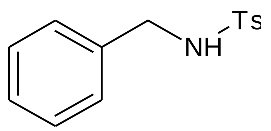

White solid (66 mg, 50%). M.p. 114-116 °C. Purification (hexanes:EtOAc, 60:40). R_f = 0.71. ^{1}H NMR (400 MHz, $CDCl_3$) δ = 7.74 (d, *J* = 8.0 Hz, 2H), 7.29 (d, *J* = 8.0 Hz, 2H), 7.30-7.24 (m, 3H), 7.20-7.18 (m, 2H), 4.84 (t, *J* = 4.0 Hz, 1H), 4.11 (d, *J* = 4.0 Hz, 2H), 2.43 (s, 3H) ppm. ^{13}C NMR (100 MHz, $CDCl_3$) δ 143.5, 136.8, 136.2, 129.7, 128.7, 127.9, 127.8, 127.2, 47.2, 21.5 ppm. IR (neat): ν = 3268, 3064, 1598, 1453, 1321, 1160, 1058, 874, 805, 741 cm^{-1}. HRMS (ESI): calculated for $C_{14}H_{16}N_1O_2S_1$ $[M + H]^+$ requires *m/z* 262.09018, found *m/z* 262.08948.

***N*-(4-bromobenzyl)-4-methylbenzenesulfonamide (3da)**[6]

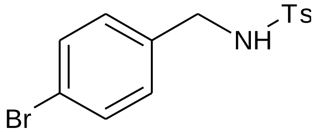

White solid (78 mg, 45%). M.p. 113-115 °C. Purification (hexanes:EtOAc, 60:40). R_f = 0.69. ^{1}H NMR (400 MHz, $CDCl_3$) δ = 7.71 (d, *J* = 8.0 Hz, 2H), 7.36 (d, *J* = 8.0 Hz, 2H), 7.28 (d, *J* = 8.0 Hz, 2H), 7.07 (d, *J* = 8.0 Hz, 2H), 5.17 (t, *J* = 4.0 Hz, 1H), 4.06 (d, *J* = 4.0 Hz, 2H), 2.43 (s, 3H) ppm. ^{13}C NMR (100 MHz, $CDCl_3$) δ 143.7, 136.7, 135.4, 131.7, 129.7, 129.5, 127.1, 121.7, 46.5, 21.5 ppm. IR (neat): ν = 3269, 2920, 1597, 1317, 1155, 1072, 1013, 878, 802, 544 cm^{-1}. HRMS (ESI): calculated for $C_{14}H_{15}N_1O_2S_1Br_1$ $[M + H]^+$ requires *m/z* 342.00069, found *m/z* 341.99771.

***N*-(4-chlorobenzyl)-4-methylbenzenesulfonamide (smaller scale, 0.125 mmol) (3ea)**[5]

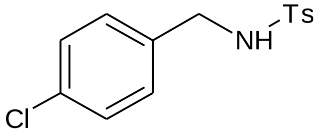

White solid (27 mg, 74%). M.p. 105-107 °C. Purification (hexanes:EtOAc, 60:40). R_f = 0.68. ^{1}H NMR (400 MHz, $CDCl_3$) δ = 7.72 (d, *J* = 8.0 Hz, 2H), 7.29 (d, *J* = 8.0 Hz, 2H), 7.22 (d, *J* = 8.0 Hz, 2H), 7.13 (d, *J* = 8.0 Hz, 2H), 4.81 (t, *J* = 4.0 Hz, 1H), 4.08 (d, *J* = 4.0 Hz, 2H), 2.43 (s, 3H) ppm. ^{13}C NMR (100 MHz, $CDCl_3$) δ 143.7, 136.7, 134.8, 133.7, 129.8, 129.2, 128.8, 127.1, 46.5, 21.5 ppm. IR (neat): ν = 3226, 2920, 1597, 1304, 1183, 816 cm^{-1}. HRMS (ESI): calculated for $C_{14}H_{15}N_1O_2S_1Cl_1$ $[M + H]^+$ requires *m/z* 296.05120, found *m/z* 296.05054.

4-methyl-*N*-(4-nitrobenzyl)benzenesulfonamide (3fa)

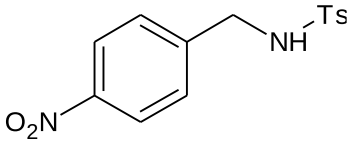

Yellow solid (62 mg, 40%). M.p. 111-113 °C. Purification (hexanes:EtOAc, 60:40). R_f = 0.47. ^{1}H NMR (400 MHz, $CDCl_3$) δ = 8.12 (d, *J* = 8.0 Hz, 2H), 7.74 (d, *J* = 8.0 Hz, 2H), 7.40 (d, *J* = 8.0 Hz, 2H), 7.30 (d, *J* = 8.0 Hz, 2H), 5.13 (t, *J* = 4.0 Hz, 1H), 4.24 (d, *J* = 4.0 Hz, 2H), 2.44 (s, 3H) ppm. ^{13}C NMR (100 MHz, $CDCl_3$) δ 144.0, 143.9, 136.6, 129.9, 128.4, 127.1, 123.8, 46.4, 27.5, 21.6 ppm. IR (neat): ν = 3252, 2855, 1516, 1309, 1150, 1109, 814 cm^{-1}. HRMS (ESI): calculated for $C_{14}H_{15}N_2O_4S_1$ $[M + H]^+$ requires *m/z* 307.07525, found *m/z* 307.07452.

***N*-(4-cyanobenzyl)-4-methylbenzenesulfonamide (3ga)**

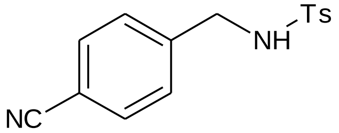

White solid (63 mg, 44%). M.p. 120-126 °C. Purification (hexanes:EtOAc, 60:40). R_f = 0.38. ^{1}H NMR (400 MHz, $CDCl_3$) δ = 7.72 (d, *J* = 8.0 Hz, 2H), 7.53 (d, *J* = 8.0 Hz, 2H), 7.35 (d, *J* = 8.0 Hz, 2H), 7.29 (d, *J* = 8.0 Hz, 2H), 5.43 (t, *J* = 4.0 Hz, 1H), 4.17 (d, *J* = 4.0 Hz, 2H), 2.44 (s, 3H) ppm. ^{13}C NMR (100 MHz, $CDCl_3$) δ 143.9, 142.0, 136.6, 132.3, 129.8, 128.3, 127.0, 118.5, 111.5, 46.6, 21.5 ppm. IR (neat): ν = 3234, 2922, 2857, 2230, 1594, 1324, 1152, 1069, 842, 815, 548 cm^{-1}. HRMS (ESI): calculated for $C_{15}H_{15}N_2O_2S_1$ $[M + H]^+$ requires *m/z* 287.08543, found *m/z* 287.08475.

4-methyl-*N*-(3-methylbenzyl)benzenesulfonamide (3ha)[8]

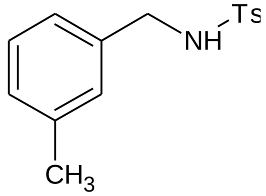

White solid (46 mg, 33%). M.p. 64-66 °C. Purification (hexanes:EtOAc, 60:40). R_f = 0.81. ^{1}H NMR (400 MHz, $CDCl_3$) δ = 7.76 (d, *J* = 8.0 Hz, 2H), 7.31 (d, *J* = 8.0 Hz, 2H), 7.16 (t, *J* = 8.0 Hz, 1H), 7.06 (d, *J* = 8.0 Hz, 1H), 6.98 (m, 2H), 4.60 (t, *J* = 4.0 Hz, 1H), 4.09 (d, *J* = 4.0 Hz, 2H), 2.44 (s, 3H), 2.28 (s, 3H). ^{13}C NMR (100 MHz, $CDCl_3$) δ 143.5, 138.4, 136.8, 136.1, 129.7, 128.62, 128.59, 128.56, 127.2, 124.9, 47.3, 21.5, 21.2 ppm. IR (neat): ν = 3294, 3029, 2922, 1596, 1320, 1153, 1065, 908, 811, 664, 537 cm^{-1}. HRMS (ESI): calculated for $C_{15}H_{18}N_1O_2S_1$ $[M + H]^+$ requires *m/z* 276.10583, found *m/z* 276.10513.

4-methyl-*N*-(2-methylbenzyl)benzenesulfonamide (smaller scale, 0.125 mmol) (3ia)[7]

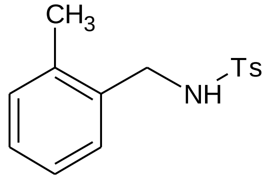

White solid (23 mg, 33%). M.p. 113-117 °C. Purification (hexanes: EtOAc, 60:40). R_f = 0.74. ^{1}H NMR (400 MHz, $CDCl_3$) δ = 7.77 (d, *J* = 8.0 Hz, 2H), 7.32 (d, *J* = 8.0 Hz, 2H), 7.21-7.10 (m, 4H), 4.46 (t, *J* = 4.0 Hz, 1H), 4.09 (d, *J* = 4.0 Hz, 2H), 2.44 (s, 3H), 2.24 (s, 3H) ppm. ^{13}C NMR (100 MHz, $CDCl_3$) δ 143.5, 136.7, 136.6, 133.8, 130.6, 129.7, 128.8, 128.2, 127.2, 126.2, 45.4, 21.5, 18.8 ppm. IR (neat): ν = 3260, 3023, 2925, 1597, 1489, 1423, 1318, 1152, 1091, 1041, 879, 806, 702, 654 cm^{-1}. HRMS (ESI): calculated for $C_{15}H_{18}N_1O_2S_1$ $[M + H]^+$ requires *m/z* 276.10583, found *m/z* 276.10483.

***N*-(3-methoxybenzyl)-4-methylbenzenesulfonamide (smaller scale, 0.125 mmol) (3ja)[8]**

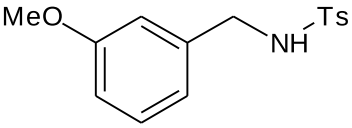

Clear oil (15 mg, 40%). Purification (hexanes:EtOAc, 60:40). R_f = 0.15. ^{1}H NMR (400 MHz, $CDCl_3$) δ = 7.76 (d, *J* = 8.0 Hz, 2H), 7.31 (d, *J* = 8.0 Hz, 2H), 7.19 (t, *J* = 8.0 Hz, 1H), 6.80-6.75 (m, 2H), 6.73 (s, 1H), 4.65 (m, 1H), 4.10 (d, *J* = 6.0 Hz, 2H), 3.75 (s, 3H), 2.44 (s, 3H) ppm. ^{13}C NMR (100 MHz, $CDCl_3$) δ 159.8, 143.6, 137.8, 136.8, 129.8, 129.7, 127.2, 120.0, 113.7, 113.1, 55.2, 47.3, 21.5 ppm. IR (neat): ν = 3279, 2922, 1597, 1586, 1490, 1455, 1436, 1320, 1288, 1262, 1152, 1091, 1040, 864, 812, 780, 660 cm^{-1}. HRMS (ESI): calculated for $C_{15}H_{18}N_1O_3S_1$ $[M + H]^+$ requires *m/z* 292.10074, found *m/z* 292.09961.

***N*-[(2-Methoxyphenyl)methyl]-4-methylbenzenesulfonamide (3ka)[8]**

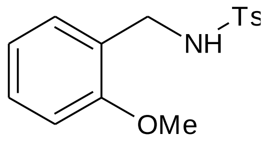

White solid (57 mg, 40%). M.p. 79-81 °C. Purification (hexanes:EtOAc, 70:30). R_f = 0.33. ^{1}H NMR (400 MHz, $CDCl_3$) δ = 7.66 (d, *J* = 8.0 Hz, 2H), 7.21-7.16 (m, 3H), 7.06 (d, *J* = 8.0 Hz, 1H), 6.80 (t, *J* = 8.0 Hz, 1H), 6.73 (d, *J* = 8.0 Hz, 1H), 5.09 (t, *J* = 4.0 Hz, 1H), 4.14 (d, *J* = 4.0 Hz, 2H), 3.73 (s, 3H), 2.38 (s, 3H) ppm. ^{13}C NMR (100 MHz, $CDCl_3$) δ 157.2, 143.0, 129.8, 129.4, 129.2, 127.03, 126.99, 124.3, 120.5, 110.1, 55.1, 44.0, 21.5 ppm. IR (neat): ν = 3304, 3254, 3014, 2949, 2837, 1602, 1404, 1325, 1148, 1024, 756, 663 cm^{-1}. HRMS (ESI): calculated for $C_{15}H_{18}N_1O_3S_1$ $[M + H]^+$ requires *m/z* 292.10074, found *m/z* 292.09958.

***N*-(3-bromobenzyl)-4-methylbenzenesulfonamide (3la)[6]**

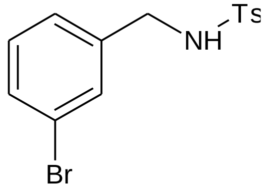

White solid (36 mg, 22%). M.p. 78-80 °C. Purification (hexanes:EtOAc, 60:40). R_f = 0.57. ^{1}H NMR (400 MHz, $CDCl_3$) δ =7.73 (d, *J* = 8.0 Hz, 2H), 7.37 (td, *J* = 4.0 Hz, 1H), 7.31 (d, *J* = 8.0 Hz, 2H), 7.27 (m, 1H), 7.15 (d, *J* = 4.0 Hz, 2H), 4.72 (t, *J* = 4.0 Hz, 1H), 4.11 (d, *J* = 4.0 Hz, 2H), 2.44 (s, 3H) ppm. ^{13}C NMR (100 MHz, $CDCl_3$) δ 143.8, 138.5, 136.7, 131.0, 130.8, 130.2, 129.8, 127.1, 126.4, 122.7, 46.6, 21.5 ppm. IR (neat): ν = 3259, 3067, 2918, 2852, 1570, 1435, 1316, 1149, 1052, 921, 814, 692, 529 cm^{-1}. HRMS (ESI): calculated for $C_{14}H_{15}N_1O_2S_1Br_1$ $[M + H]^+$ requires *m/z* 342.00069, found *m/z* 341.99731.

_N_-(2-bromobenzyl)-4-methylbenzenesulfonamide (3ma)[6]

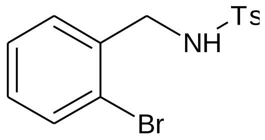

Yellow oil (50 mg, 30%). Purification (hexanes:EtOAc, 80:20). R_f = 0.27. ^{1}H NMR (400 MHz, $CDCl_3$) δ = 7.71 (d, *J* = 8.0 Hz, 2H), 7.45 (d, *J* = 8.0 Hz, 1H), 7.30 (d, *J* = 8.0 Hz, 1H), 7.25 (d, *J* = 8.0 Hz, 2H), 7.21 (t, *J* = 8.0 Hz, 1H), 7.10 (t, *J* = 8.0 Hz, 1H), 5.03 (t, *J* = 6.0 Hz, 1H), 4.22 (d, *J* = 6.0 Hz, 2H), 2.41 (s, 3H) ppm. ^{13}C NMR (100 MHz, $CDCl_3$) δ 136.9, 135.5, 132.7, 130.5, 129.6, 129.5, 129.1, 127.7, 127.1, 123.4, 47.4, 21.5 ppm. IR (neat): ν = 3282, 2922, 1597, 1441, 1325, 1154, 1024, 811, 658 cm^{-1}. HRMS (ESI): calculated for $C_{14}H_{15}N_1O_2S_1Br_1$ $[M + H]^+$ requires *m/z* 342.00069, found *m/z* 341.99728.

_N_-(4-methylbenzyl)benzenesulfonamide (3bb)[11]

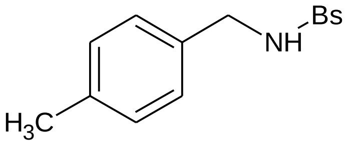

White solid (52 mg, 40%). M.p. 76-79 °C. Purification (hexanes:EtOAc, 70:30). R_f = 0.48. ^{1}H NMR (400 MHz, $CDCl_3$) δ = 7.87 (d, *J* = 8.0 Hz, 2H), 7.59 (tt, *J* = 8.0 Hz, 4.0 Hz, 1H), 7.51 (m, 2H), 7.06 (s, 4H), 4.75 (t, *J* = 4.0 Hz, 1H), 4.09 (d, *J* = 4.0 Hz, 2H), 2.30 (s, 3H) ppm. ^{13}C NMR (100 MHz, $CDCl_3$) δ 139.9, 137.7, 133.1, 132.7, 129.3, 129.1, 127.8, 127.1, 47.1, 21.1 ppm. IR (neat): ν = 3264, 2920, 1615, 1445, 1318, 1156, 1038, 907, 805 cm^{-1}. HRMS (ESI): calculated for $C_{14}H_{16}N_1O_2S_1$ $[M + H]^+$ requires *m/z* 262.09018, found *m/z* 262.08905.

_N_-(4-chlorobenzyl)benzenesulfonamide (smaller scale, 0.125 mmol) (3eb)[9]

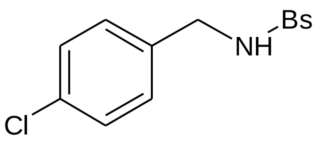

White solid (51 mg, 70%). M.p. 109-112 °C. Purification (hexanes:EtOAc, 70:30). R_f = 0.44. ^{1}H NMR (400 MHz, $CDCl_3$) δ = 7.84 (d, *J* = 8.0 Hz, 2H), 7.61-7.57 (m, 1H), 7.51 (d, *J* = 8.0 Hz, 2H), 7.22 (d, *J* = 8.0 Hz, 2H), 7.12 (d, *J* = 8.0 Hz, 2H), 4.87 (t, *J* = 4.0 Hz, 1H), 4.11 (d, *J* = 4.0 Hz, 2H) ppm. ^{13}C NMR (100 MHz, $CDCl_3$) δ 139.8, 134.7, 133.8, 132.8, 129.2, 128.8, 127.0, 46.6, 29.7 ppm. IR (neat): ν = 3255, 3059, 1598, 1490, 1320, 1181, 1091, 817 cm^{-1}. HRMS (ESI): calculated for $C_{13}H_{13}N_1O_2S_1Cl_1$ $[M + H]^+$ requires *m/z* 282.03555, found *m/z* 282.03458.

_N_-(2-methylbenzyl)benzenesulfonamide (smaller scale, 0.125 mmol) (3hb)[12]

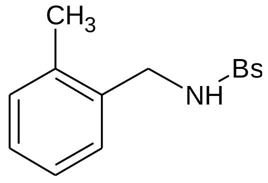

White solid (25 mg, 38%). M.p. 117-123 °C. Purification (hexanes:EtOAc, 60:40). R_f = 0.65. ^{1}H NMR (400 MHz, $CDCl_3$) δ = 7.88 (d, *J* = 8.0 Hz, 2H), 7.61-7.57 (m, 1H), 7.53 (d, *J* = 8.0 Hz, 2H), 7.21-7.09 (m, 4H), 4.56 (t, *J* = 4.0 Hz, 1H), 4.12 (d, *J* = 4.0 Hz, 2H), 2.23 (s, 3H) ppm. ^{13}C NMR (100 MHz, $CDCl_3$) δ 139.6, 133.7, 132.7, 130.6, 129.3, 129.1, 128.9, 128.3, 127.1, 126.2, 45.4, 18.8 ppm. IR (neat): ν = 3274, 3068, 1448, 1323, 1159, 1092, 1036, 886, 722, 686 cm^{-1}. HRMS (ESI): calculated for $C_{14}H_{16}N_1O_2S_1$ $[M + H]^+$ requires *m/z* 262.09018, found *m/z* 262.08923.

4-chloro-_N_-(4-methylbenzyl)benzenesulfonamide (3bc)

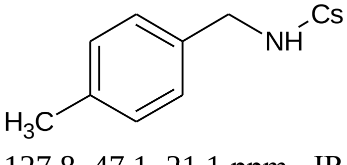

White solid (59 mg, 40%). M.p. 120-122 °C. Purification (hexanes:EtOAc, 70:30). R_f = 0.58. ^{1}H NMR (400 MHz, $CDCl_3$) δ = 7.77 (d, *J* = 8.0 Hz, 2H), 7.45 (d, *J* = 8.0 Hz, 2H), 7.06 (m, 4H), 4.86 (t, *J* = 4.0 Hz, 1H), 4.10 (d, *J* = 4.0Hz, 2H), 2.31 (s, 3H) ppm. ^{13}C NMR (100 MHz, $CDCl_3$) δ 139.1, 138.5, 137.9, 132.8, 129.4, 129.3, 128.6, 127.8, 47.1, 21.1 ppm. IR (neat): ν = 3245, 2923, 1586, 1325, 1158, 1092, 1058, 757, 565 cm^{-1}. HRMS (ESI): calculated for $C_{14}H_{15}N_1O_2S_1Cl_1$ $[M + H]^+$ requires *m/z* 296.05120, found *m/z* 296.05029.

***N*-(4-bromobenzyl)-4-chlorobenzenesulfonamide (smaller scale, 0.125 mmol) (3dc)**

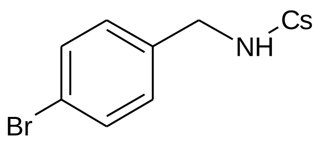

White solid (25 mg, 54%). M.p. 126-128 °C. Purification (hexanes:EtOAc, 70:30). R_f = 0.64. ^{1}H NMR (400 MHz, $CDCl_3$) δ = 7.77 (d, *J* = 8.0 Hz, 2H), 7.48 (d, *J* = 8.0 Hz, 2H), 7.41 (d, *J* = 8.0 Hz, 2H), 7.08 (d, *J* = 8.0 Hz, 2H), 4.82 (t, *J* = 4.0 Hz, 1H), 4.11 (d, *J* = 4.0 Hz, 2H) ppm. ^{13}C NMR (100 MHz, $CDCl_3$) δ 139.4, 138.3, 135.0, 131.8, 129.50, 129.45, 128.5, 122.0, 46.6 ppm. IR (neat): ν = 3246, 3087, 1573, 1320, 1153, 826 cm^{-1}. HRMS (ESI): calculated for $C_{13}H_{12}N_1O_2S_1Cl_1Br_1$ [M + H]$^+$ requires *m/z* 361.94607, found *m/z* 361.94302.

2-chloro-*N*-(4-methylbenzyl)benzenesulfonamide (3bd)

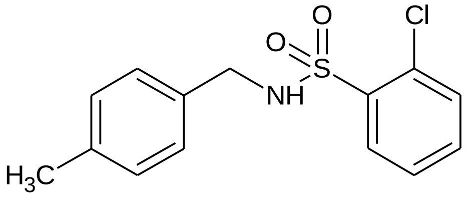

White solid (25 mg, 17%). M.p. 64-66 °C. Purification (hexanes:EtOAc, 70:30). R_f = 0.53. ^{1}H NMR (400 MHz, $CDCl_3$) δ = 8.09 (d, *J* = 8.0 Hz, 1H), 7.50-7.49 (m, 2H), 7.42-7.38 (m, 1H), 7.06 (s, 4H), 5.21 (t, *J* = 4.0 Hz, 1H), 4.07 (d, *J* = 4.0 Hz, 2H), 2.29 (s, 3H) ppm. ^{13}C NMR (100 MHz, $CDCl_3$) δ 137.8, 137.1, 133.6, 132.6, 131.5, 131.4, 131.3, 129.3, 127.9, 127.2, 47.3, 21.1 ppm. IR (neat): ν = 3277, 3094, 2924, 1426, 1327, 1163, 1042, 760, 585 cm^{-1}. HRMS (ESI): calculated for $C_{14}H_{15}N_1O_2S_1Cl_1$ [M + H]$^+$ requires *m/z* 296.05120, found *m/z* 296.05140.

***N*-(4-methoxybenzyl)-4-nitrobenzenesulfonamide (3ae)[9]**

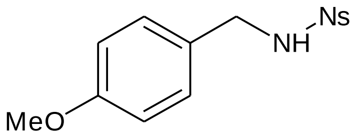

Tan solid (19 mg, 11%). M.p. 126-130 °C. Purification (hexanes:EtOAc, 60:40). R_f = 0.53. ^{1}H NMR (400 MHz, $CDCl_3$) δ = 8.31 (d, *J* = 8.0 Hz, 2H), 8.09 (d, *J* = 8.0 Hz, 2H), 7.08 (d, *J* = 8.0 Hz, 2H), 6.77 (d, *J* = 8.0 Hz, 2H), 4.82 (t, *J* = 4.0 Hz, 1H), 4.16 (d, *J* = 4.0 Hz, 2H), 3.76 (s, 3H) ppm. ^{13}C NMR (100 MHz, $CDCl_3$) δ 146.1, 129.3, 128.6, 128.3, 127.4, 124.3, 121.0, 114.2, 55.3, 47.0 ppm. IR (neat): ν = 3269, 3110, 29`5, 2840, 1512, 1245, 1151, 1030, 817 cm^{-1}. HRMS (ESI): calculated for $C_{14}H_{15}N_2O_5S_1$ [M + H]$^+$ requires *m/z* 323.07017, found *m/z* 323.22165.

***N*-(4-chlorobenzyl)-4-nitrobenzenesulfonamide (smaller scale, 0.125 mmol) (3ee)[11]**

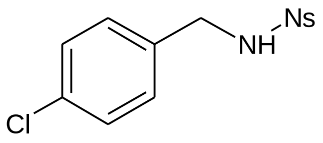

Oil (9 mg, 21%). Purification (hexanes:EtOAc, 60:40). R_f = 0.62. ^{1}H NMR (400 MHz, $CDCl_3$) δ = 8.35 (d, *J* = 8.0 Hz, 2H), 8.02 (d, *J* = 8.0 Hz, 2H), 7.27 (d, *J* = 8.0 Hz, 2H), 7.15 (d, *J* = 8.0 Hz, 2H), 4.84 (t, *J* = 4.0 Hz, 1H), 4.21 (d, *J* = 4.0 Hz, 2H) ppm. ^{13}C NMR (100 MHz, $CDCl_3$) δ 145.9, 134.3, 134.0, 129.2, 129.0, 128.3, 124.4, 46.7, 29.7 ppm. IR (neat): ν = 3265, 2921, 1606, 1341, 1156, 837 cm^{-1}. HRMS (ESI): calculated for $C_{13}H_{12}N_2O_4S_1Cl_1$ [M + H]$^+$ requires *m/z* 327.02063, found *m/z* 327.07776.

***N*-(4-methylbenzyl)-3-nitrobenzenesulfonamide (3bf)[10]**

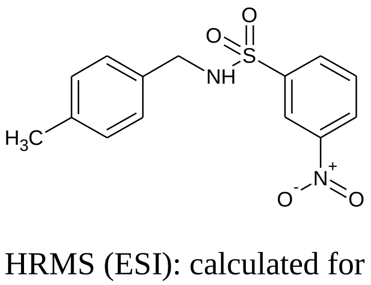

White solid (12 mg, 8%). M.p. 110-113 °C. Purification (hexanes:EtOAc, 70:30). R_f = 0.36. ^{1}H NMR (400 MHz, $CDCl_3$) δ = 8.57 (t, *J* = 4.0 Hz, 1H), 8.38 (ddd, *J* = 4.0 Hz, 8.0 Hz, 1H), 8.13 (dt, *J* = 8.0 Hz, 1H), 7.67 (t, *J* = 8.0 Hz, 1H), 7.04 (s, 4H), 4.93 (t, *J* = 4.0 Hz, 1H), 4.21 (d, *J* = 4.0 Hz, 2H), 2.28 (s, 3H) ppm. ^{13}C NMR (100 MHz, $CDCl_3$) δ 142.6, 138.1, 132.5, 130.3, 129.4, 127.9, 127.1, 126.9, 122.4, 47.2, 21.0 ppm. IR (neat): ν = 3250, 3089, 2921, 1607, 1526, 1428, 1331, 1163 cm^{-1}. HRMS (ESI): calculated for $C_{14}H_{15}N_2O_4S_1$ [M + H]$^+$ requires *m/z* 307.07525, found *m/z* 307.07416.

References:

1. Hopkins, M. D.; Scott, K. A.; DeMier, B. C.; Morgan, H. R.; Macgruder, J. A.; Lamar, A. A., Formation of N-sulfonyl imines from iminoiodinanes by iodine-promoted, N-centered radical sulfonamidation of aldehydes. *Organic & biomolecular chemistry* **2017,** *15* (43), 9209-9216.
2. Castellano, S.; Fiji, H. D. G.; Kinderman, S. S.; Watanabe, M.; de Leon, P.; Tamanoi, F.; Kwon, O., Small-Molecule Inhibitors of Protein Geranylgeranyltransferase Type I. *Journal of the American Chemical Society* **2007,** *129* (18), 5843-5845.
3. Chen, D.; Chen, X.; Du, T.; Kong, L.; Zhen, R.; Zhen, S.; Wen, Y.; Zhu, G., Highly efficient and diastereoselective synthesis of 1,3-oxazolidines featuring a palladium-catalyzed cyclization reaction of 2-butene-1,4-diol derivatives and imines. *Tetrahedron Letters* **2010,** *51* (39), 5131-5133.
4. Kohler, M. C.; Yost, J. M.; Garnsey, M. R.; Coltart, D. M., Direct Carbon−Carbon Bond Formation via Soft Enolization: A Biomimetic Asymmetric Mannich Reaction of Phenylacetate Thioesters. *Organic letters* **2010,** *12* (15), 3376-3379.
5. Gao, F.; Deng, M.; Qian, C., The effect of coordination on the reaction of N-tosyl imines with diethylzinc. *Tetrahedron* **2005,** *61* (52), 12238-12243.
6. Zhu, M.; Fujita, K.-i.; Yamaguchi, R., Simple and Versatile Catalytic System for N-Alkylation of Sulfonamides with Various Alcohols. *Organic letters* **2010,** *12* (6), 1336-1339.
7. Wallach, D. R.; Chisholm, J. D., Alkylation of Sulfonamides with Trichloroacetimidates under Thermal Conditions. *The Journal of organic chemistry* **2016,** *81* (17), 8035-42.
8. Cui, X.; Shi, F.; Tse, M. K.; GÃ¶rdes, D.; Thurow, K.; Beller, M.; Deng, Y., Copper-CatalyzedN-Alkylation of Sulfonamides with Benzylic Alcohols: Catalysis and Mechanistic Studies. *Advanced Synthesis & Catalysis* **2009,** *351* (17), 2949-2958.
9. Yrjölä, S.; Parkkari, T.; Navia-Paldanius, D.; Laitinen, T.; Kaczor, A. A.; Kokkola, T.; Adusei-Mensah, F.; Savinainen, J. R.; Laitinen, J. T.; Poso, A.; Alexander, A.; Penman, J.; Stott, L.; Anskat, M.; Irving, A. J.; Nevalainen, T. J., Potent and selective N-(4-sulfamoylphenyl)thiourea-based GPR55 agonists. *European Journal of Medicinal Chemistry* **2016,** *107*, 119-132.
10. Caddick, S.; Wilden, J. D.; Judd, D. B., Direct Synthesis of Sulfonamides and Activated Sulfonate Esters from Sulfonic Acids. *Journal of the American Chemical Society* **2004,** *126* (4), 1024-1025.
11. Yu, X.; Liu, C.; Jiang, L.; Xu, Q., Manganese Dioxide Catalyzed N-Alkylation of Sulfonamides and Amines with Alcohols under Air. *Organic letters* **2011,** *13* (23), 6184-6187.
12. Li, Z.-l.; Jin, L.-k.; Cai, C., Nickel-catalyzed product-controllable amidation and imidation of sp3 C-H bonds in substituted toluenes with sulfonamides. *Organic & biomolecular chemistry* **2017,** *15* (6), 1317-1320.

This report was created by ACD/NMR Processor Academic Edition. For more information go to www.acdlabs.com/nmrproc/

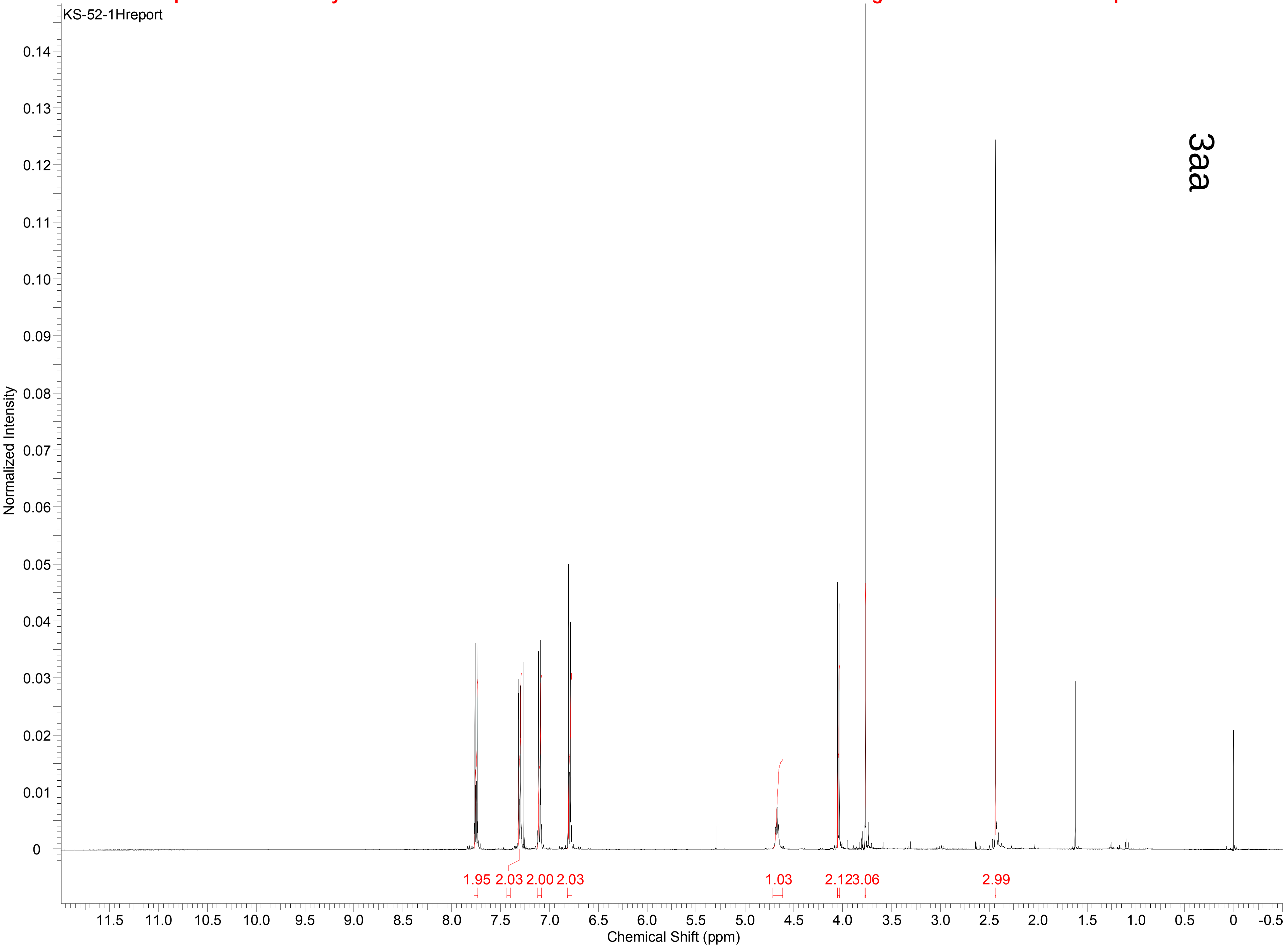

3aa

This report was created by ACD/NMR Processor Academic Edition. For more information go to www.acdlabs.com/nmrproc/

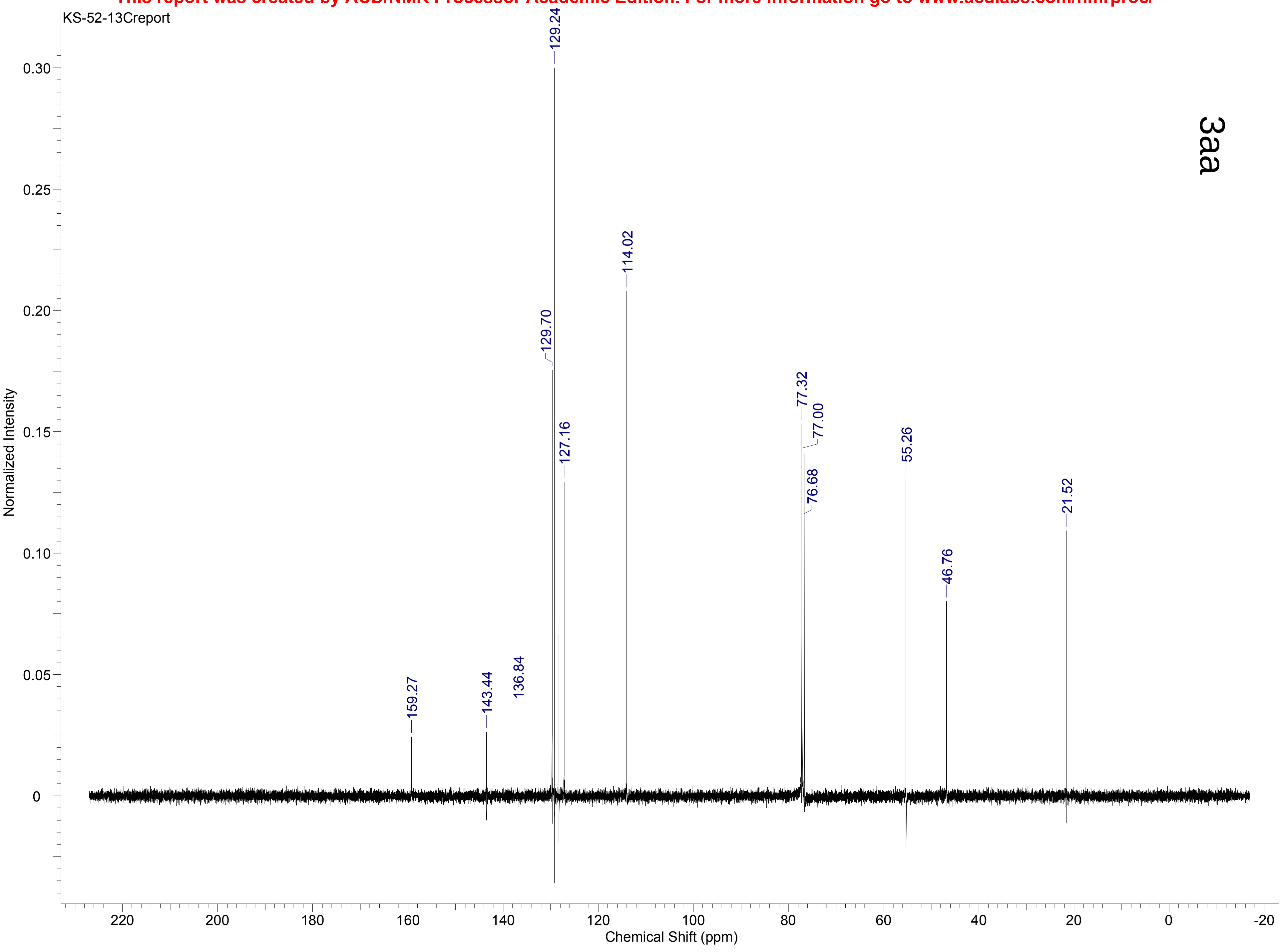

This report was created by ACD/NMR Processor Academic Edition. For more information go to www.acdlabs.com/nmrproc/

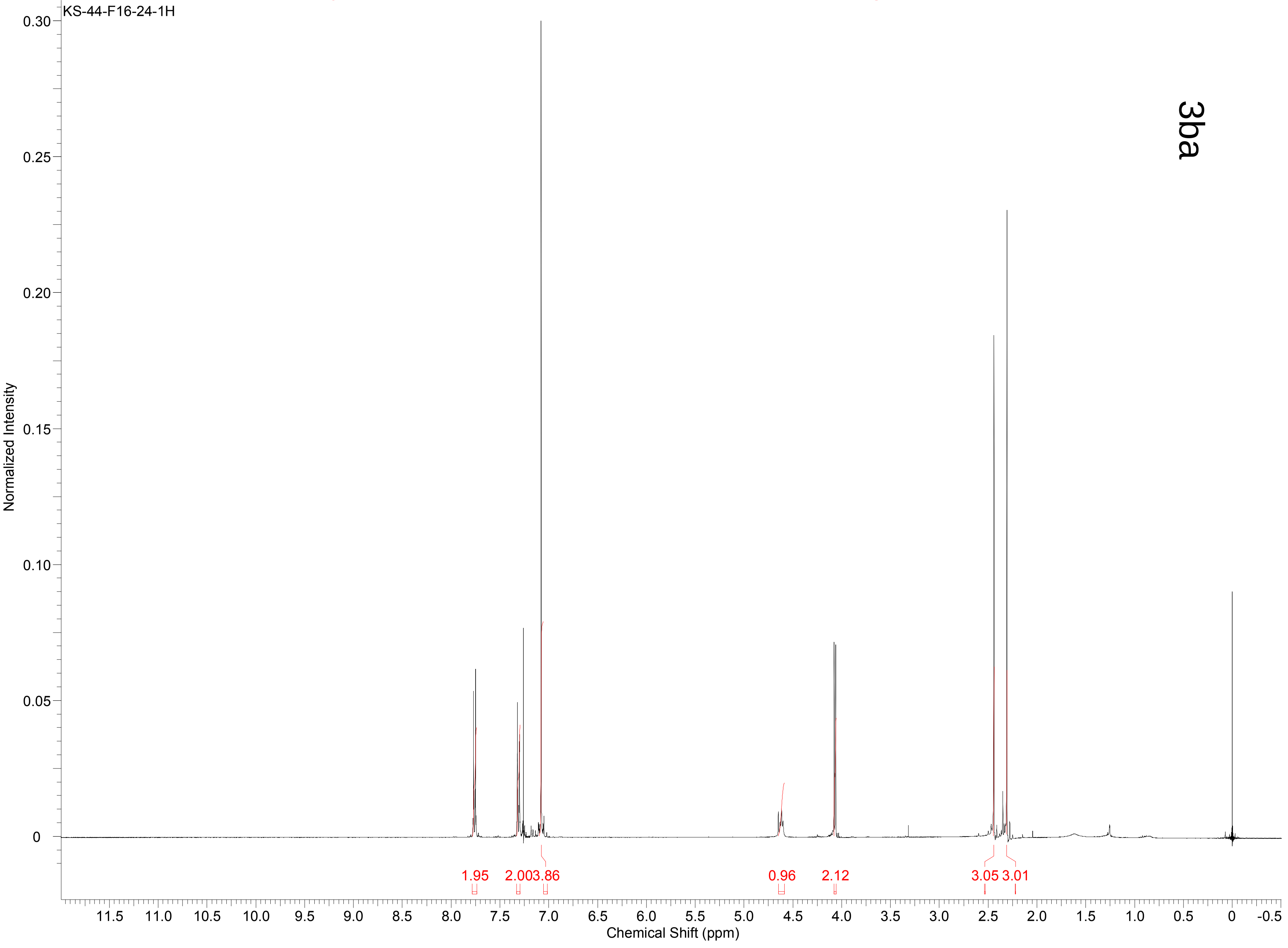

This report was created by ACD/NMR Processor Academic Edition. For more information go to www.acdlabs.com/nmrproc/

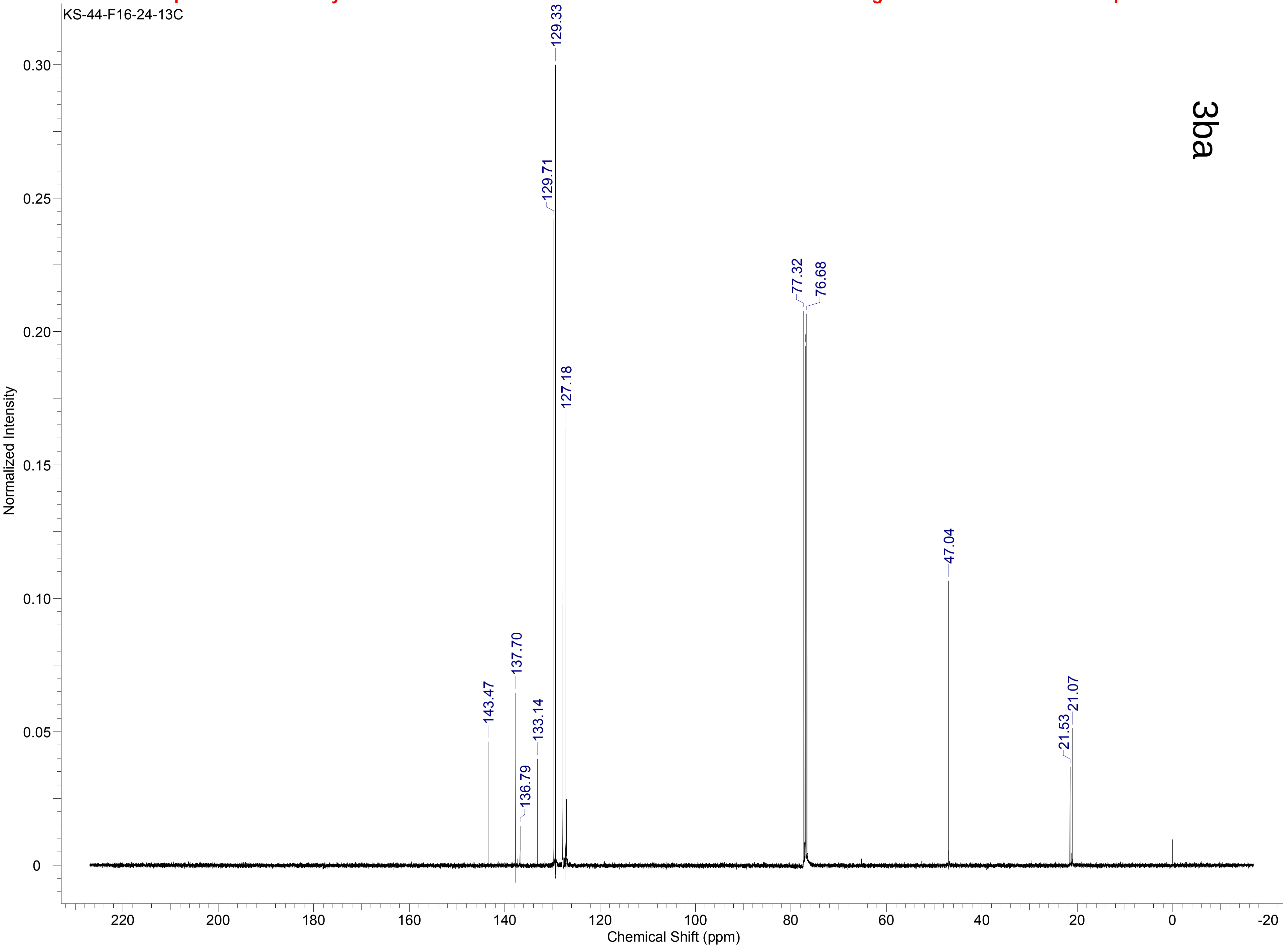

This report was created by ACD/NMR Processor Academic Edition. For more information go to www.acdlabs.com/nmrproc/

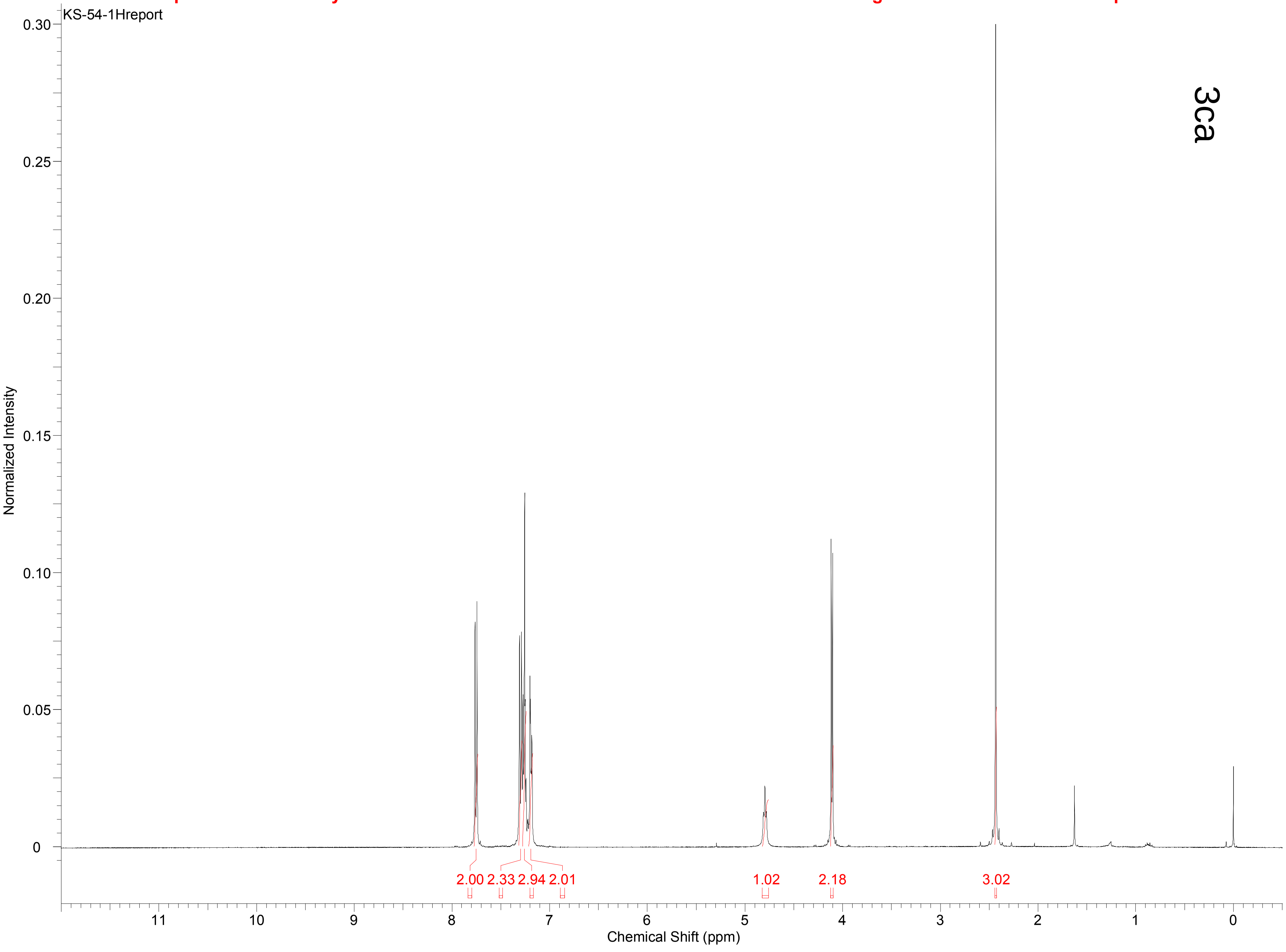

This report was created by ACD/NMR Processor Academic Edition. For more information go to www.acdlabs.com/nmrproc/

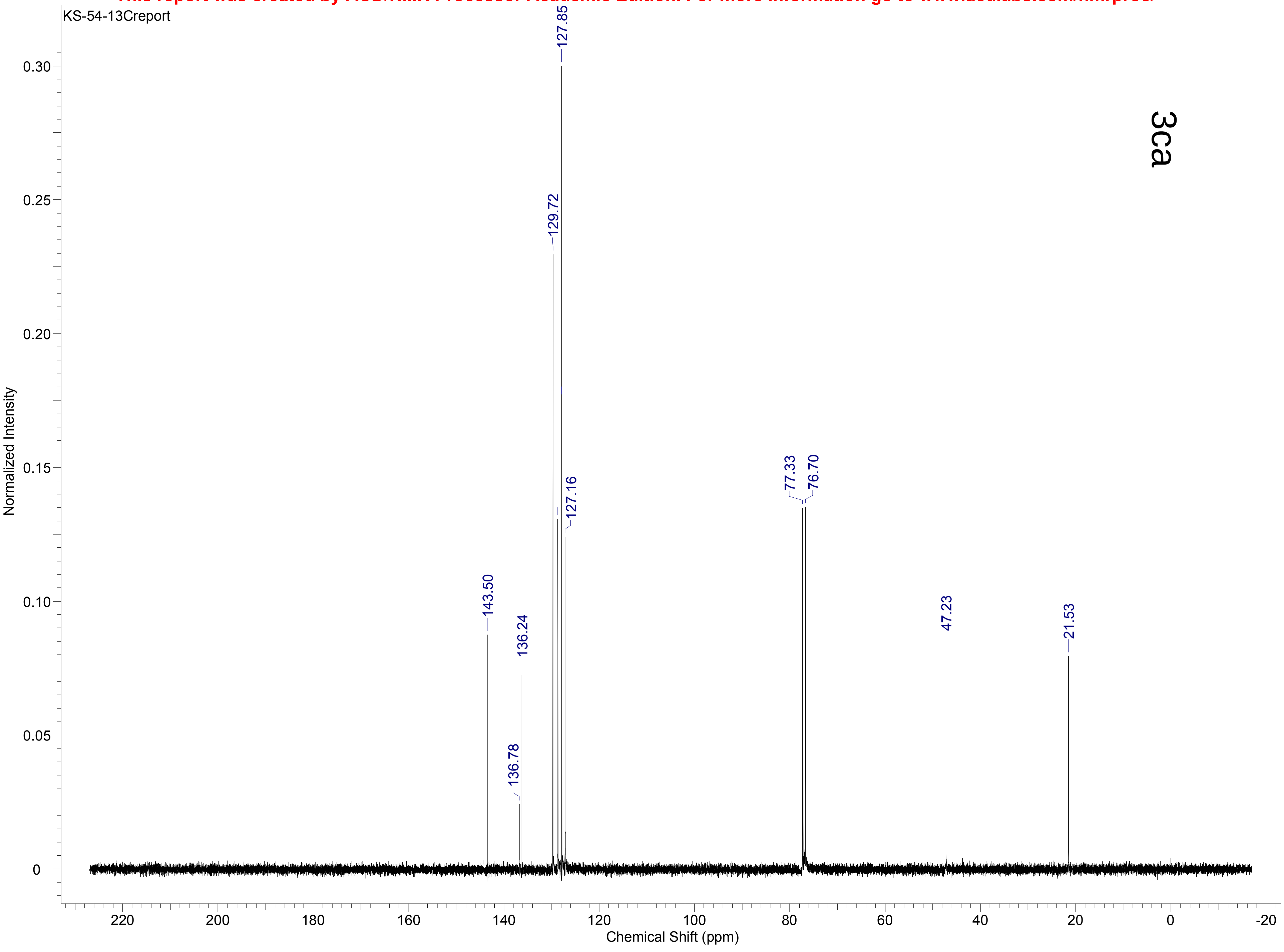

This report was created by ACD/NMR Processor Academic Edition. For more information go to www.acdlabs.com/nmrproc/

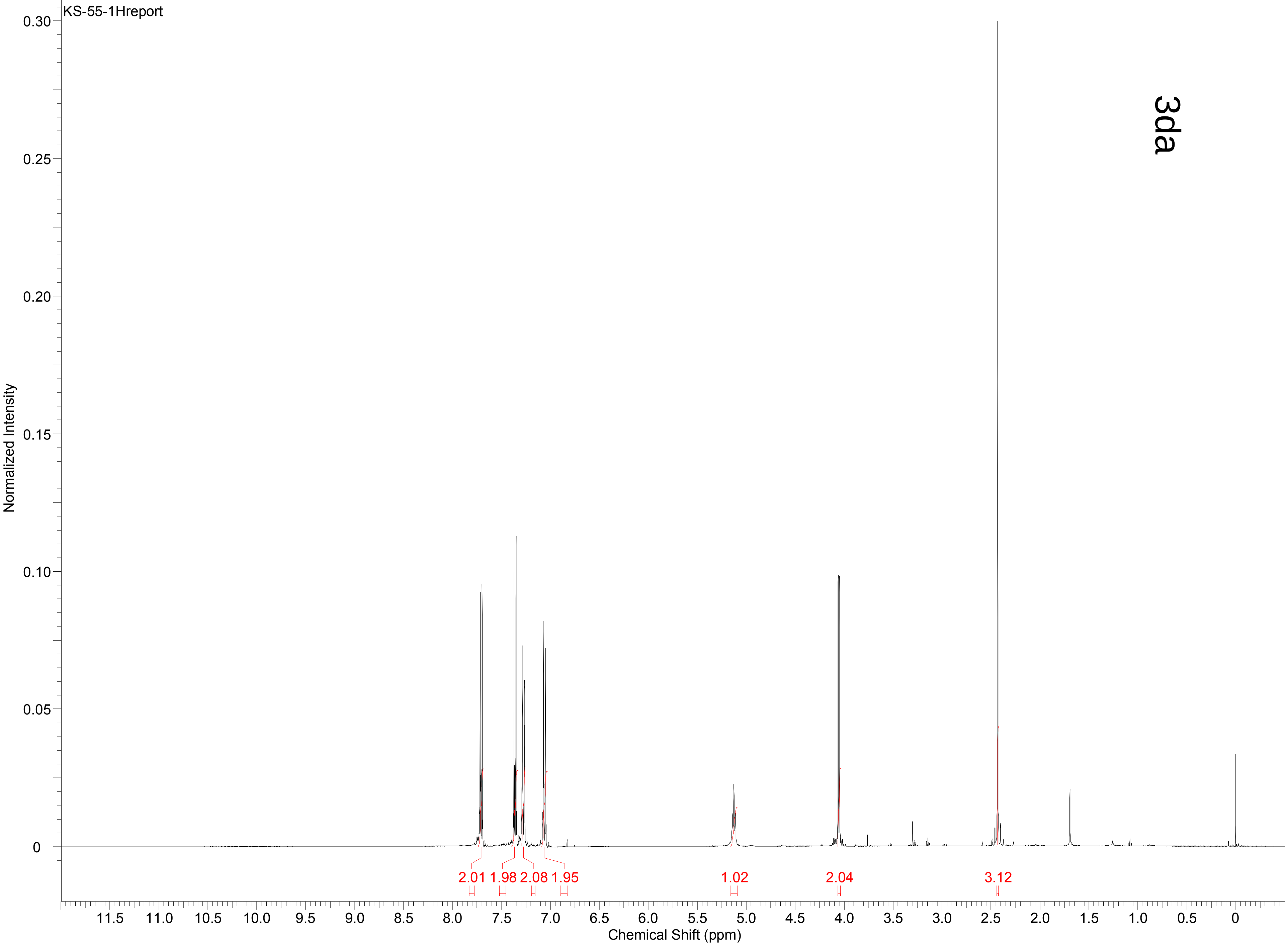

This report was created by ACD/NMR Processor Academic Edition. For more information go to www.acdlabs.com/nmrproc/

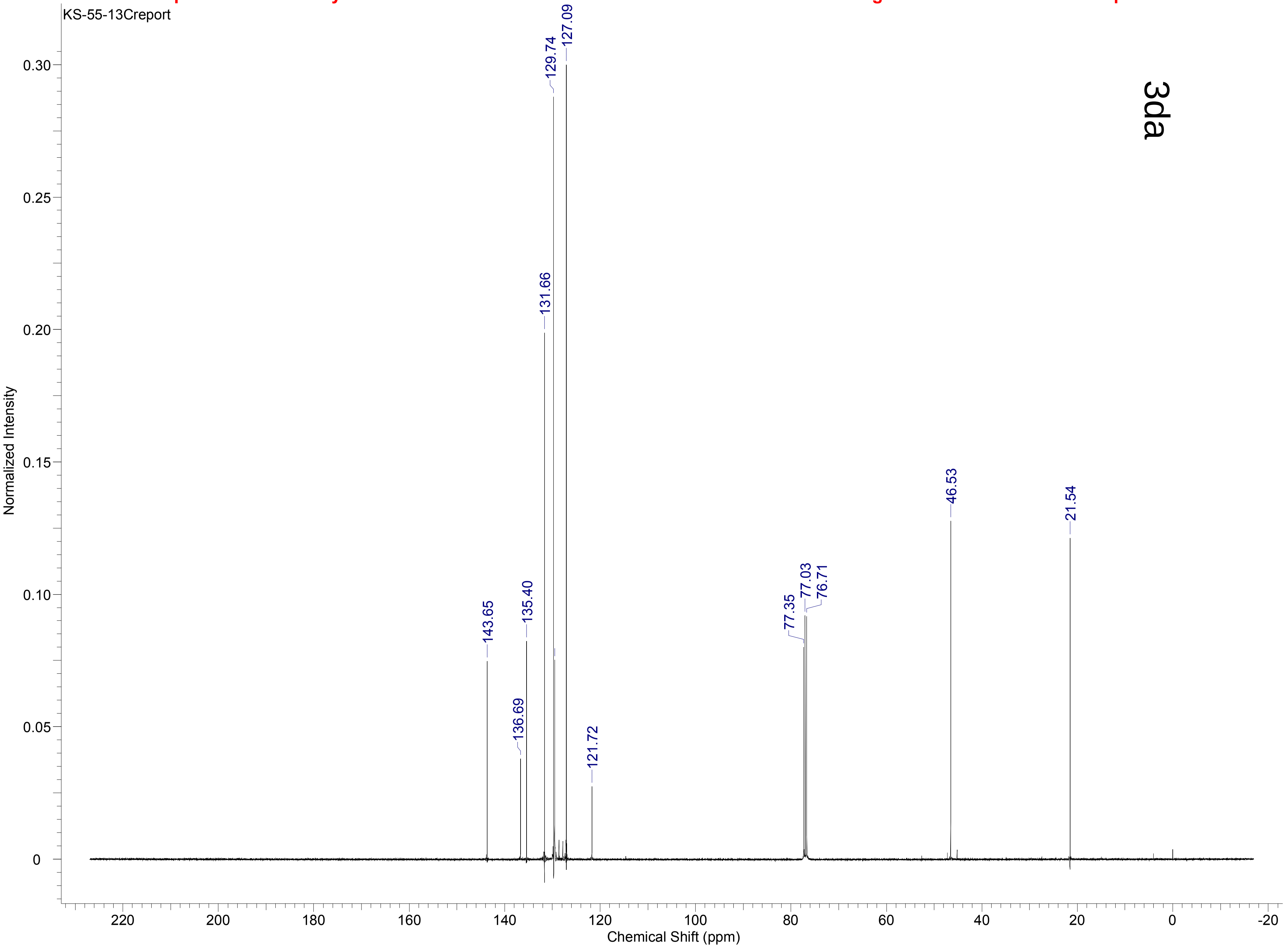

This report was created by ACD/NMR Processor Academic Edition. For more information go to www.acdlabs.com/nmrproc/

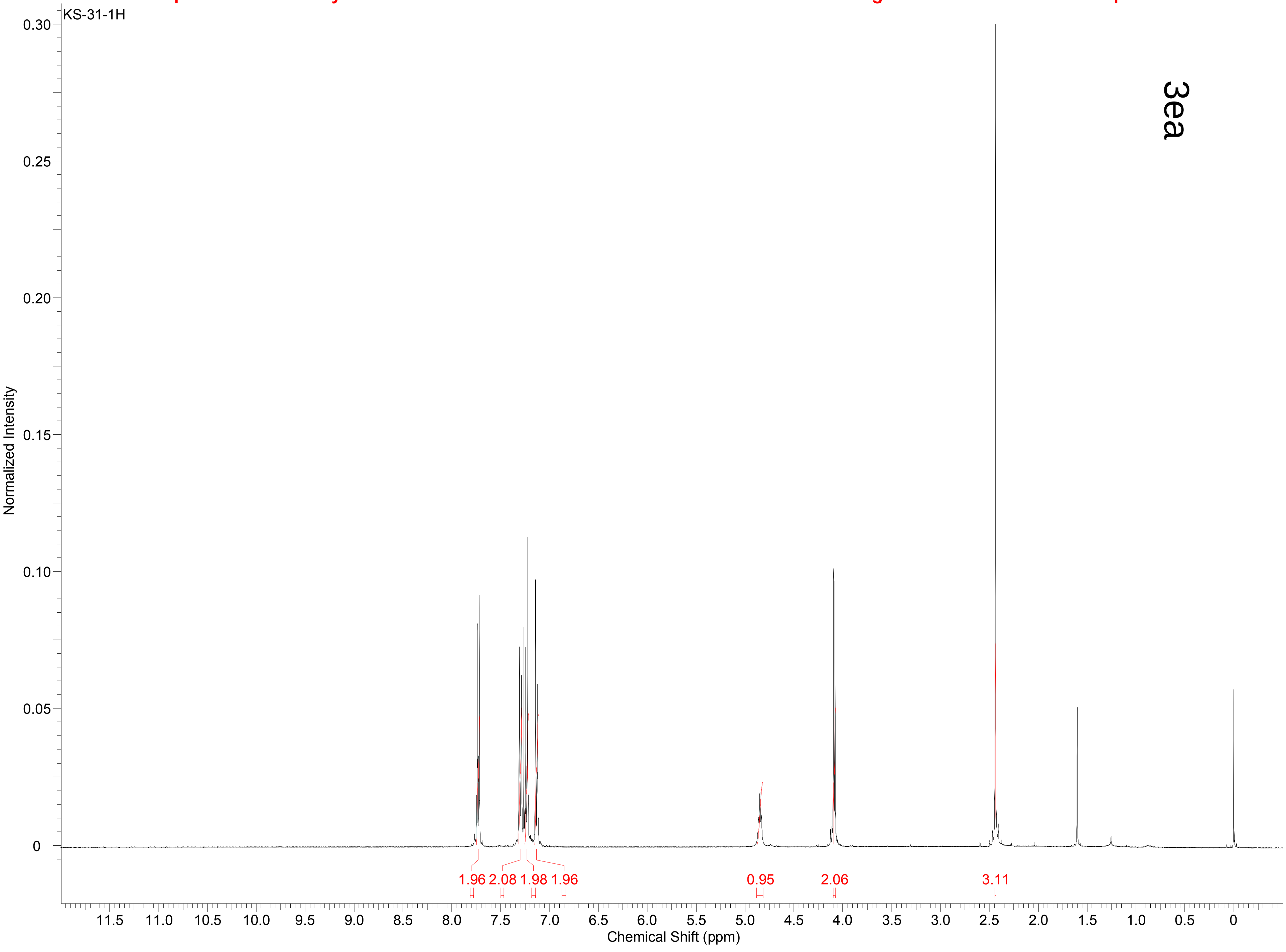

This report was created by ACD/NMR Processor Academic Edition. For more information go to www.acdlabs.com/nmrproc/

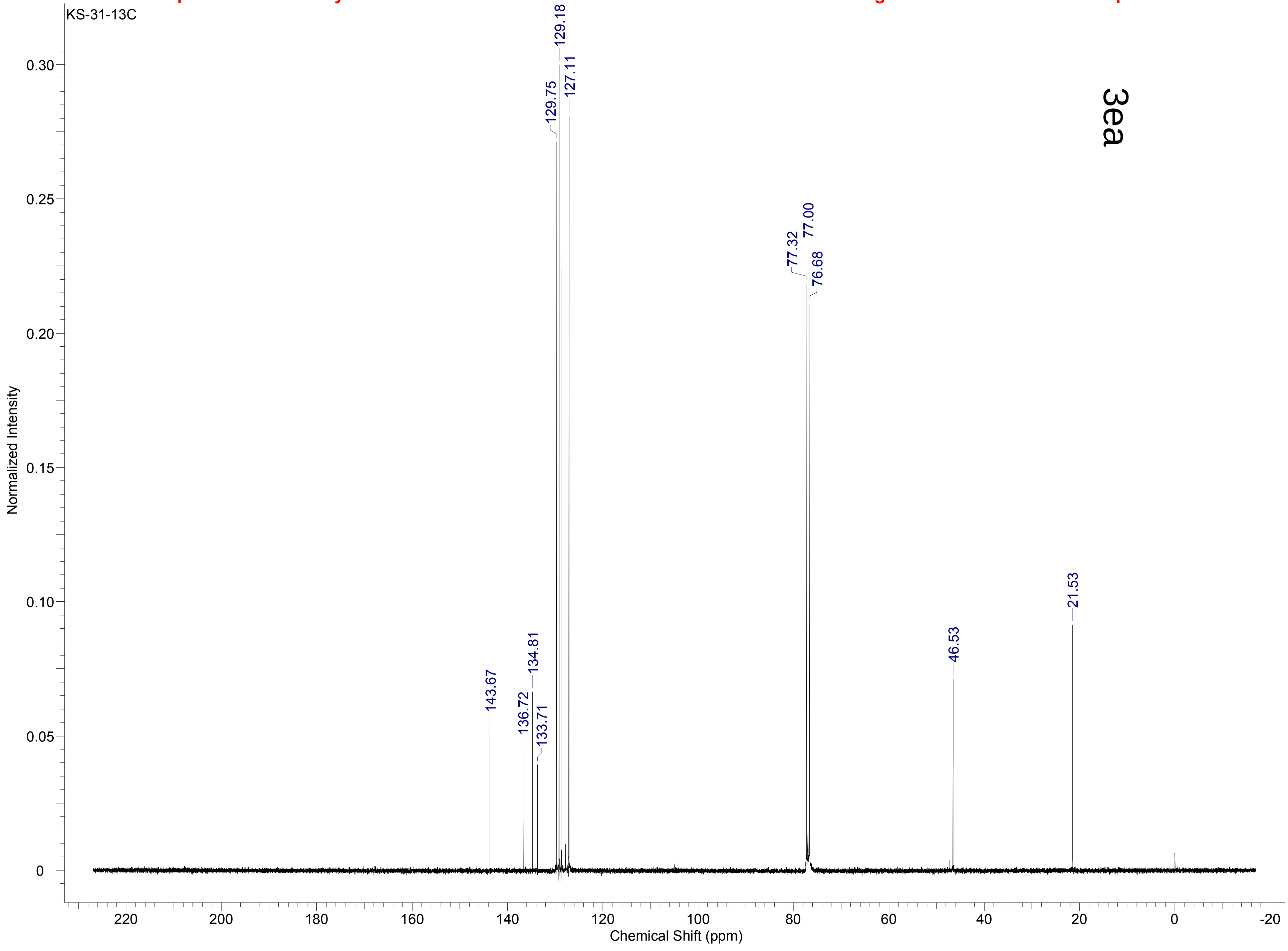

This report was created by ACD/NMR Processor Academic Edition. For more information go to www.acdlabs.com/nmrproc/

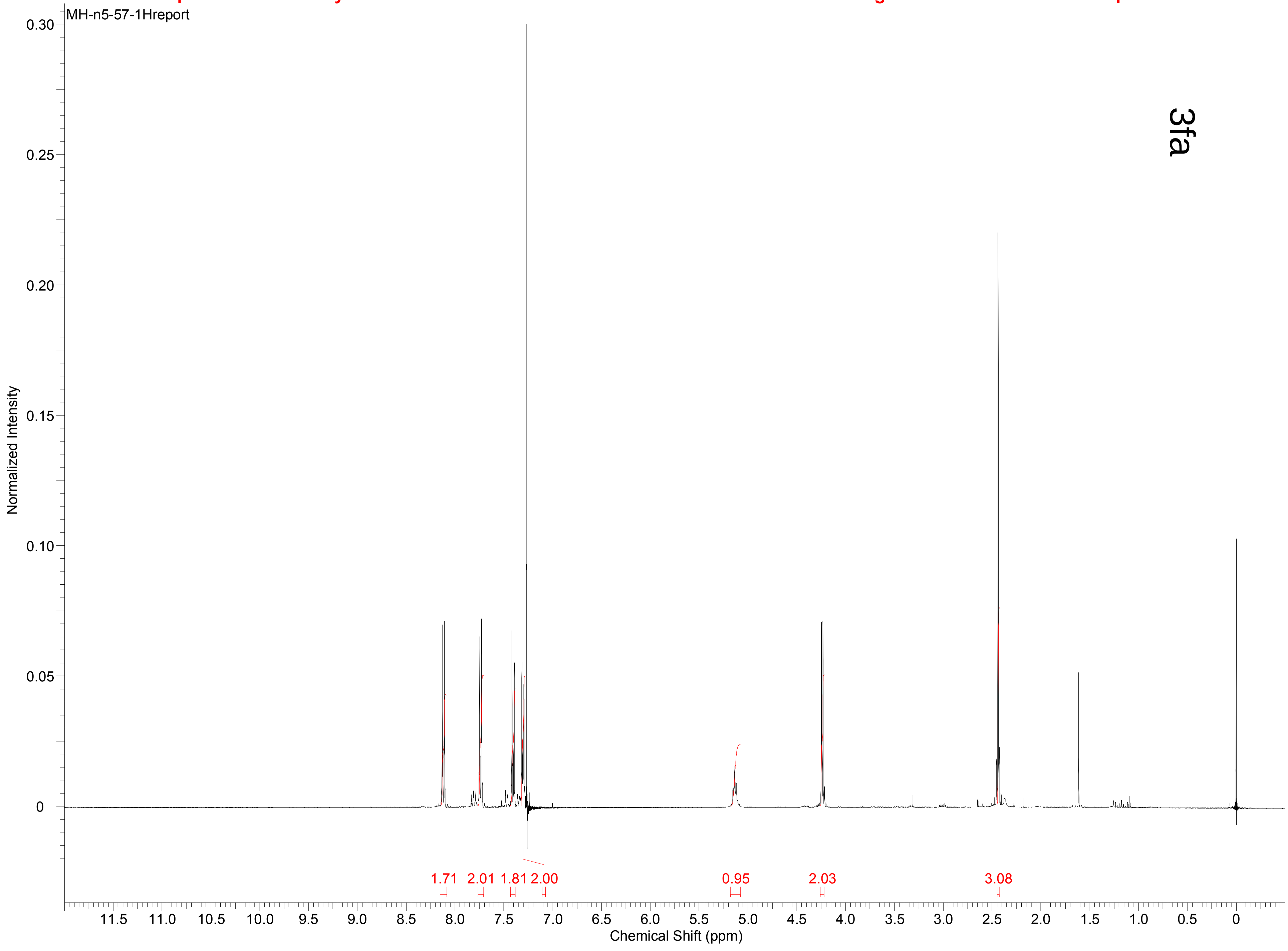

This report was created by ACD/NMR Processor Academic Edition. For more information go to www.acdlabs.com/nmrproc/

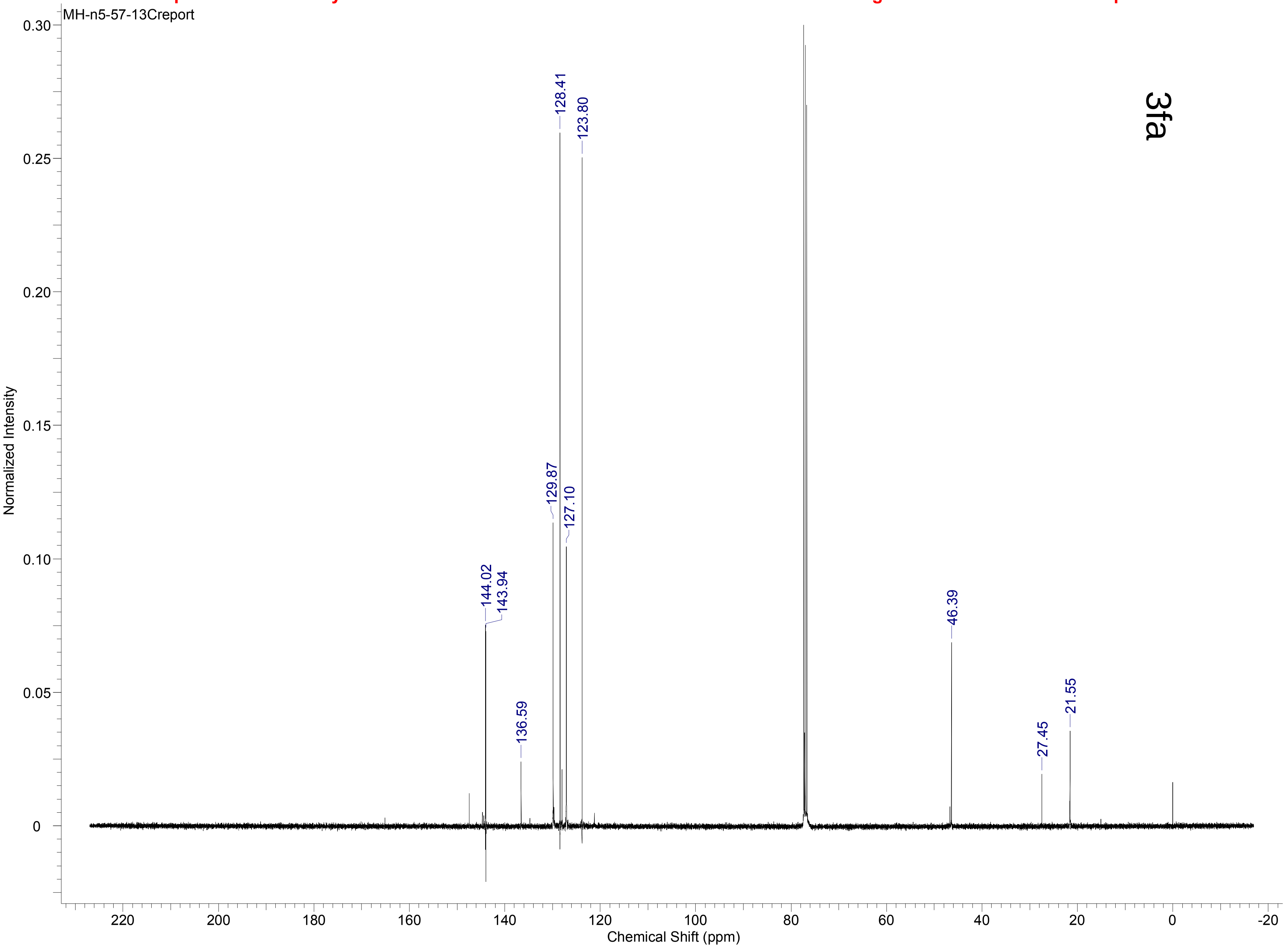

This report was created by ACD/NMR Processor Academic Edition. For more information go to www.acdlabs.com/nmrproc/

3ga

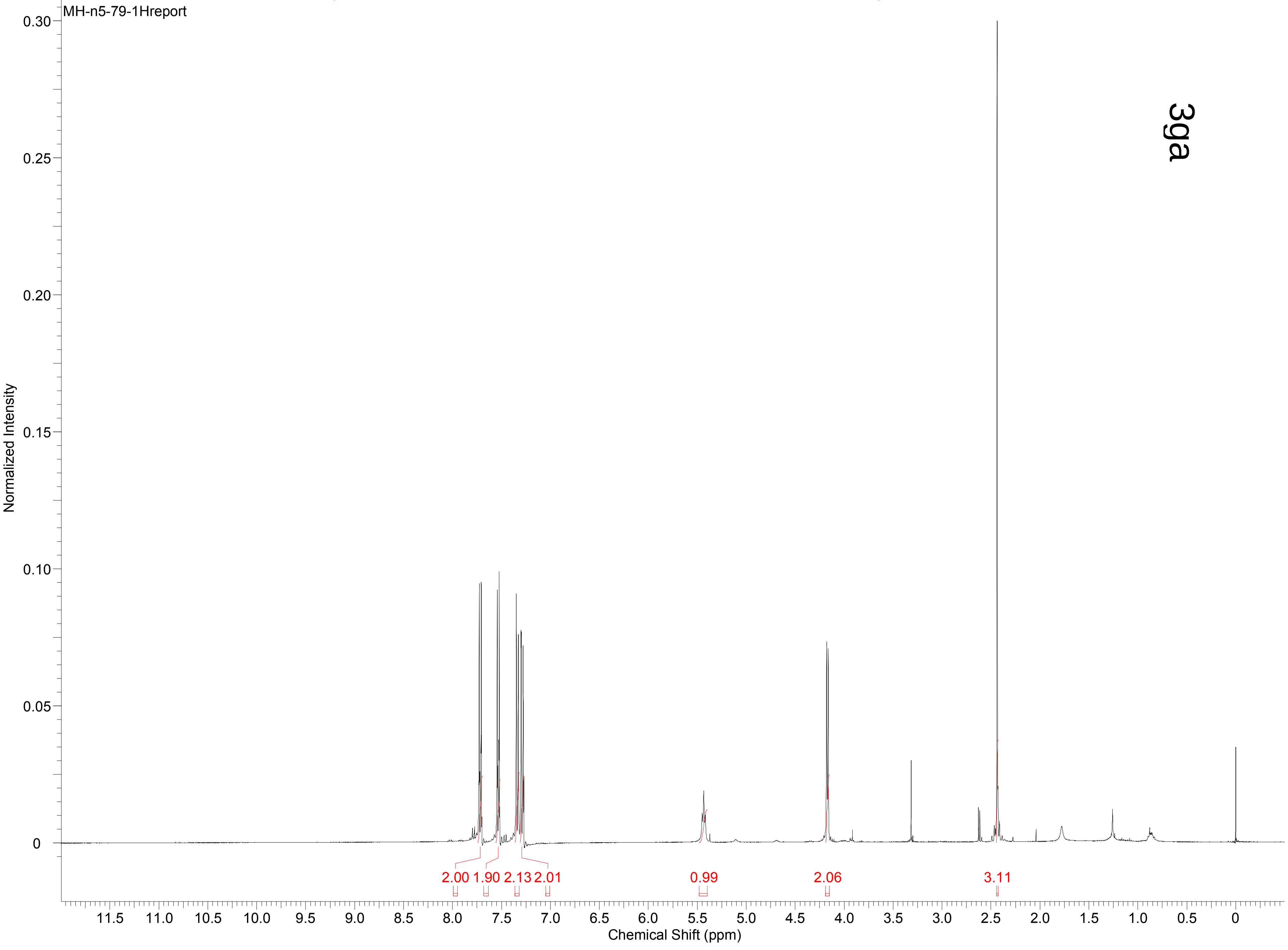

This report was created by ACD/NMR Processor Academic Edition. For more information go to www.acdlabs.com/nmrproc/

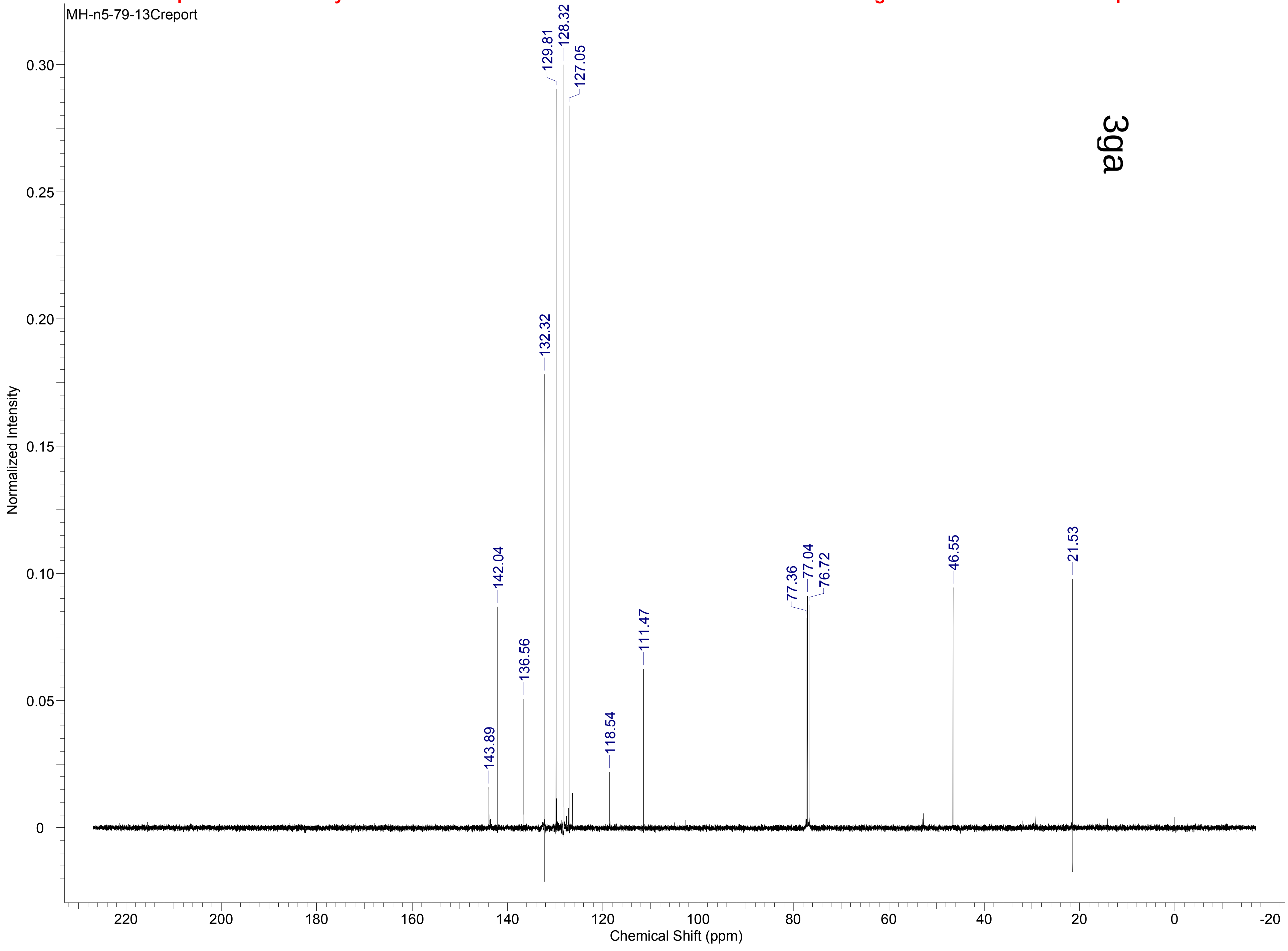

This report was created by ACD/NMR Processor Academic Edition. For more information go to www.acdlabs.com/nmrproc/

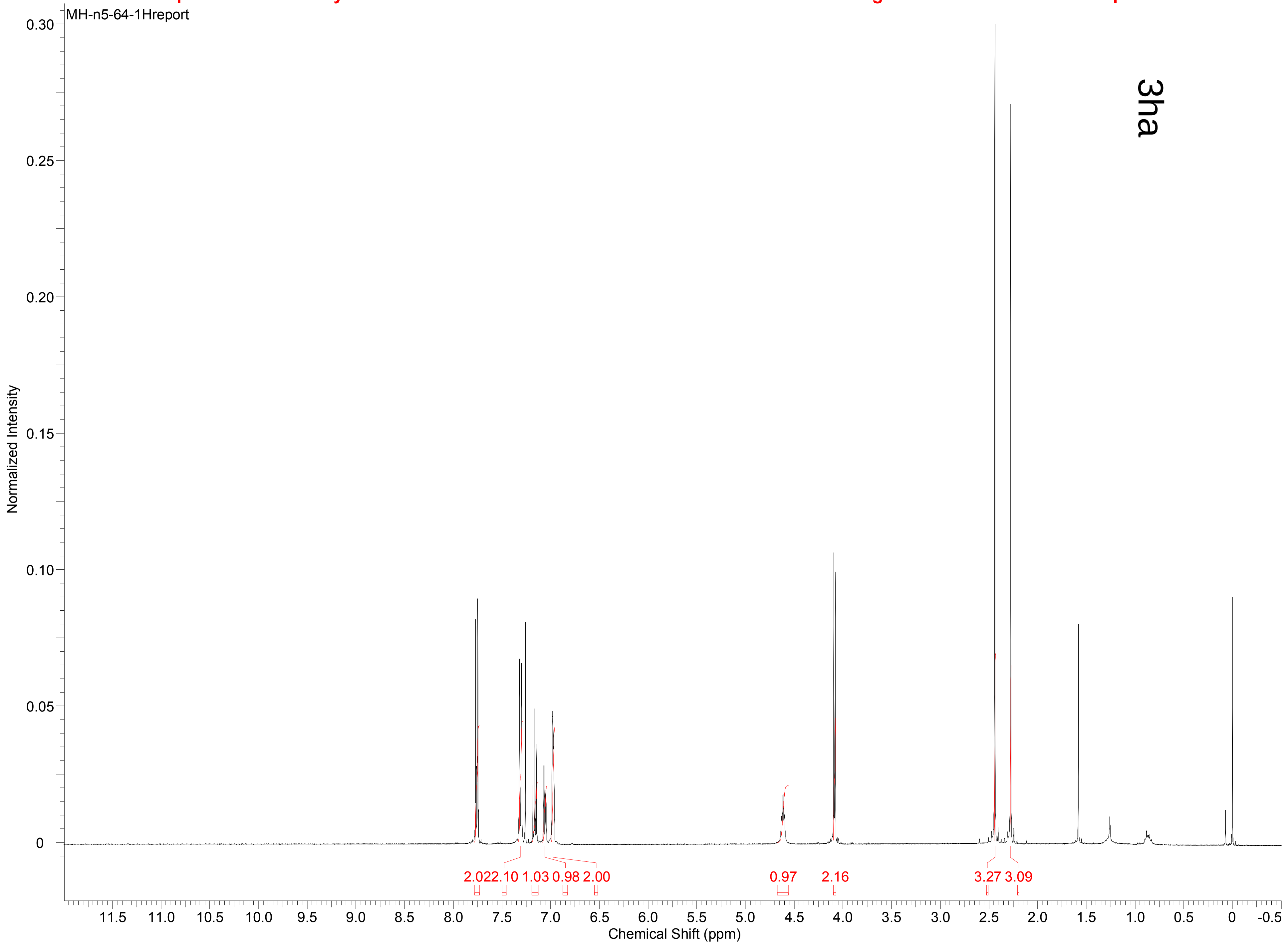

This report was created by ACD/NMR Processor Academic Edition. For more information go to www.acdlabs.com/nmrproc/

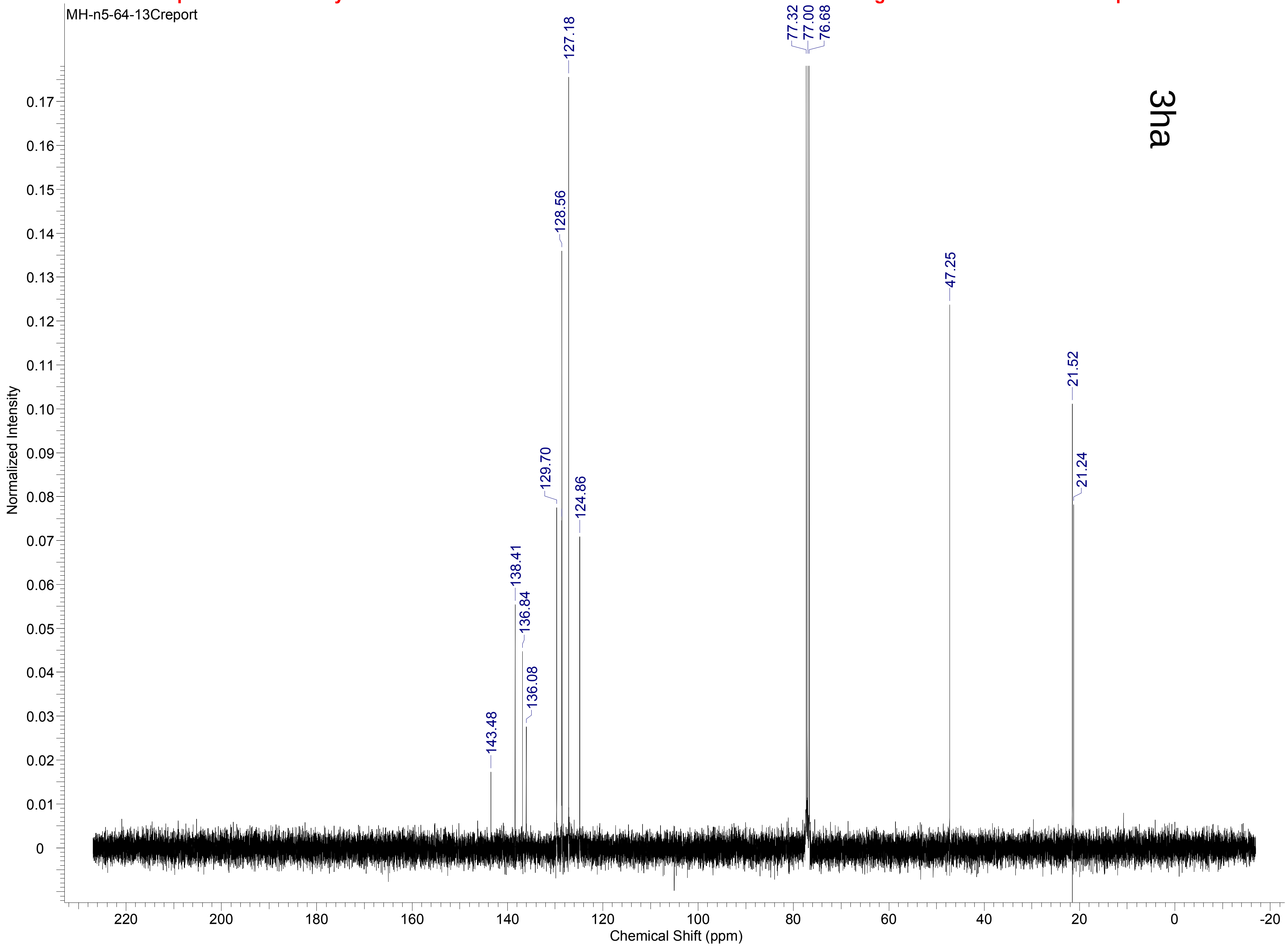

This report was created by ACD/NMR Processor Academic Edition. For more information go to www.acdlabs.com/nmrproc/

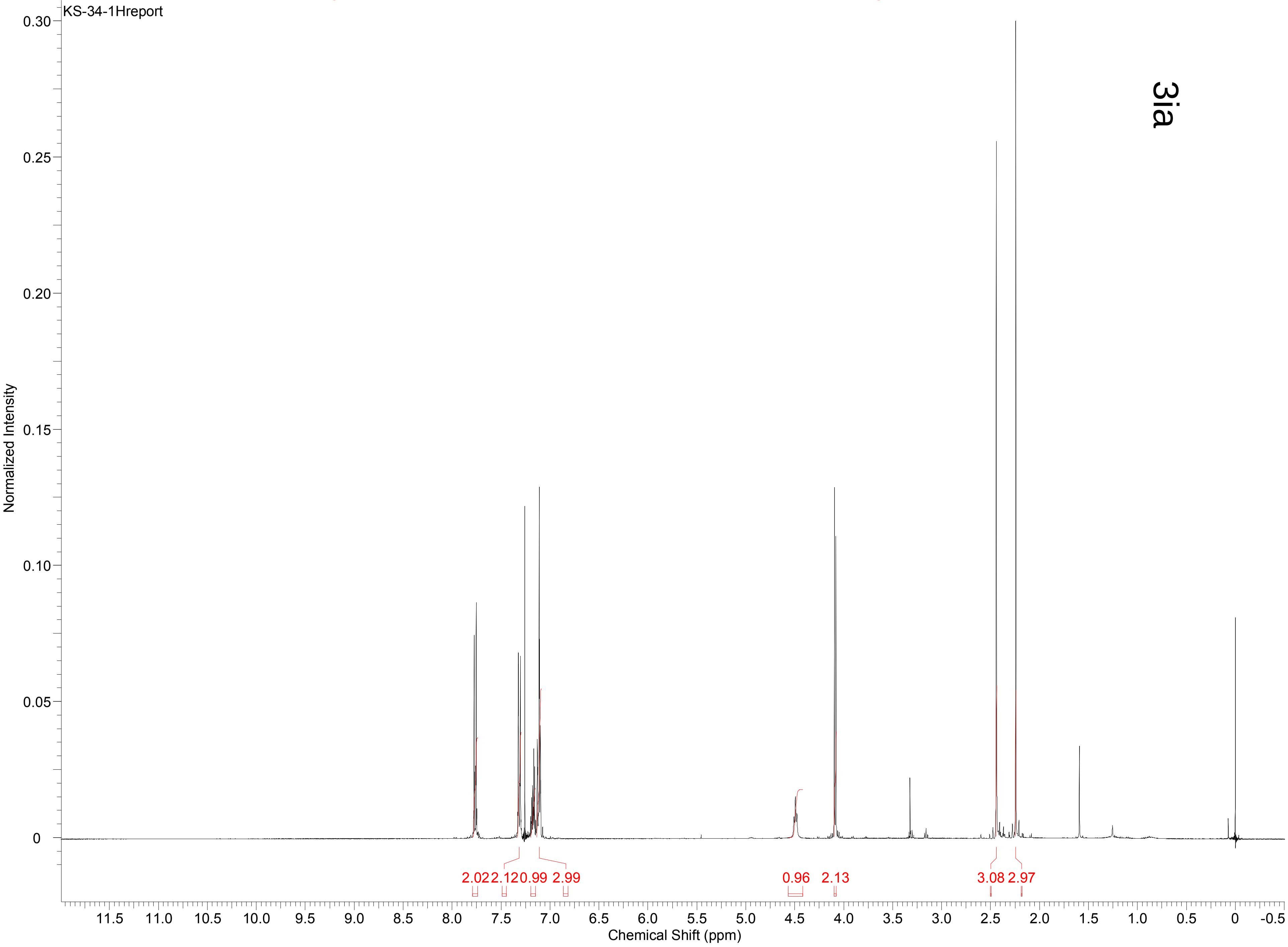

This report was created by ACD/NMR Processor Academic Edition. For more information go to www.acdlabs.com/nmrproc/

KS-34-13Creport

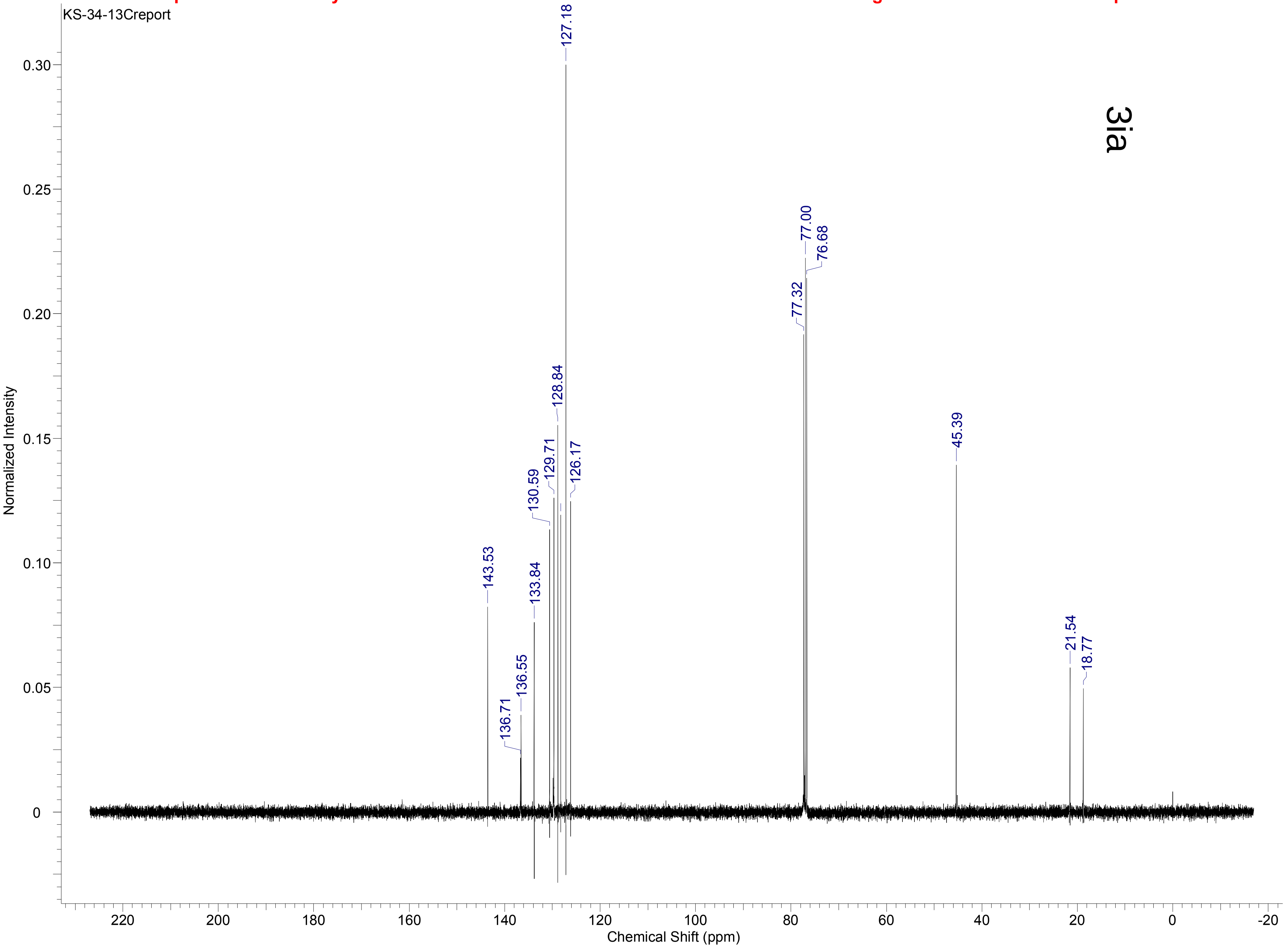

This report was created by ACD/NMR Processor Academic Edition. For more information go to www.acdlabs.com/nmrproc/

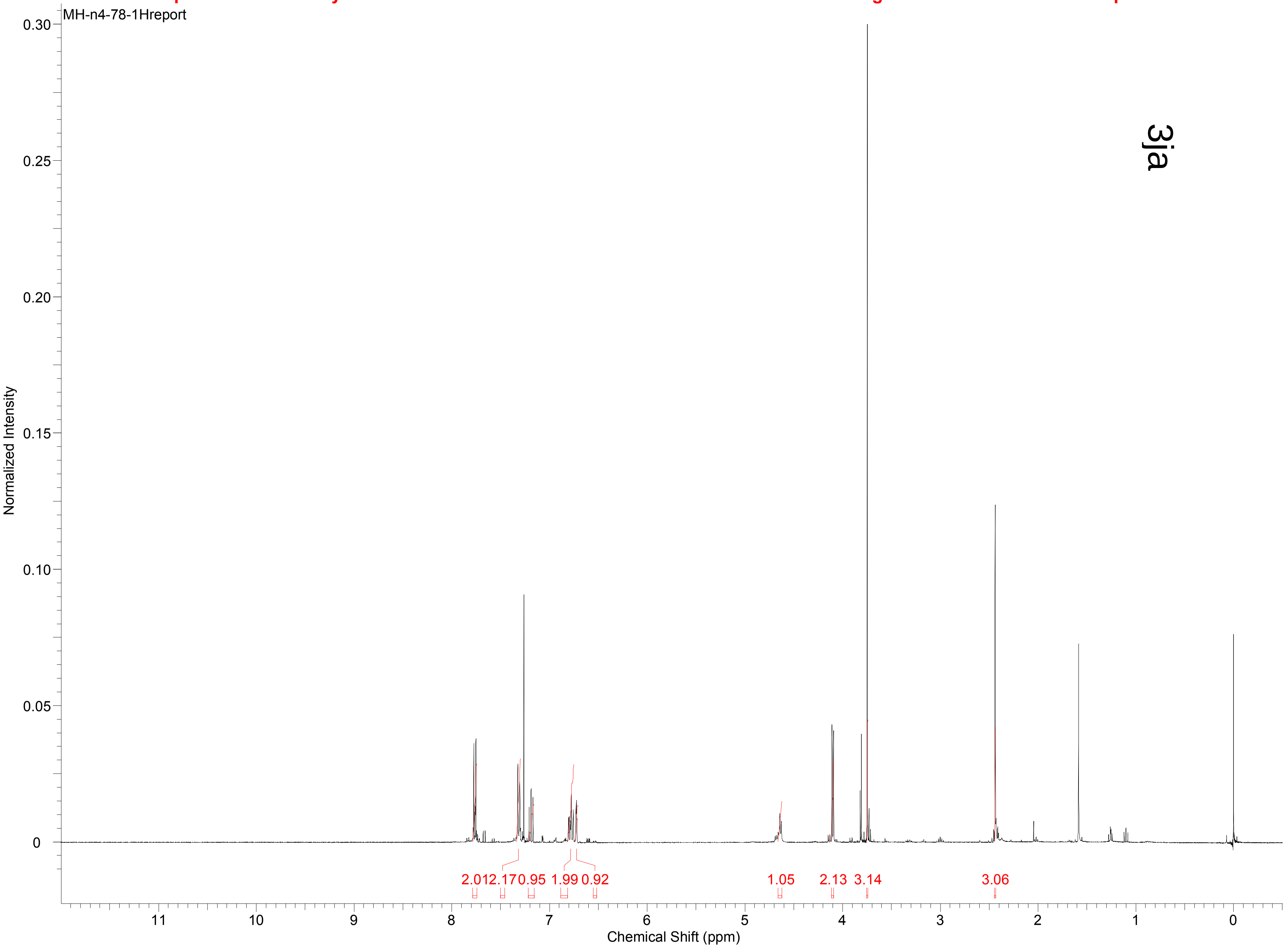

This report was created by ACD/NMR Processor Academic Edition. For more information go to www.acdlabs.com/nmrproc/

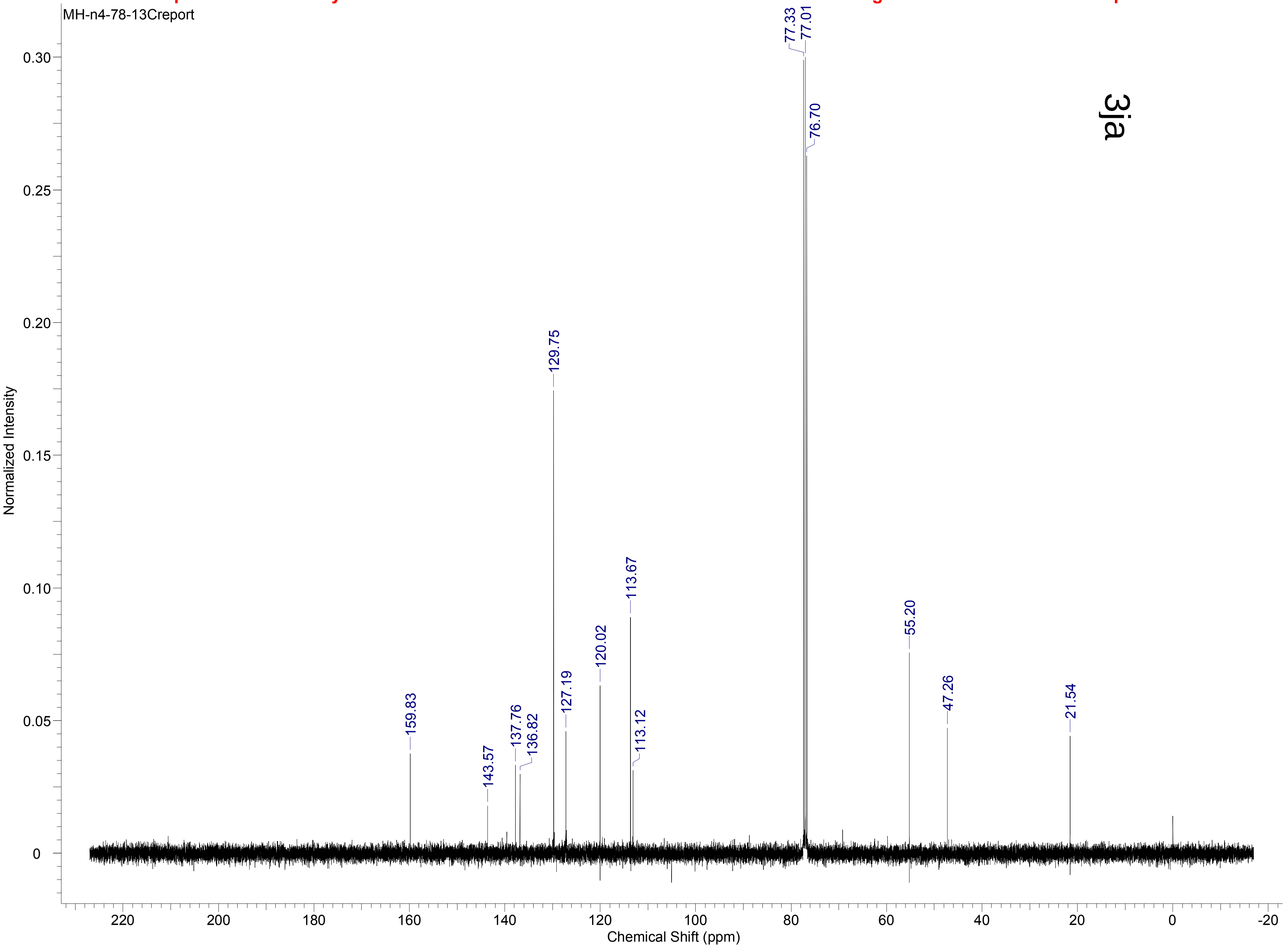

This report was created by ACD/NMR Processor Academic Edition. For more information go to www.acdlabs.com/nmrproc/

3ka

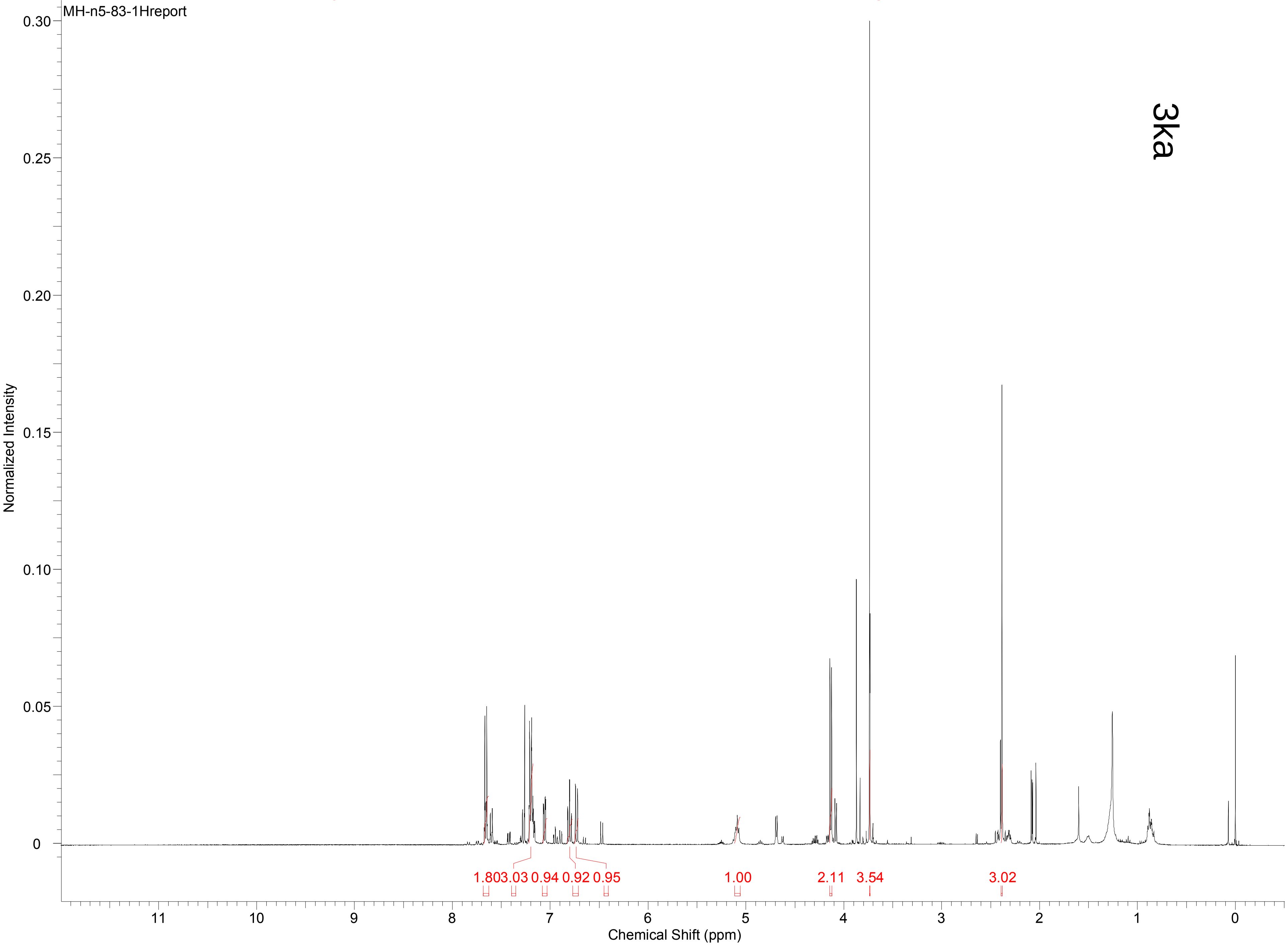

This report was created by ACD/NMR Processor Academic Edition. For more information go to www.acdlabs.com/nmrproc/

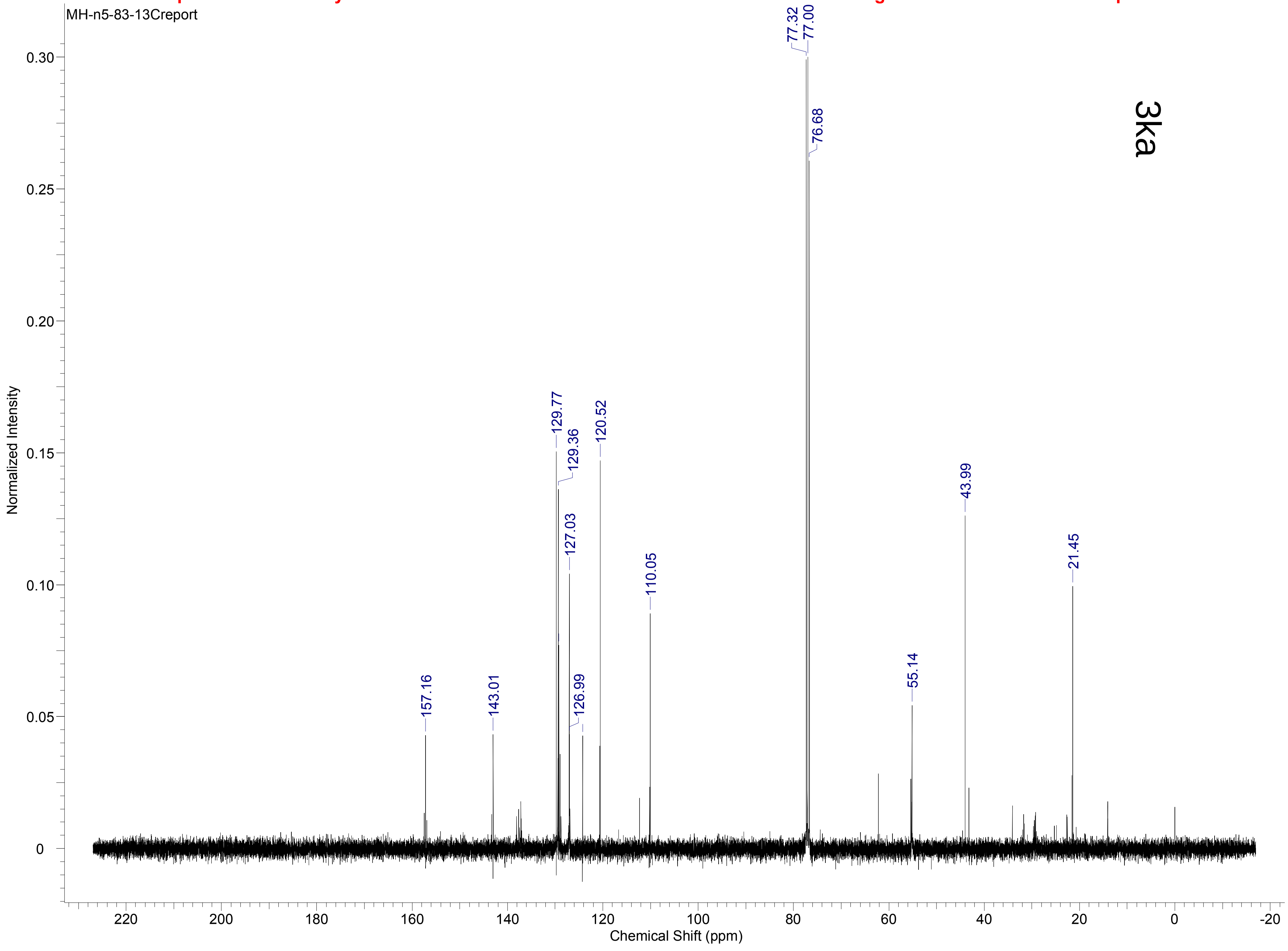

This report was created by ACD/NMR Processor Academic Edition. For more information go to www.acdlabs.com/nmrproc/

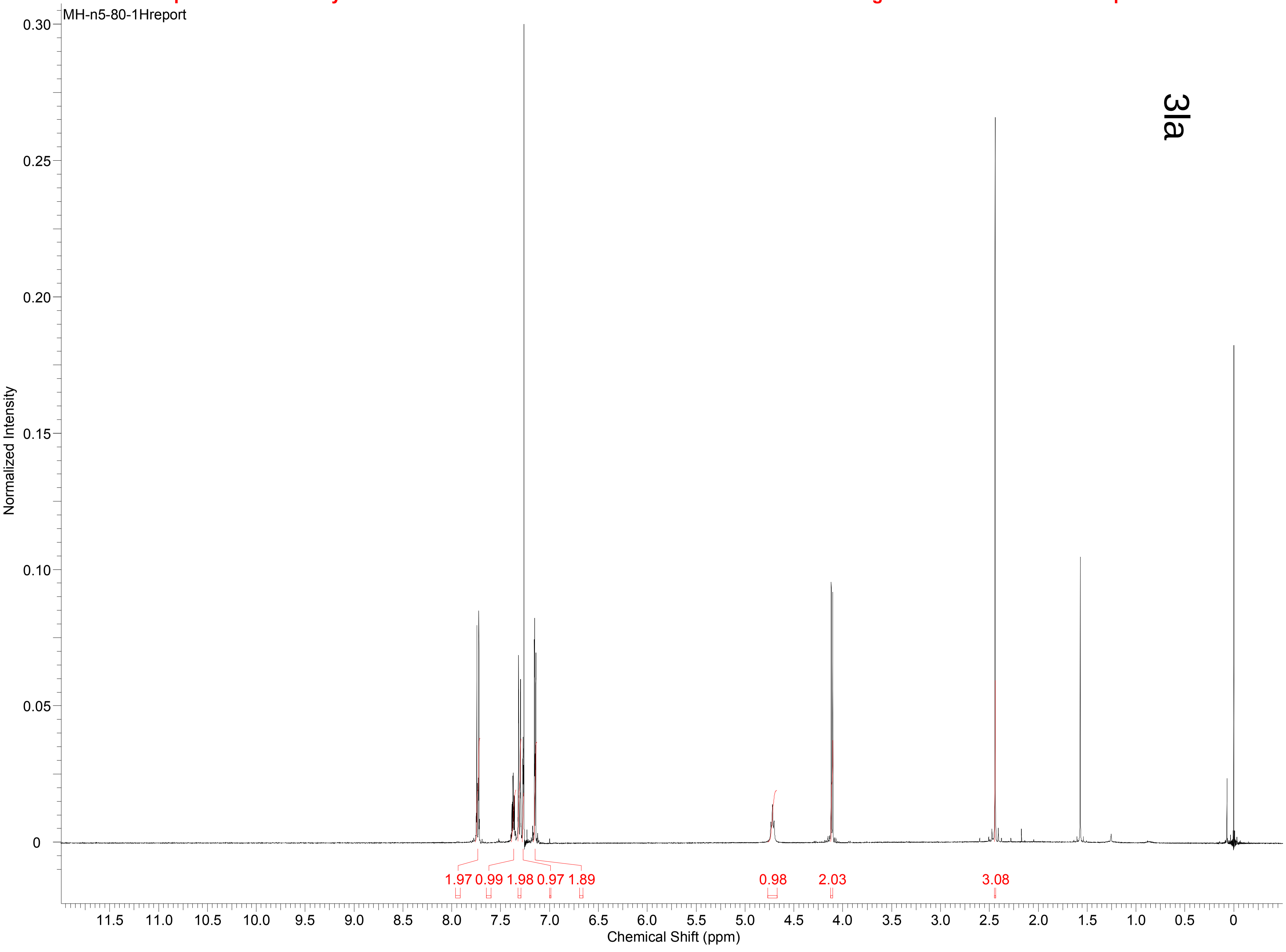

This report was created by ACD/NMR Processor Academic Edition. For more information go to www.acdlabs.com/nmrproc/

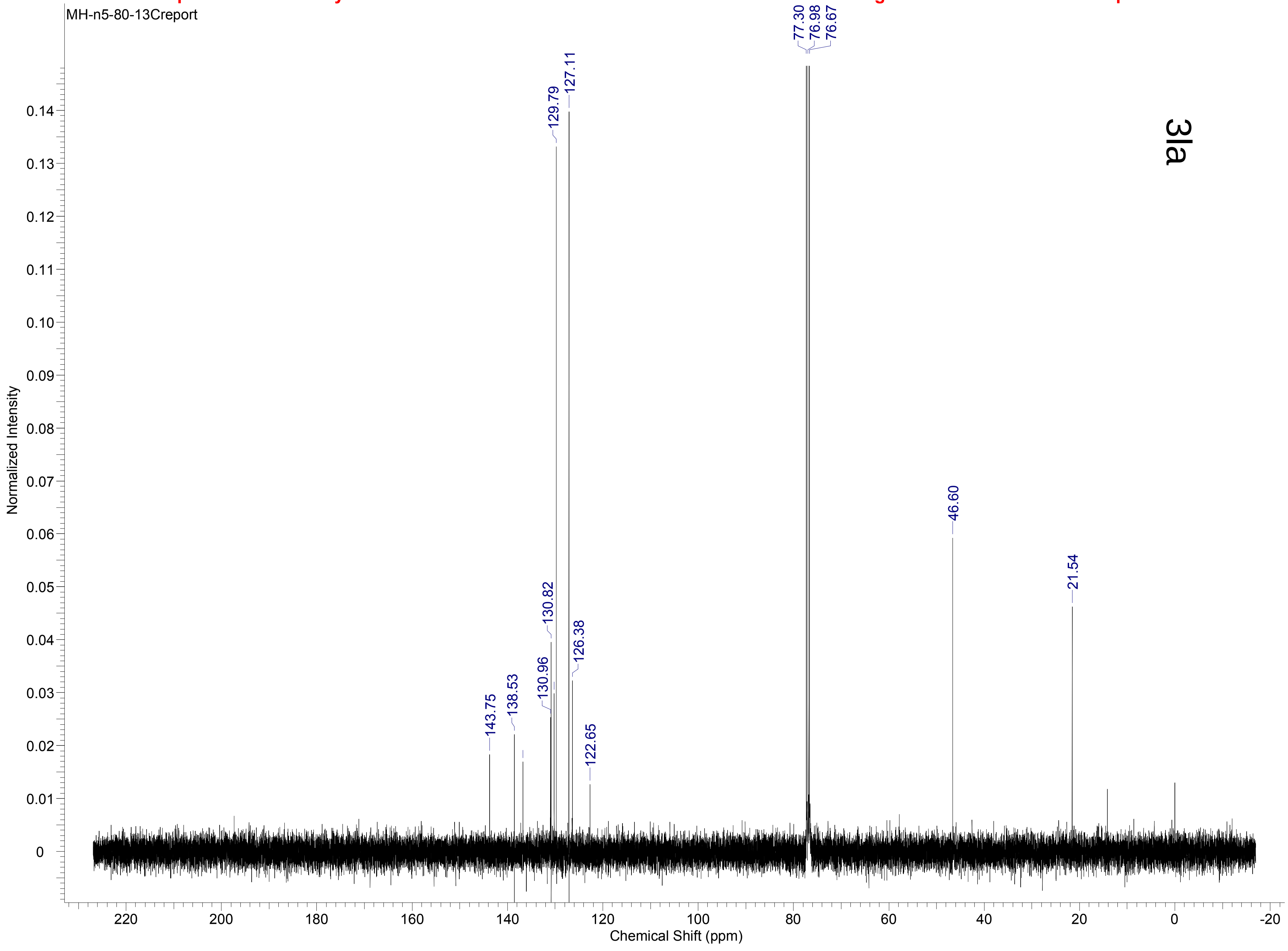

This report was created by ACD/NMR Processor Academic Edition. For more information go to www.acdlabs.com/nmrproc/

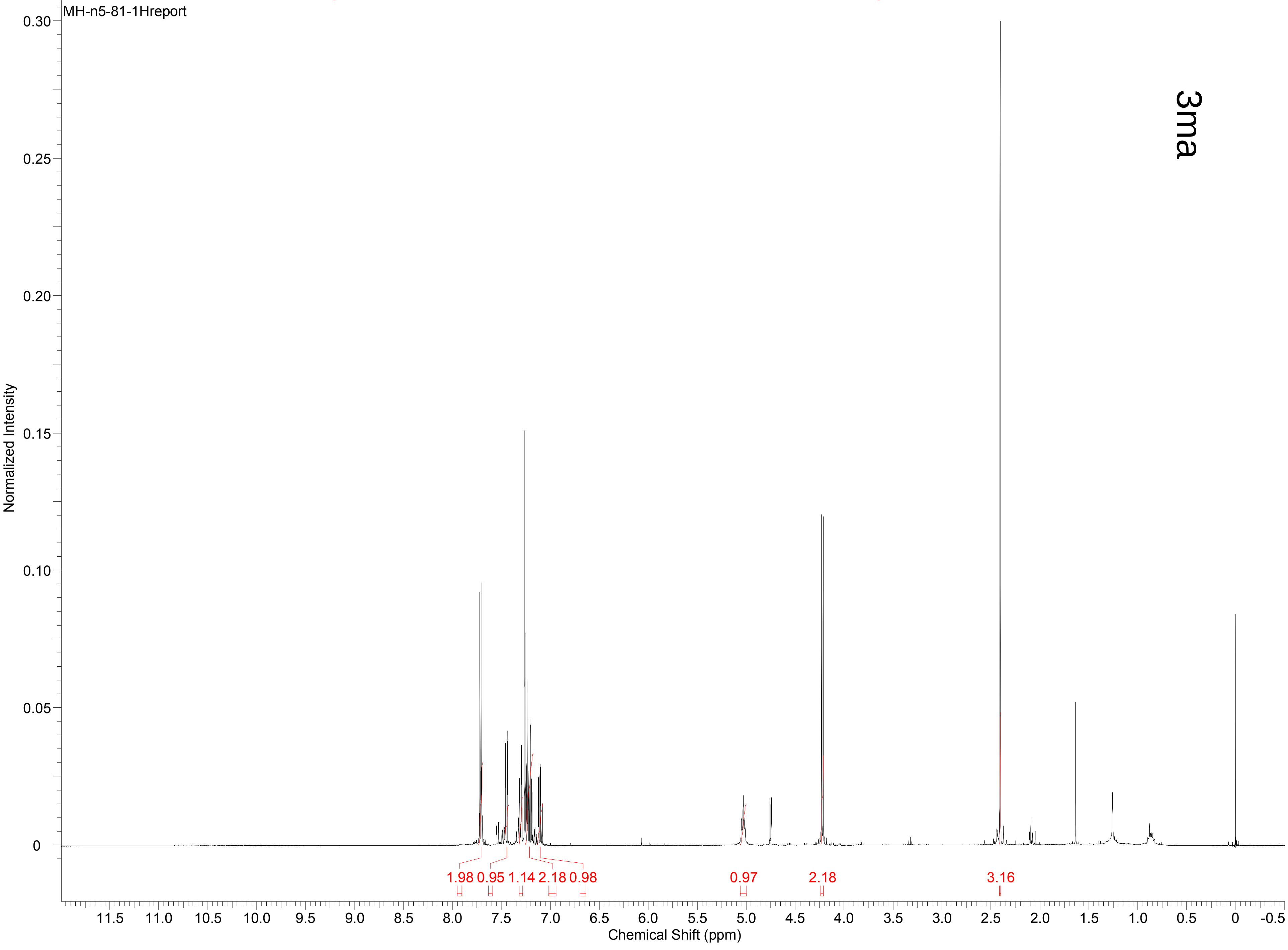

This report was created by ACD/NMR Processor Academic Edition. For more information go to www.acdlabs.com/nmrproc/

3ma

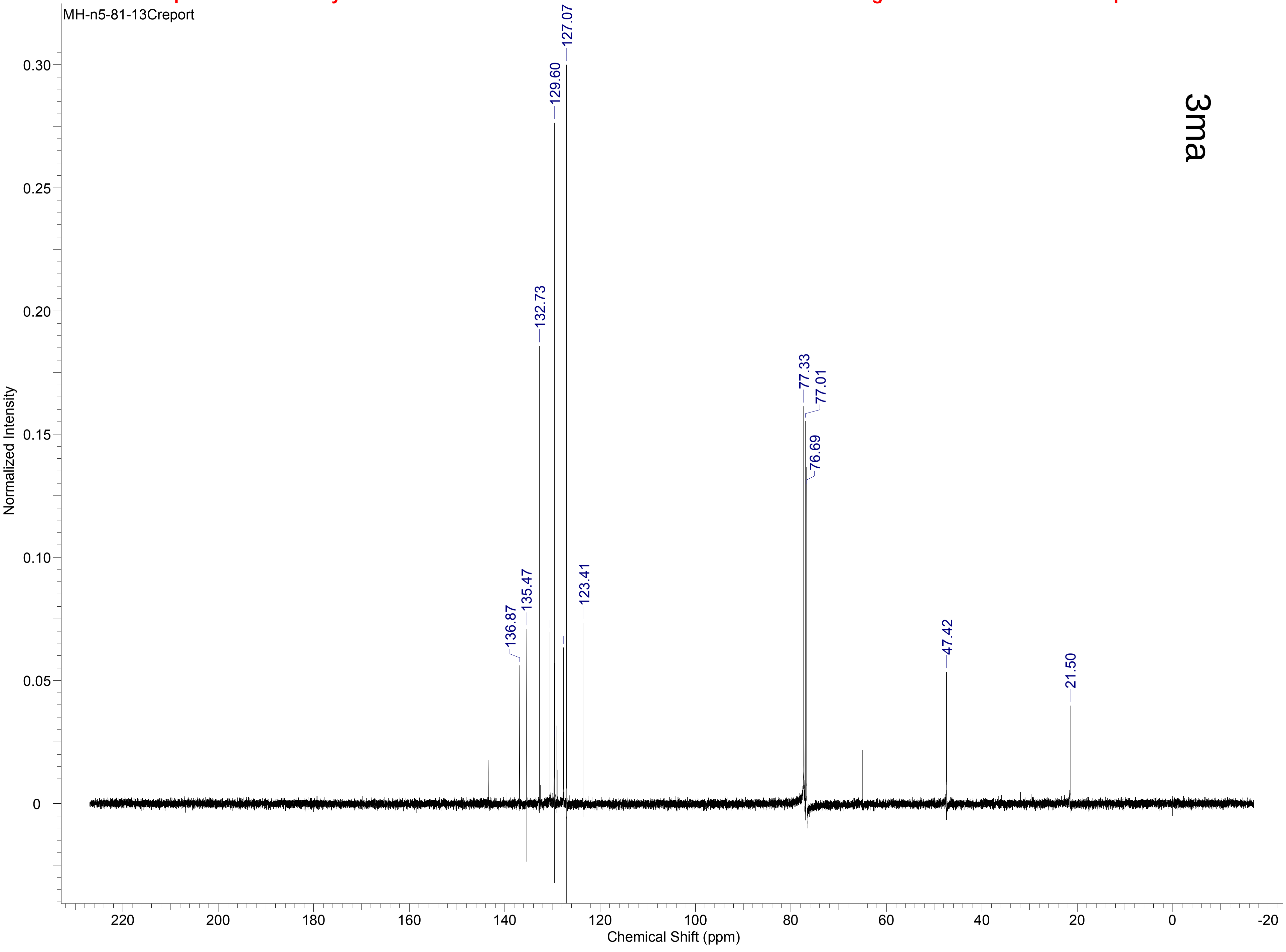

This report was created by ACD/NMR Processor Academic Edition. For more information go to www.acdlabs.com/nmrproc/

3bb

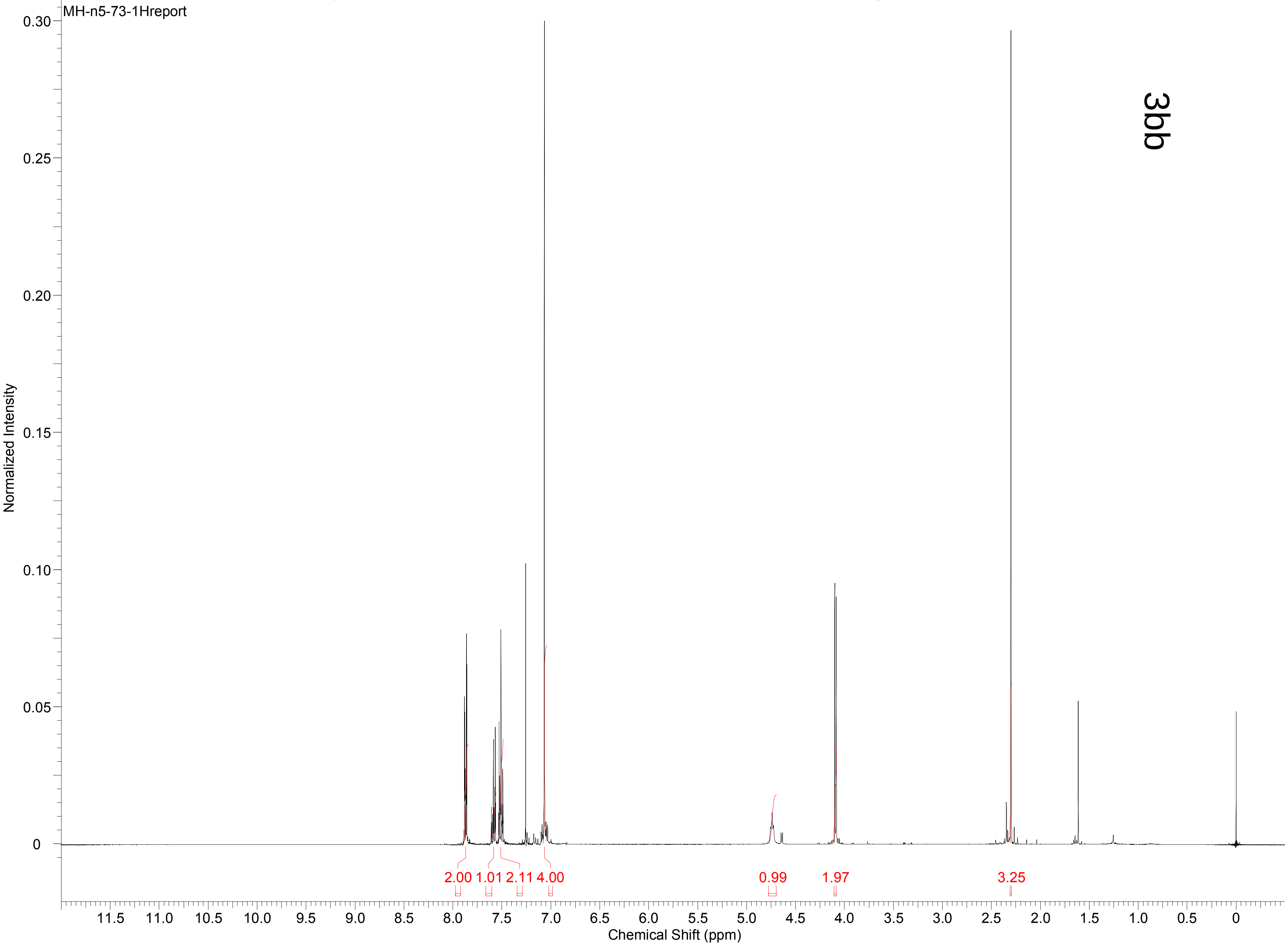

This report was created by ACD/NMR Processor Academic Edition. For more information go to www.acdlabs.com/nmrproc/

3bb

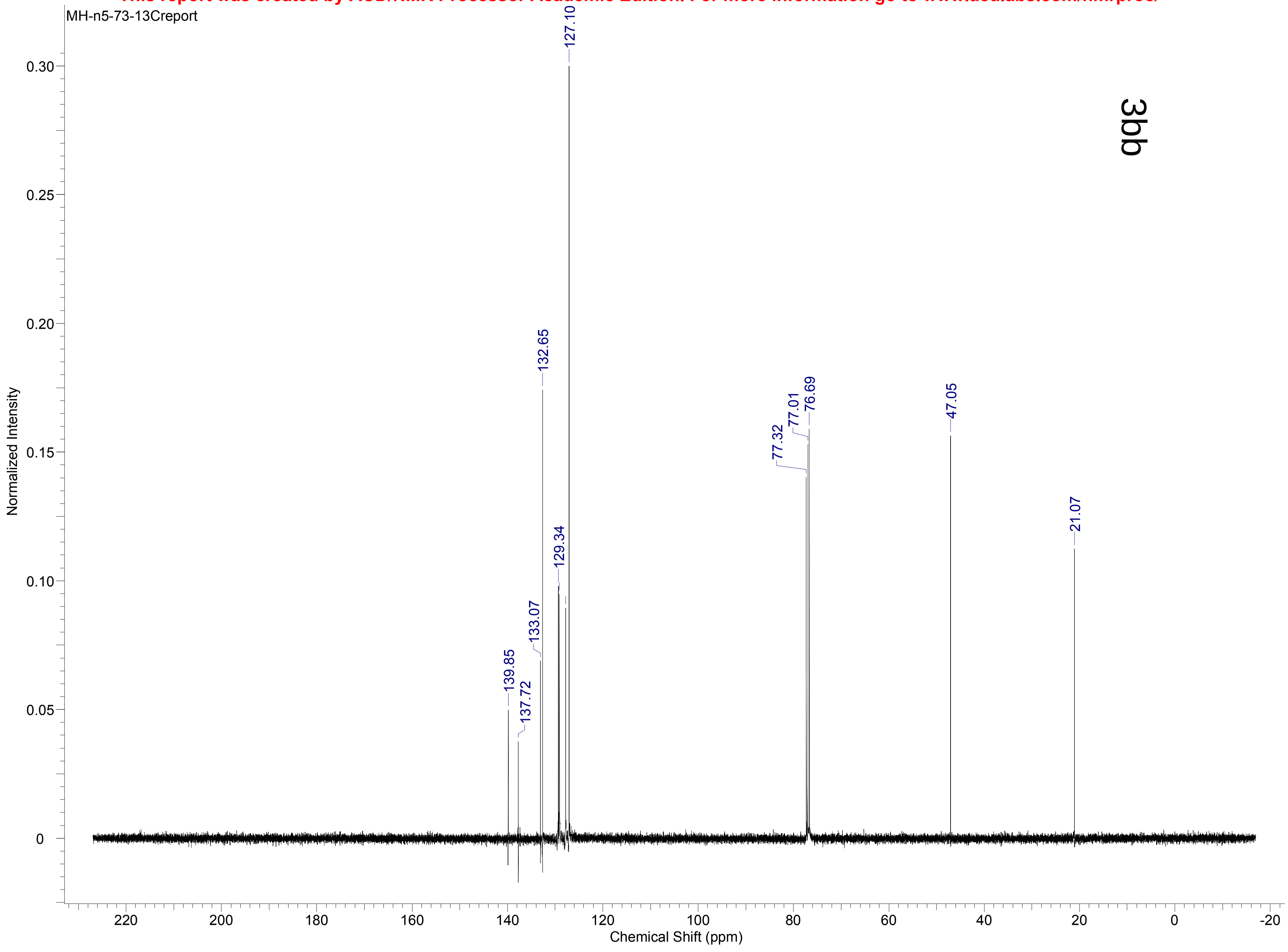

This report was created by ACD/NMR Processor Academic Edition. For more information go to www.acdlabs.com/nmrproc/

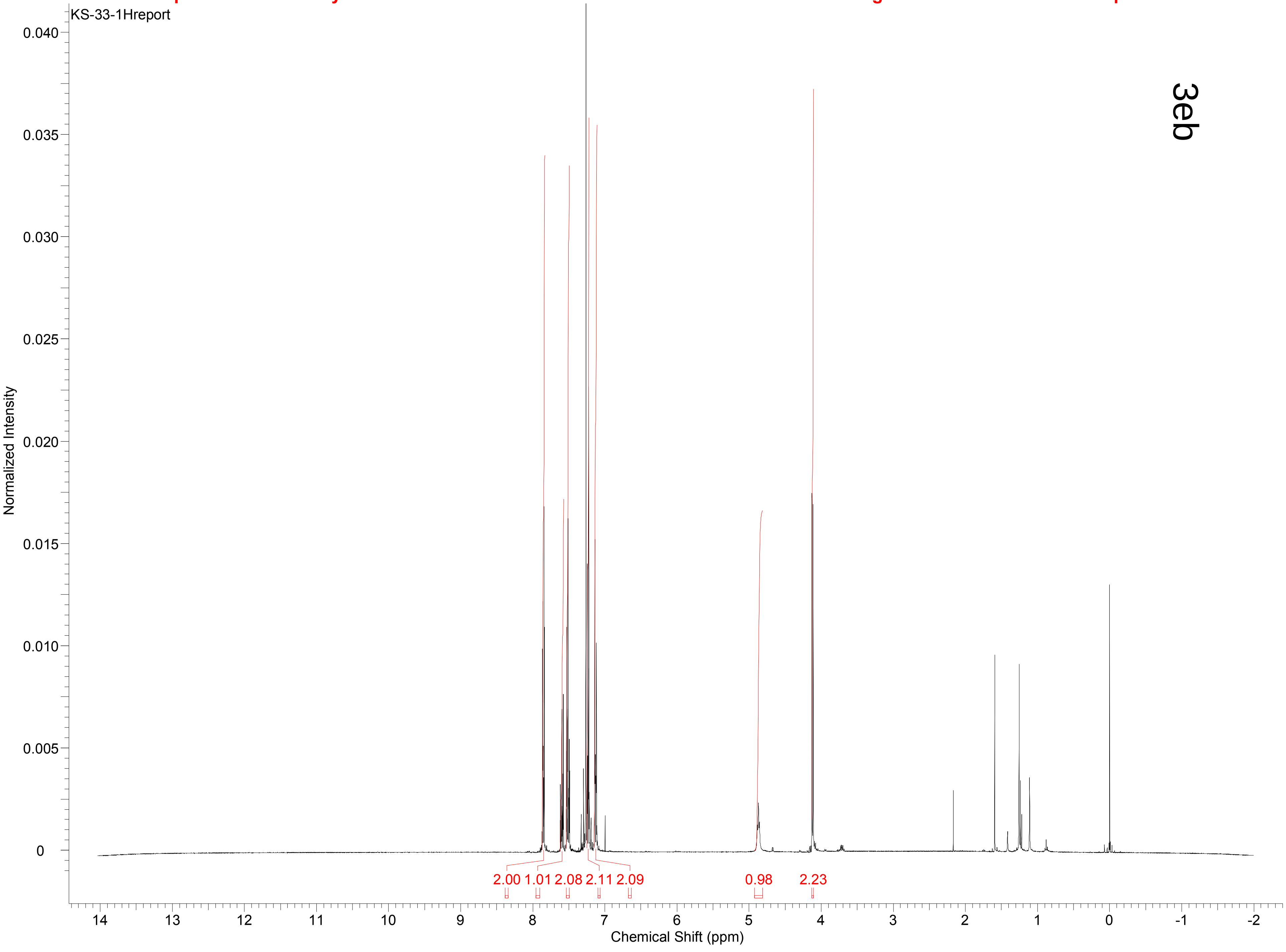

This report was created by ACD/NMR Processor Academic Edition. For more information go to www.acdlabs.com/nmrproc/

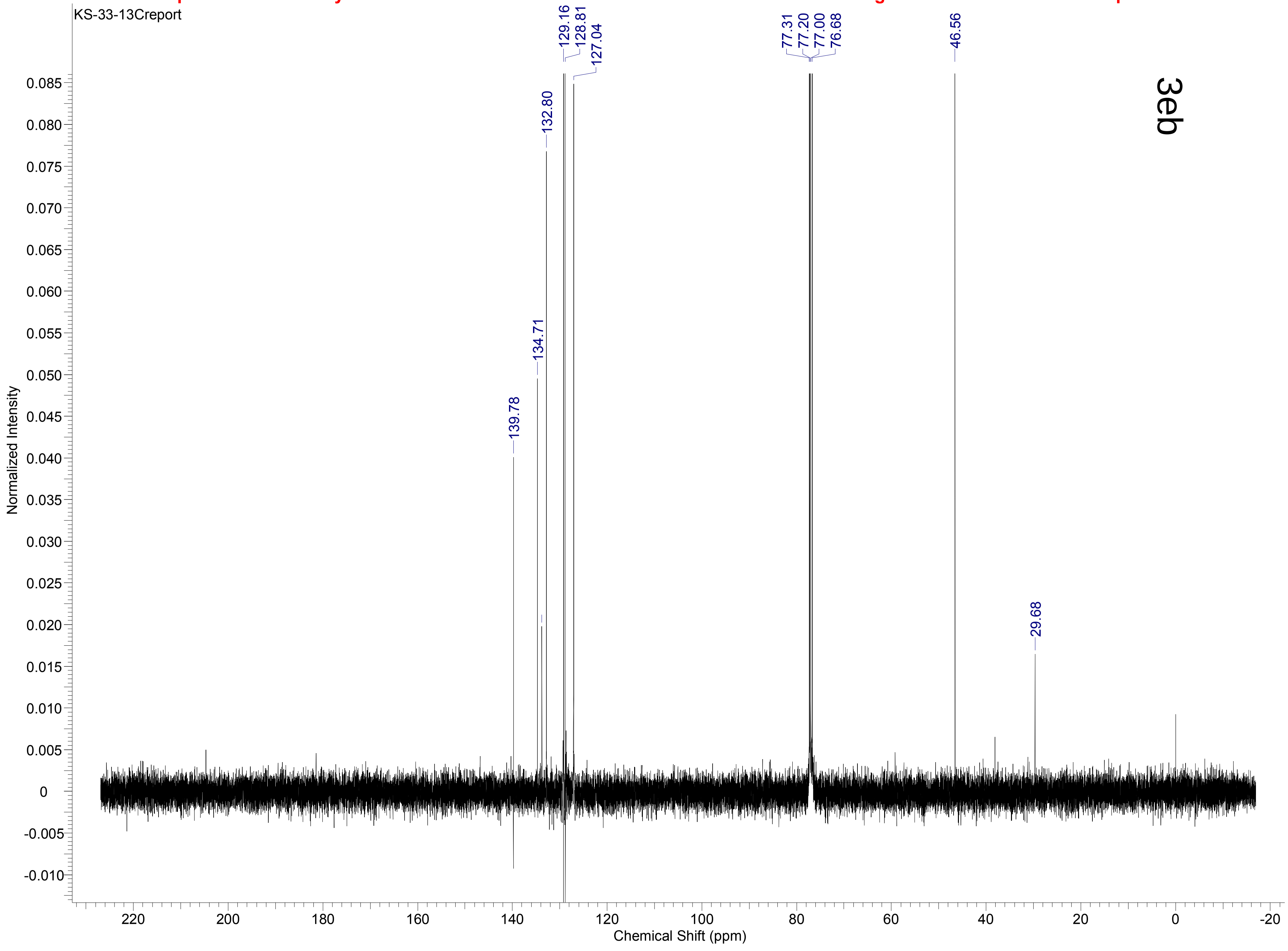

This report was created by ACD/NMR Processor Academic Edition. For more information go to www.acdlabs.com/nmrproc/

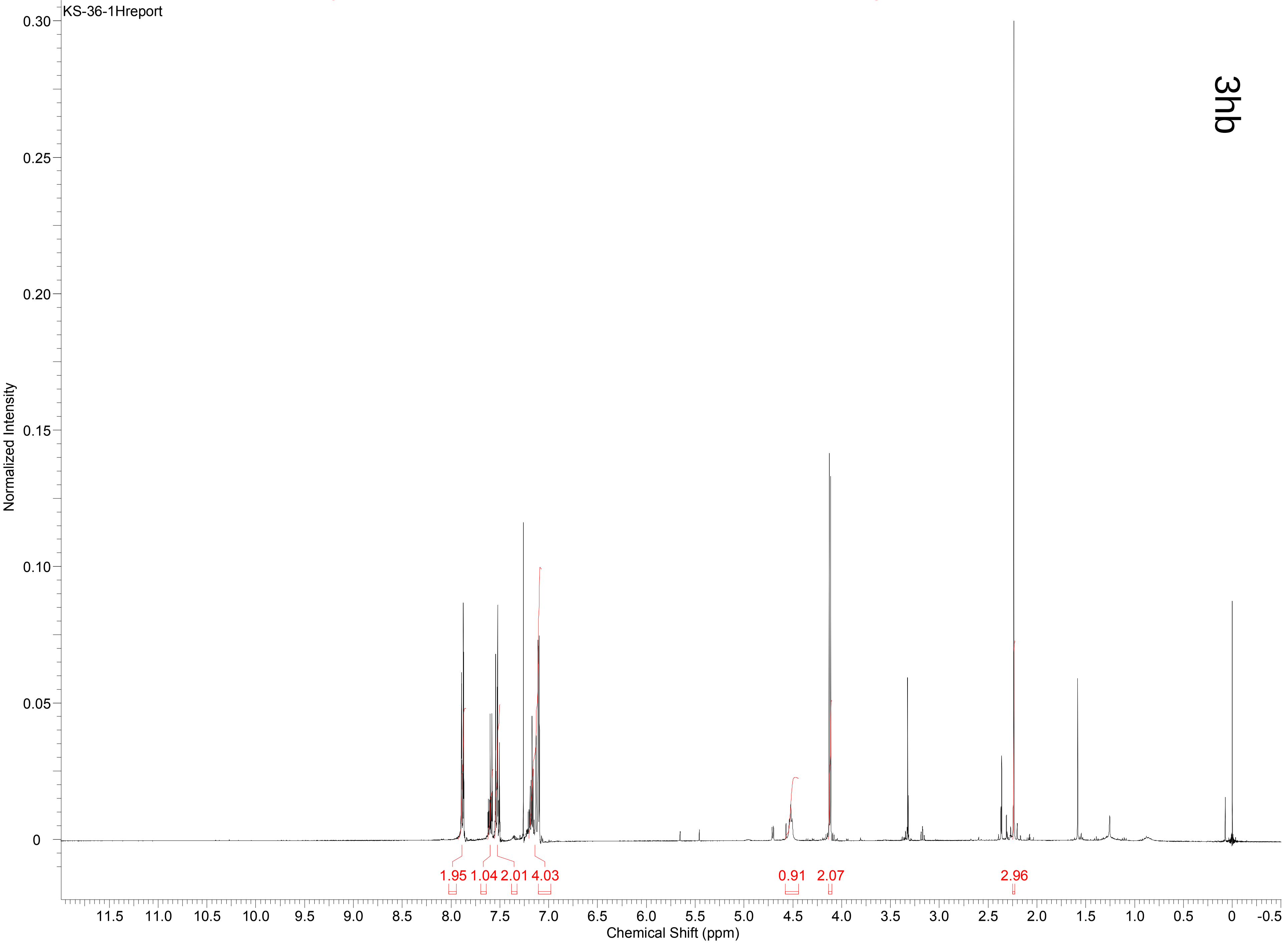

This report was created by ACD/NMR Processor Academic Edition. For more information go to www.acdlabs.com/nmrproc/

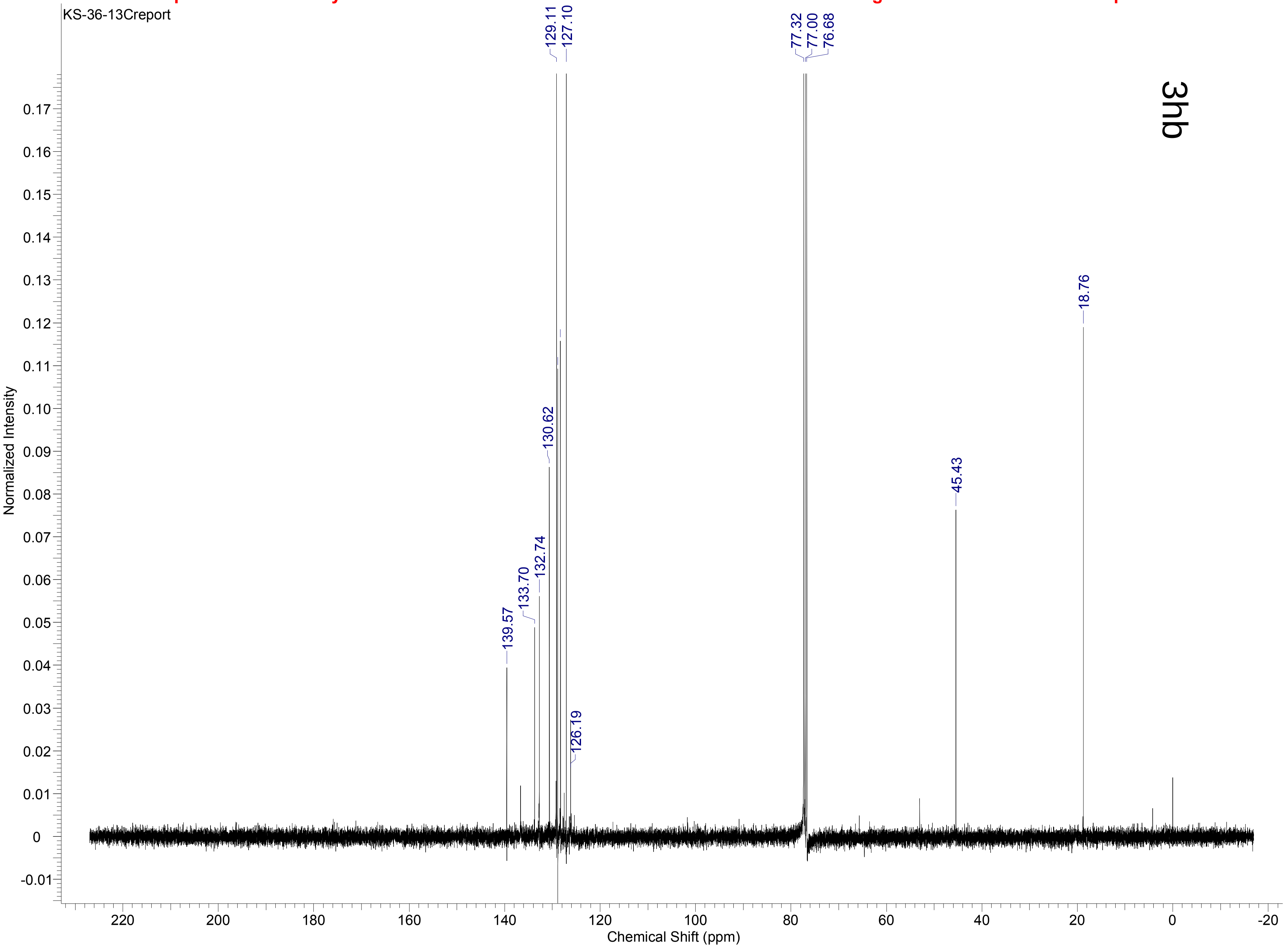

This report was created by ACD/NMR Processor Academic Edition. For more information go to www.acdlabs.com/nmrproc/

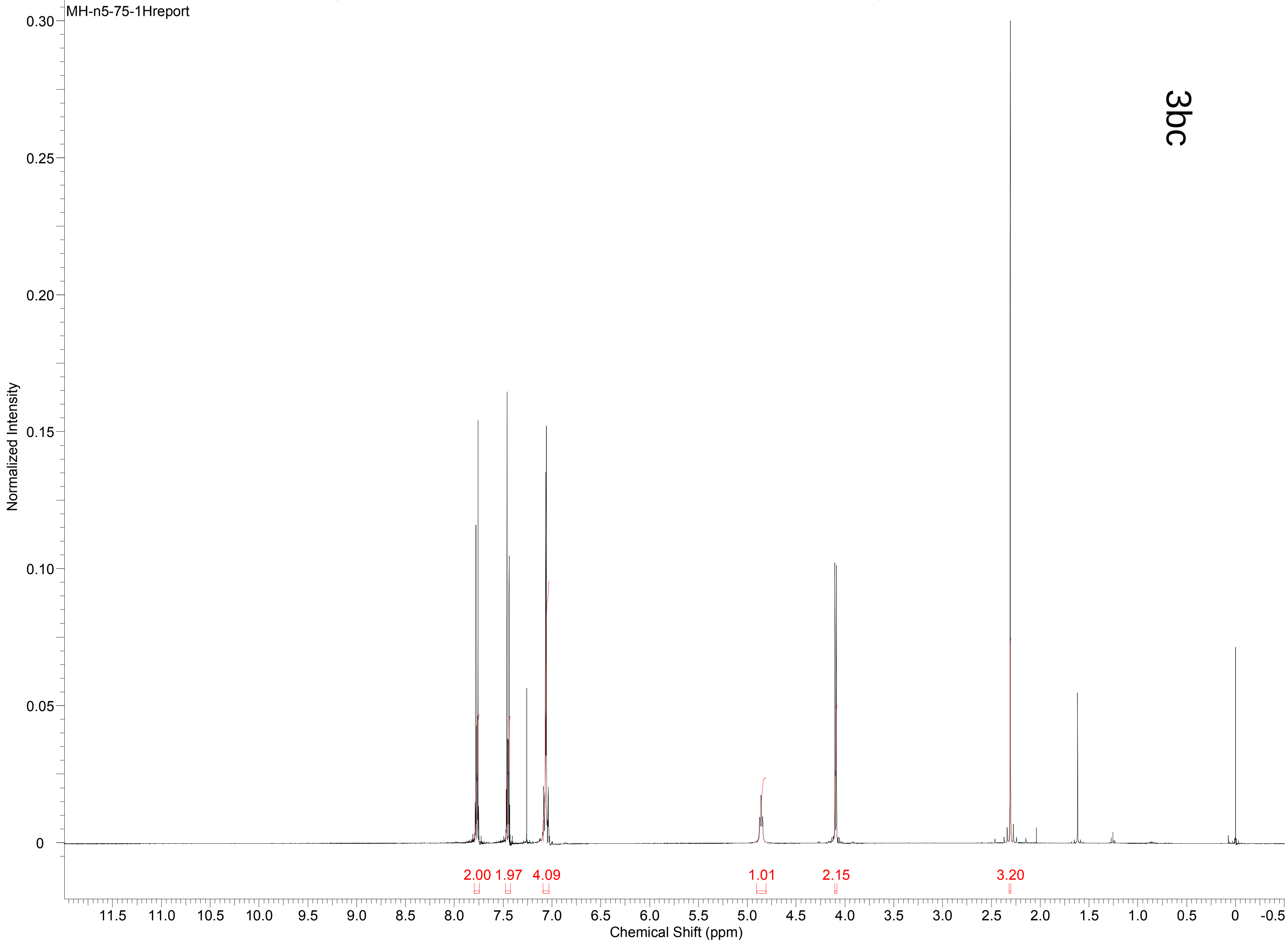

This report was created by ACD/NMR Processor Academic Edition. For more information go to www.acdlabs.com/nmrproc/

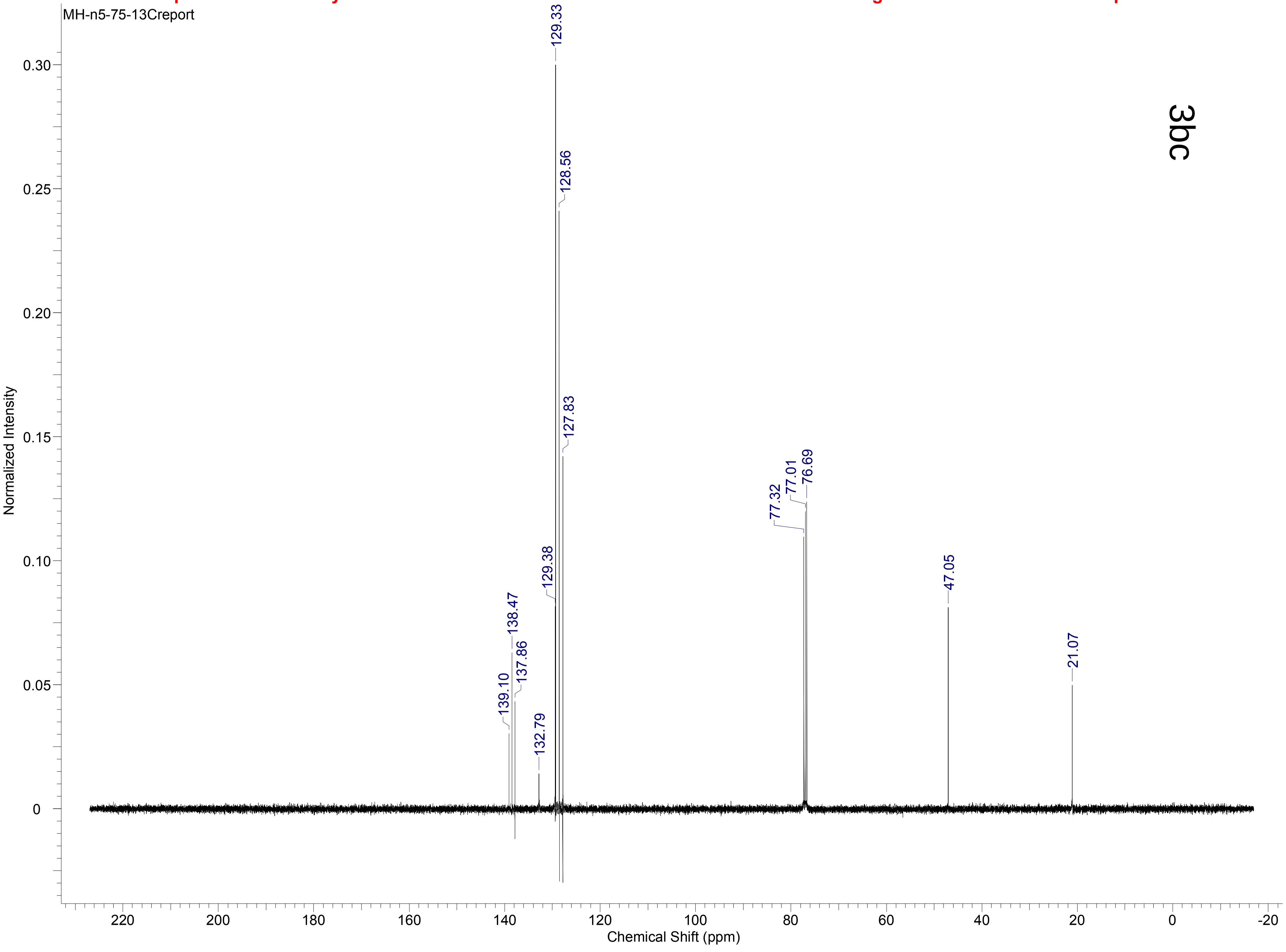

This report was created by ACD/NMR Processor Academic Edition. For more information go to www.acdlabs.com/nmrproc/

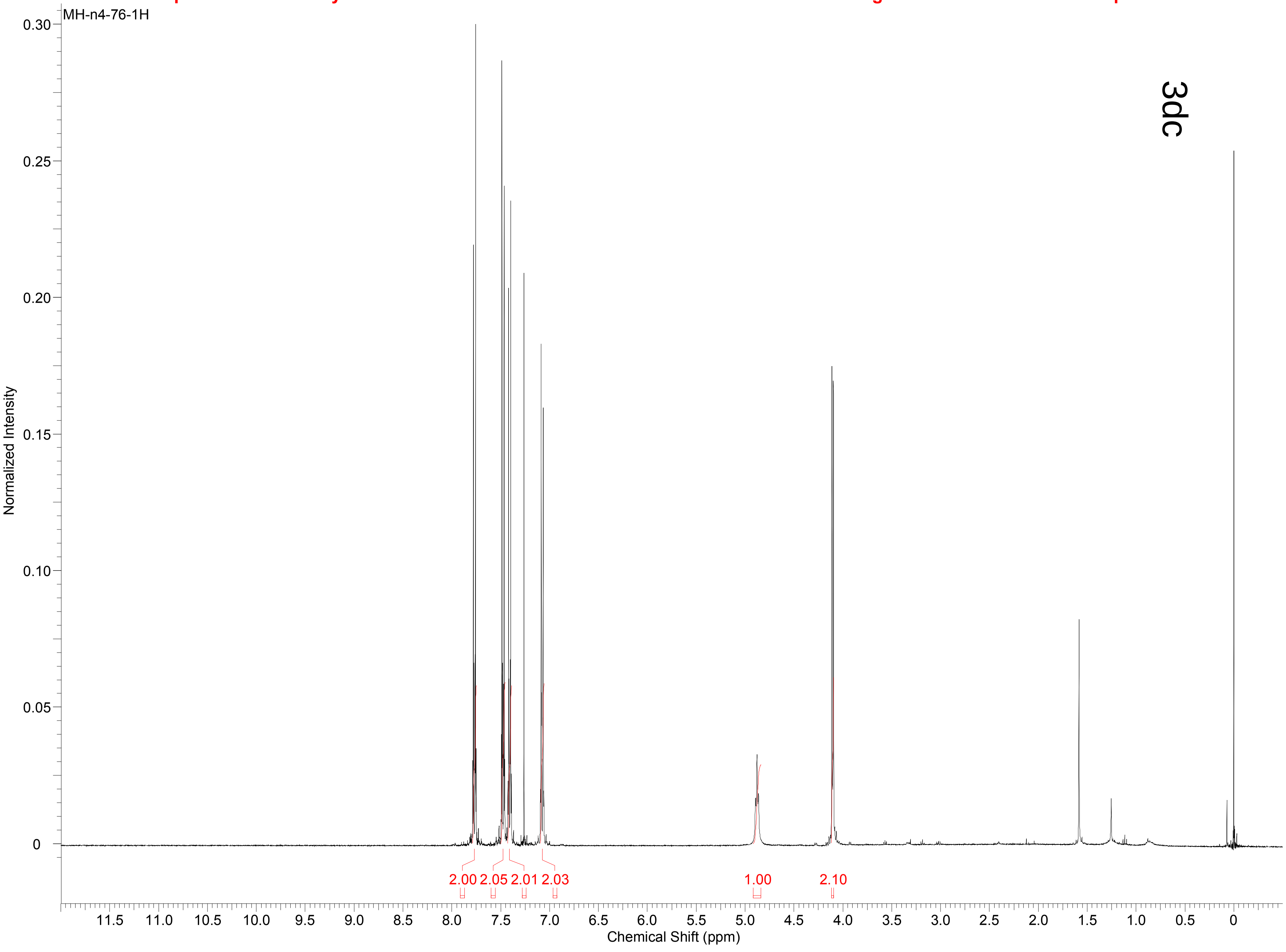

This report was created by ACD/NMR Processor Academic Edition. For more information go to www.acdlabs.com/nmrproc/

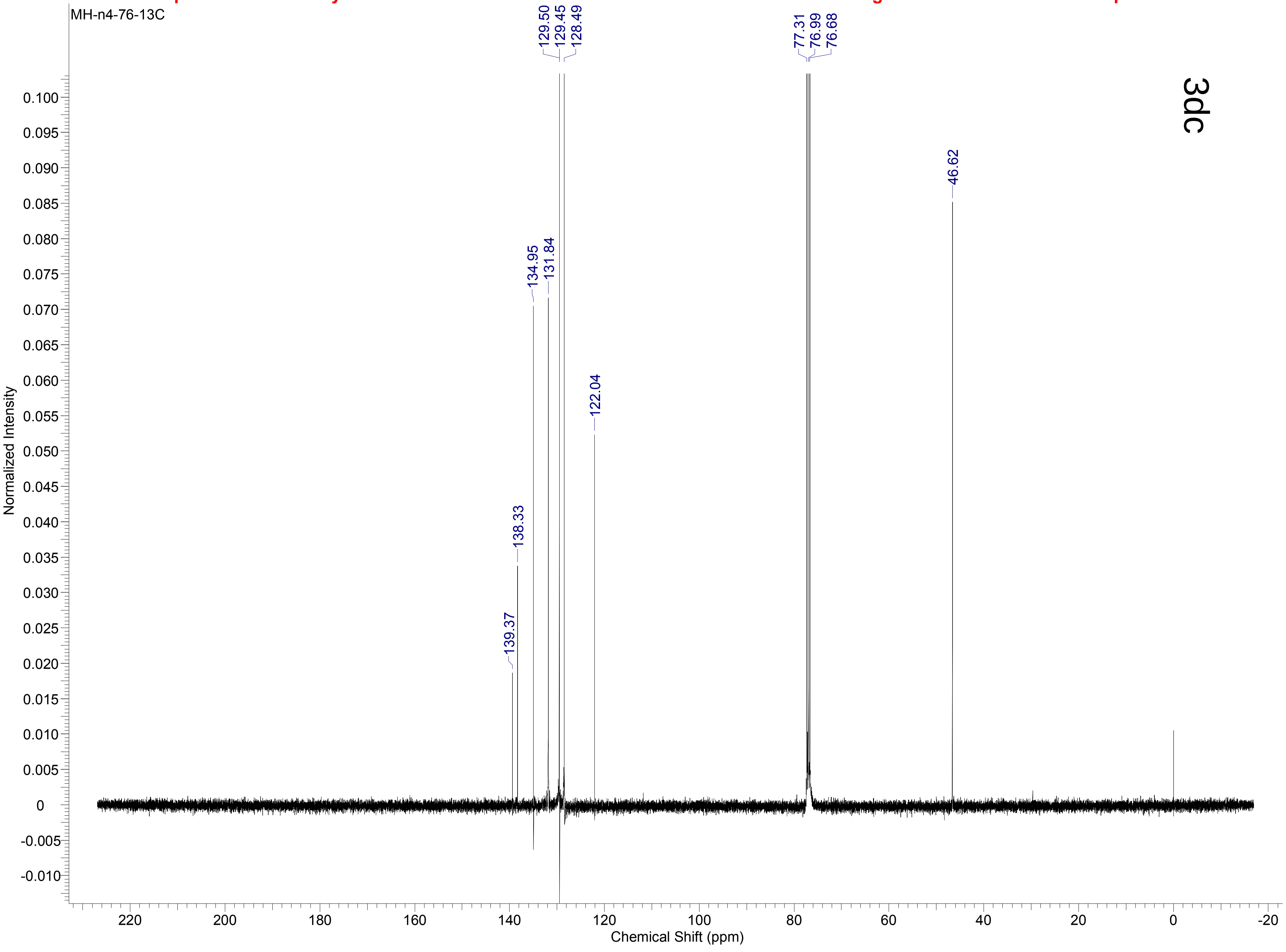

This report was created by ACD/NMR Processor Academic Edition. For more information go to www.acdlabs.com/nmrproc/

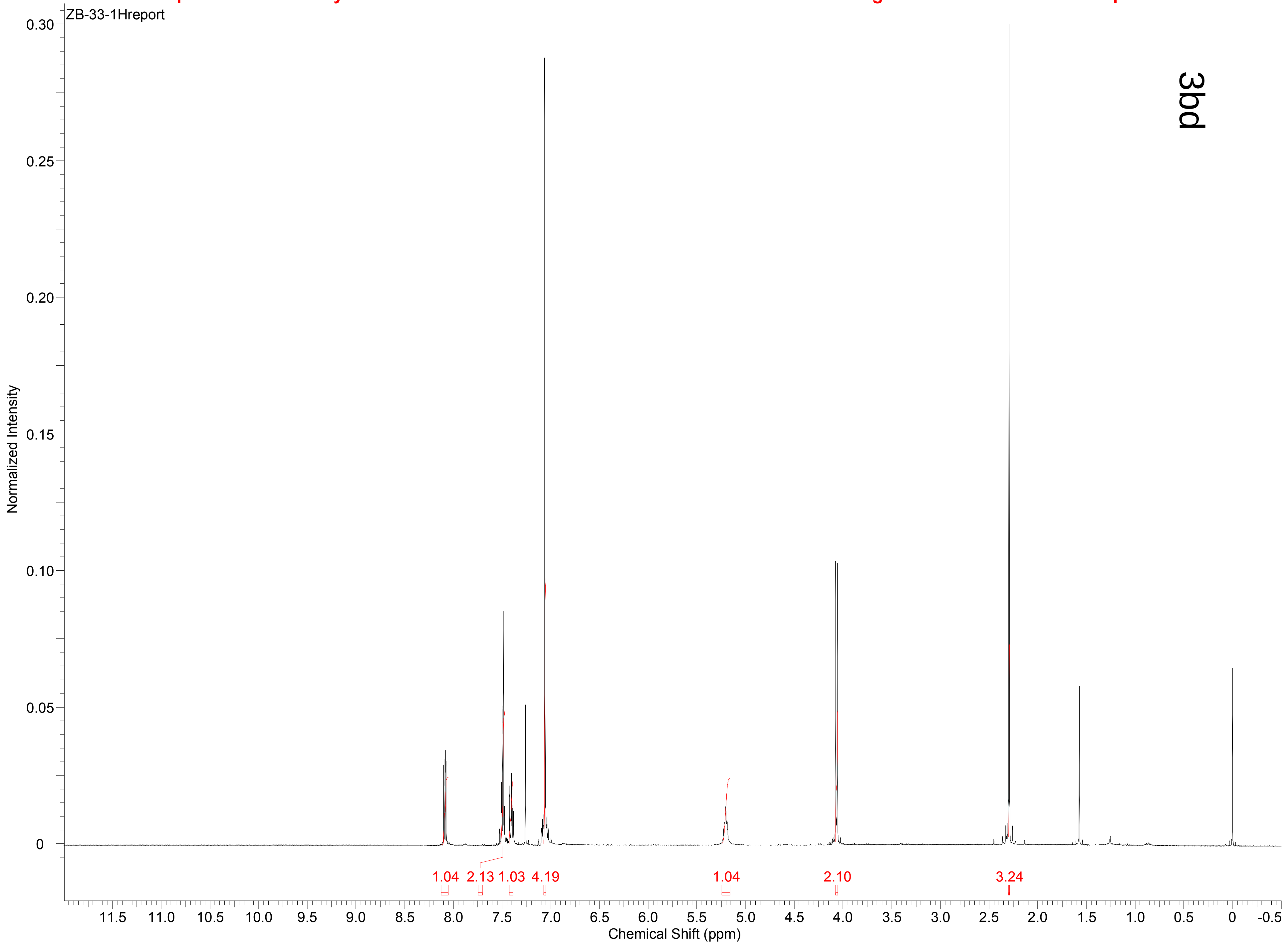

This report was created by ACD/NMR Processor Academic Edition. For more information go to www.acdlabs.com/nmrproc/

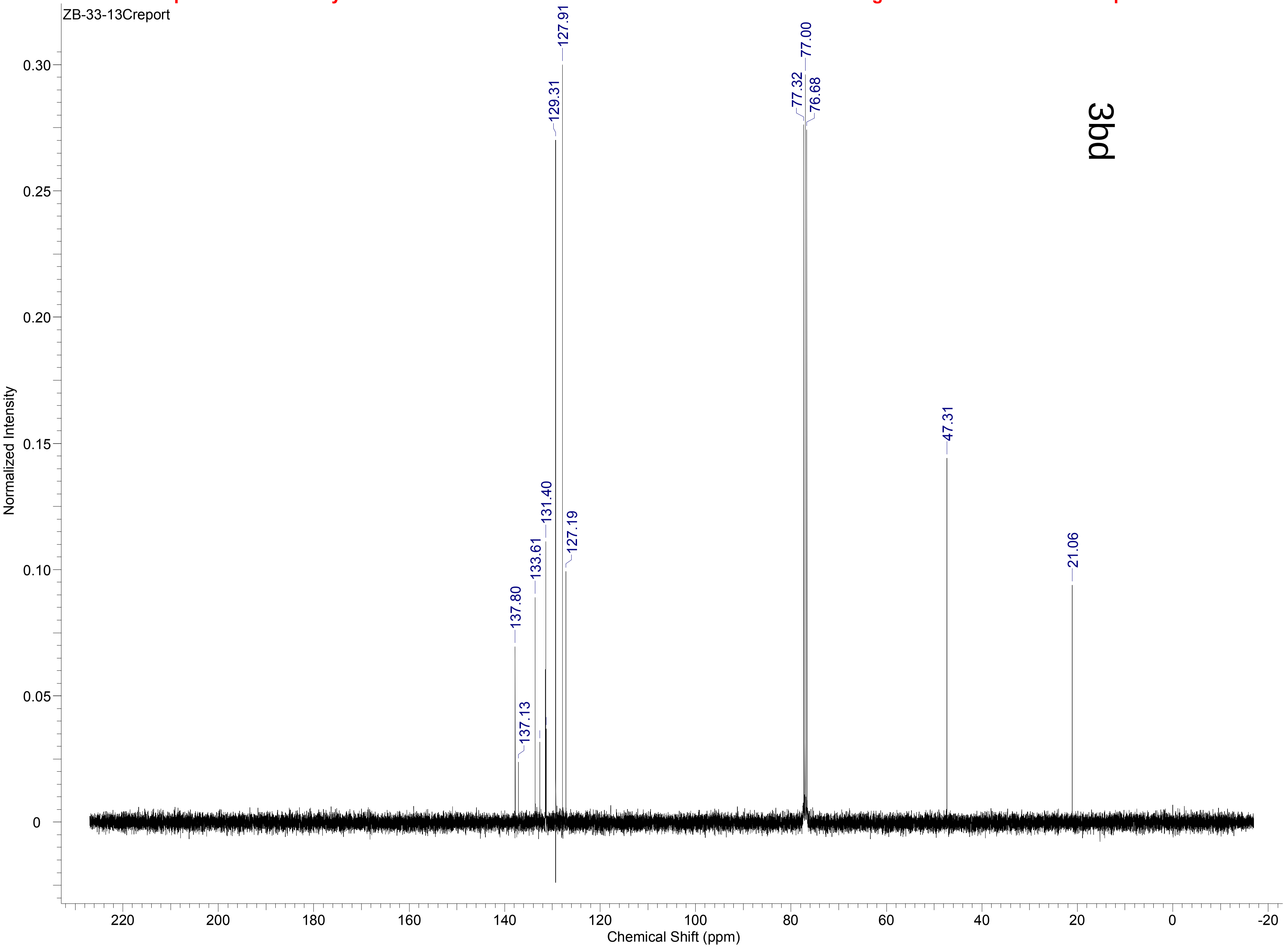

This report was created by ACD/NMR Processor Academic Edition. For more information go to www.acdlabs.com/nmrproc/

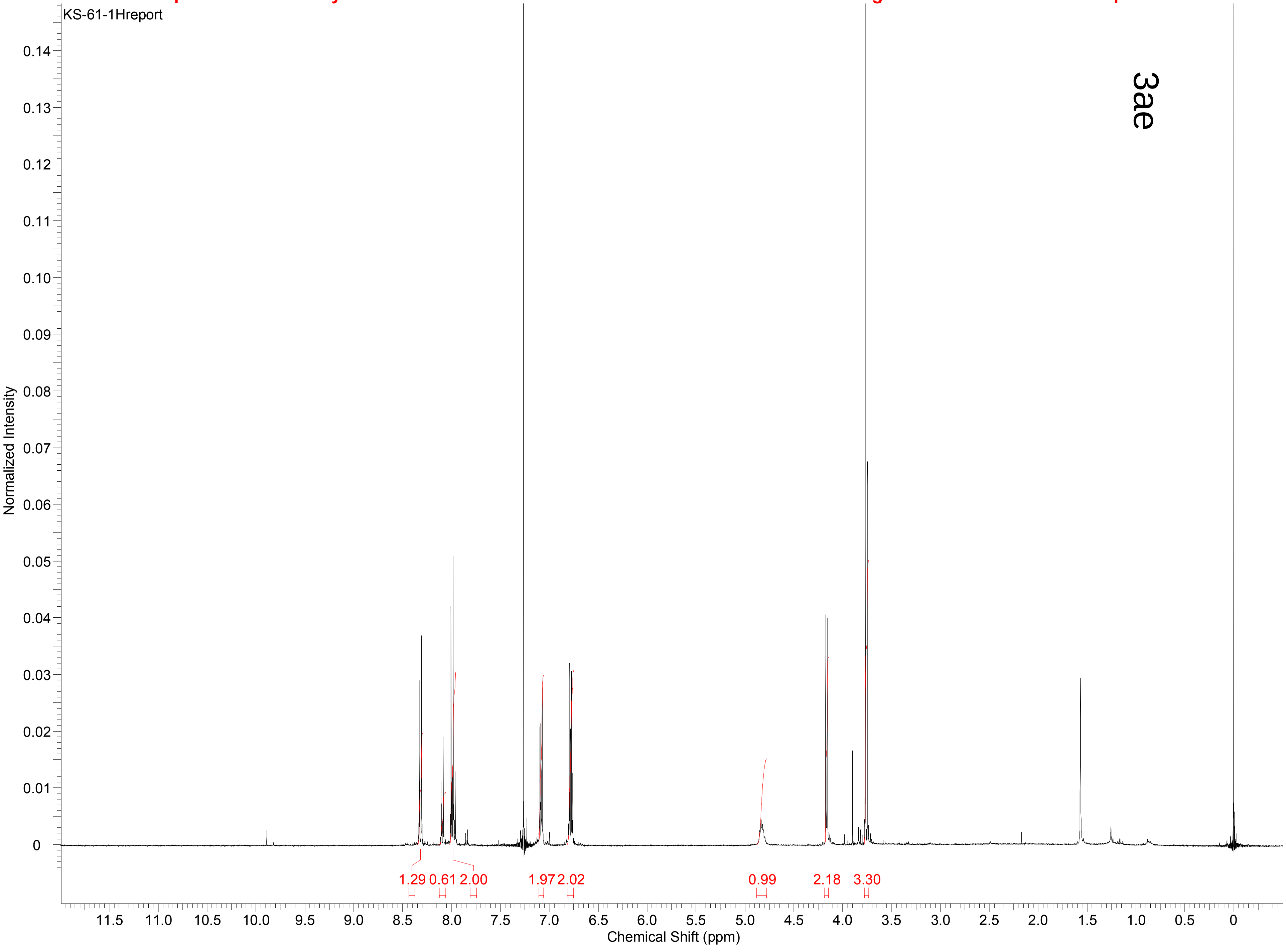

This report was created by ACD/NMR Processor Academic Edition. For more information go to www.acdlabs.com/nmrproc/

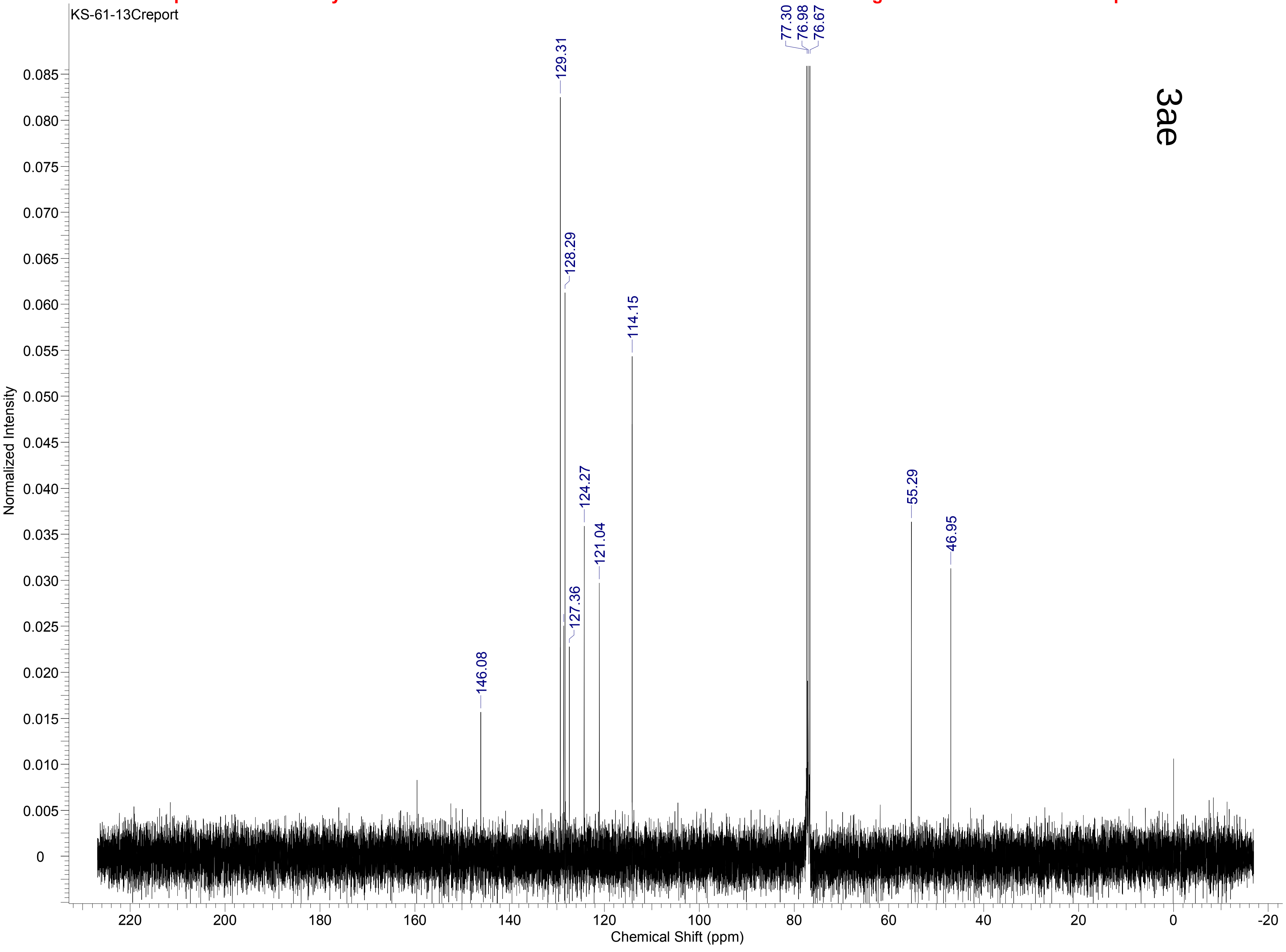

This report was created by ACD/NMR Processor Academic Edition. For more information go to www.acdlabs.com/nmrproc/

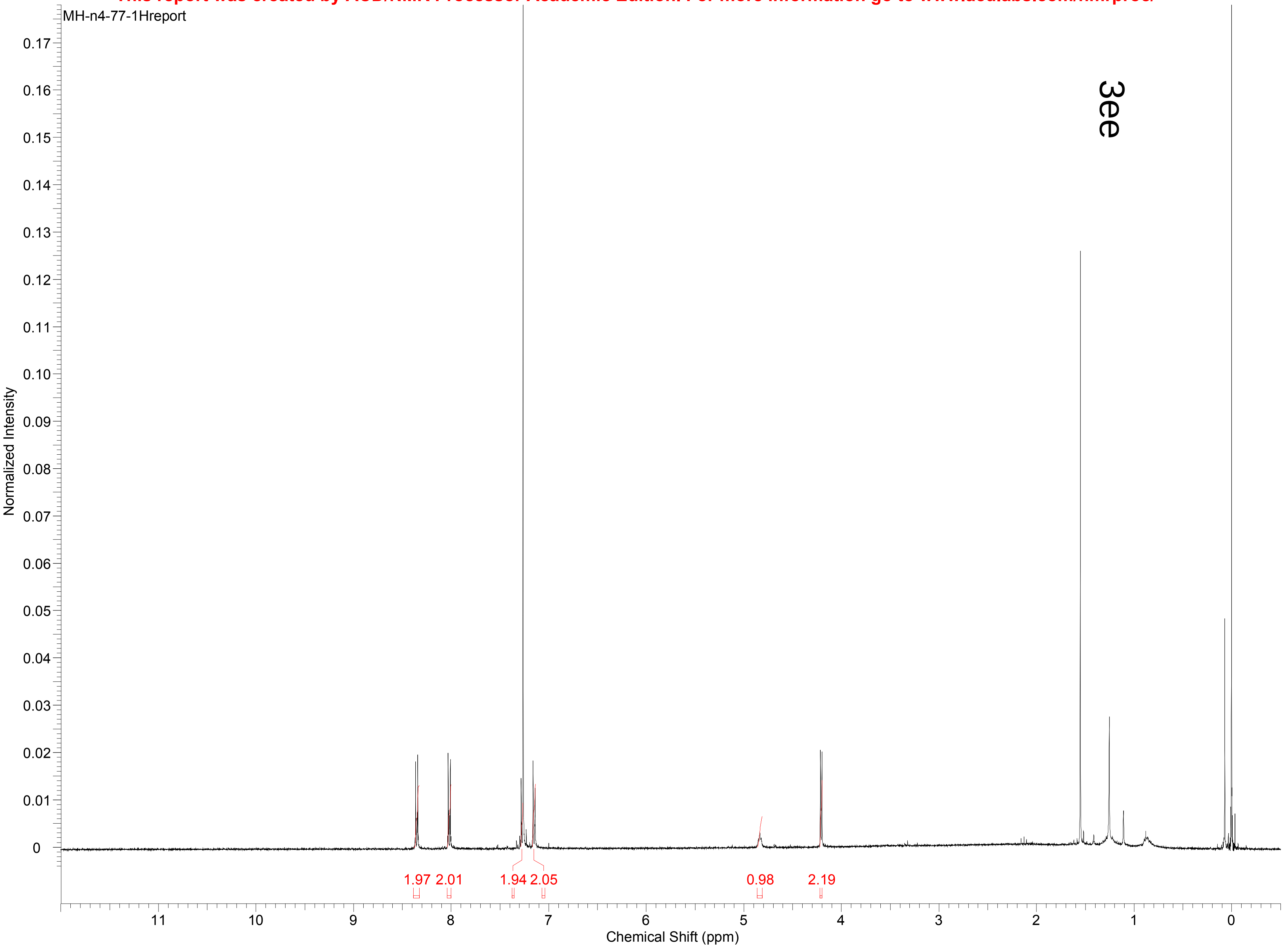

This report was created by ACD/NMR Processor Academic Edition. For more information go to www.acdlabs.com/nmrproc/

3ee

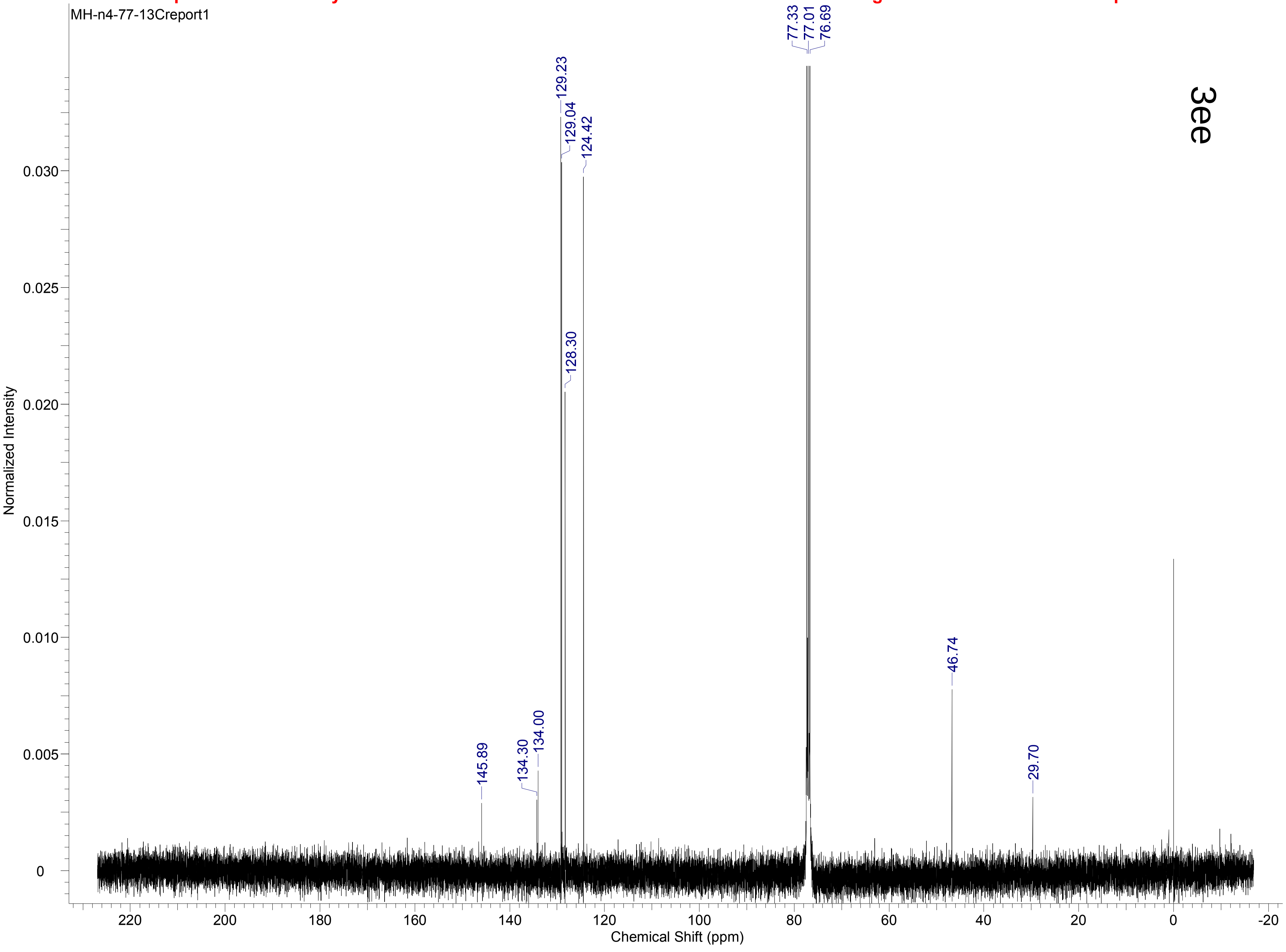

This report was created by ACD/NMR Processor Academic Edition. For more information go to www.acdlabs.com/nmrproc/

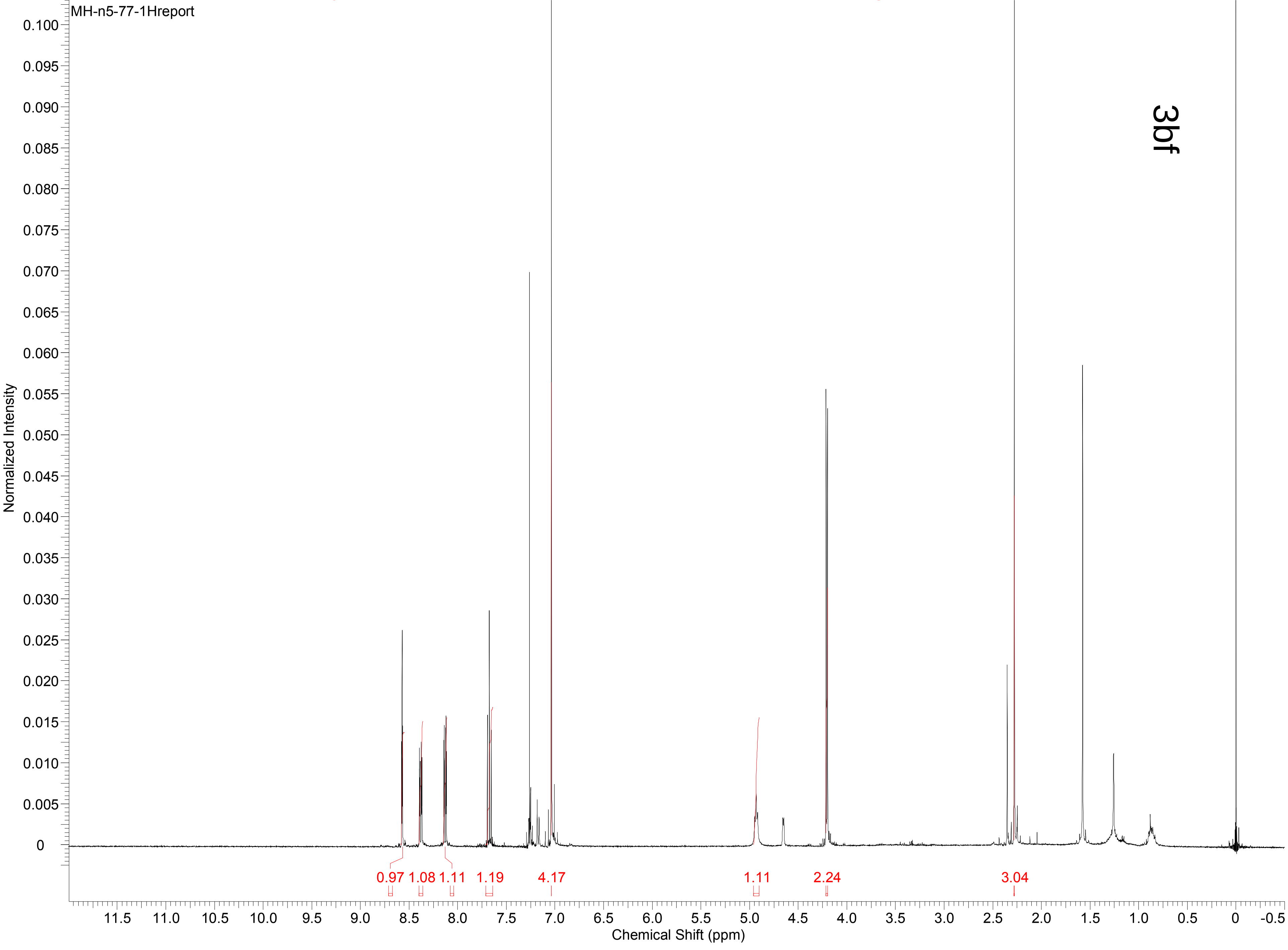

This report was created by ACD/NMR Processor Academic Edition. For more information go to www.acdlabs.com/nmrproc/

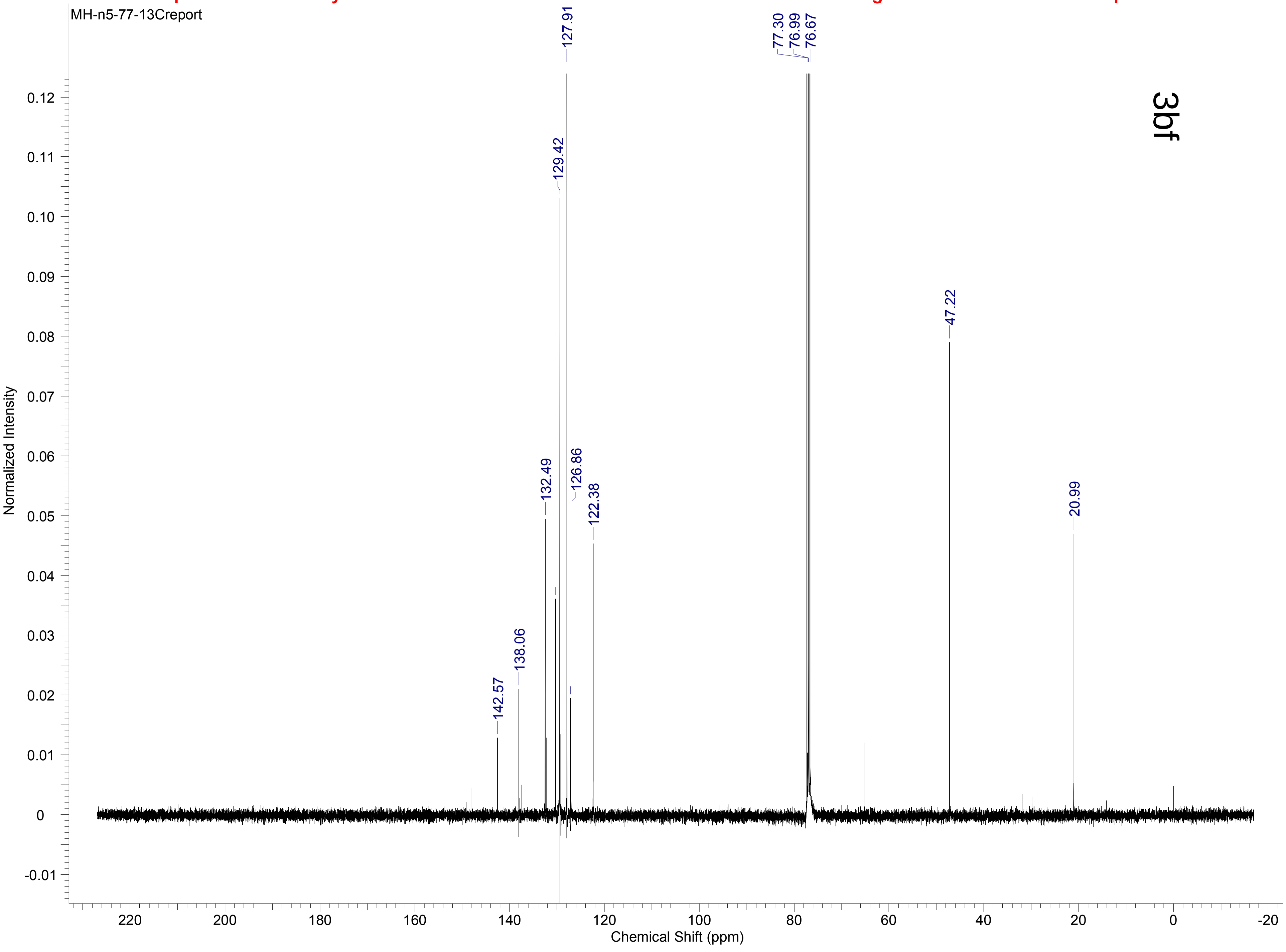

This report was created by ACD/NMR Processor Academic Edition. For more information go to www.acdlabs.com/nmrproc/

5

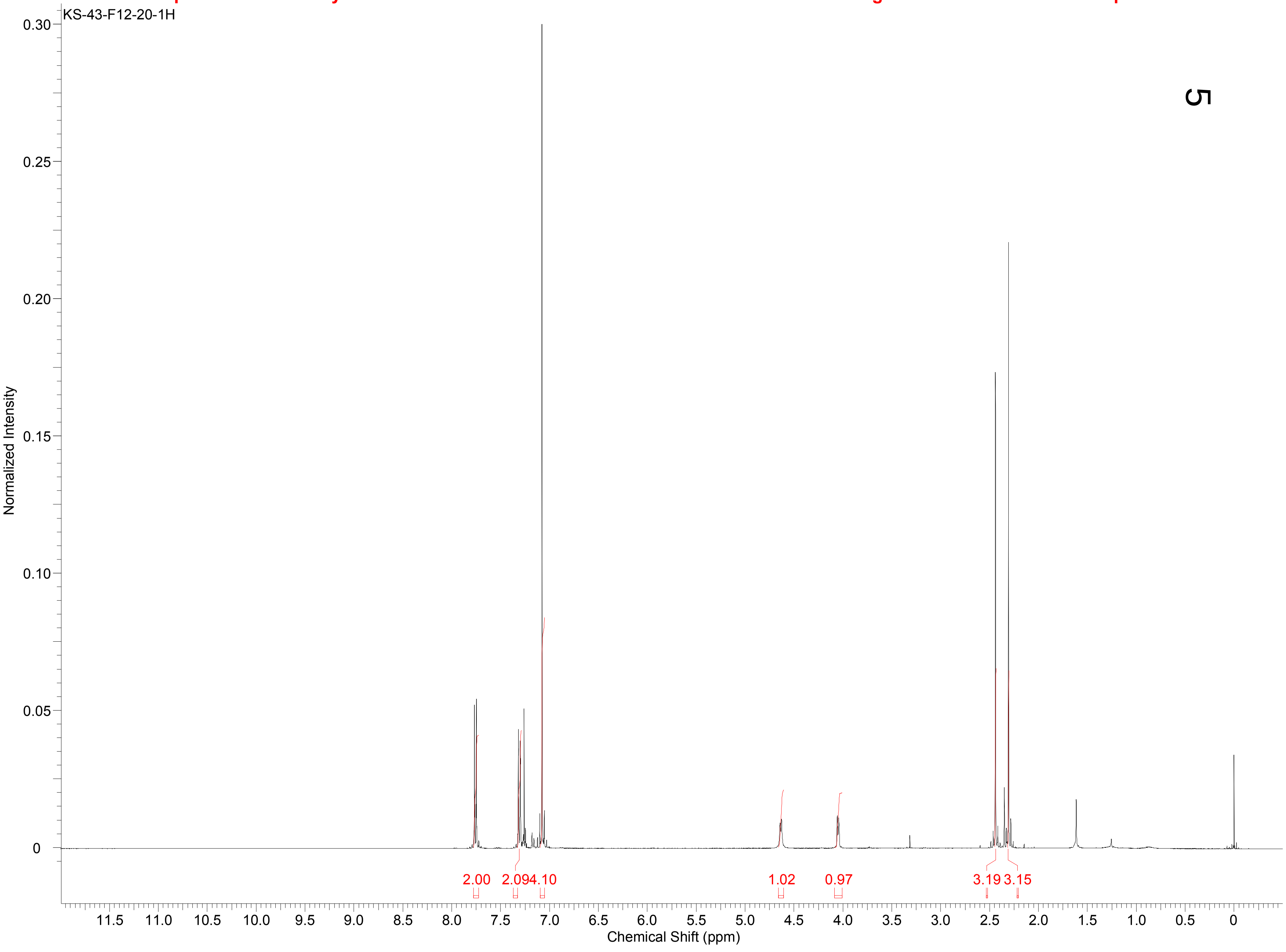

This report was created by ACD/NMR Processor Academic Edition. For more information go to www.acdlabs.com/nmrproc/

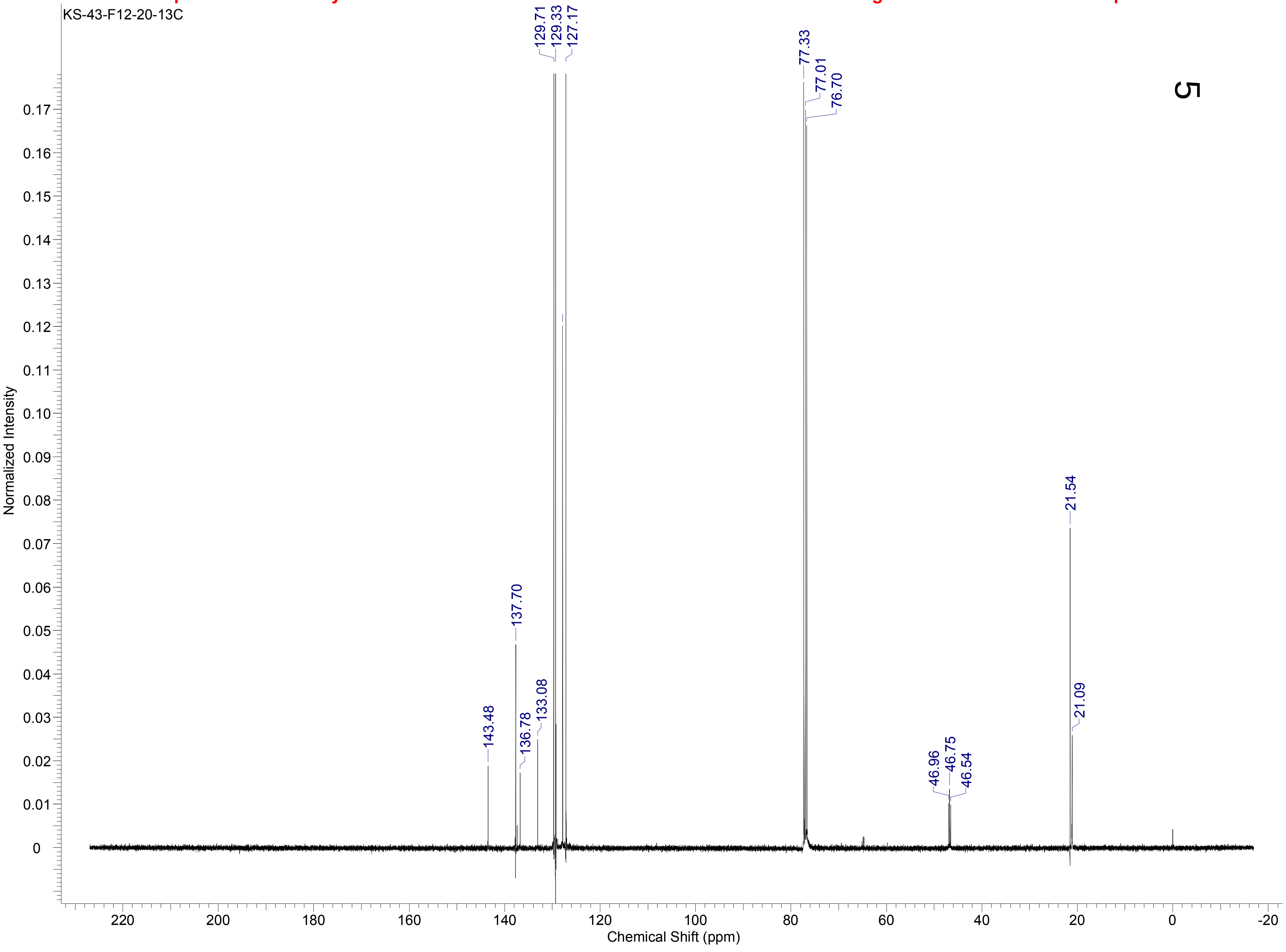

Supplement: Supplementary file 1 [file molecules-23-01838-s001.pdf]
